# Taming Flexibility: Synergistic Pore and Polarity Engineering in a MOF for High‐Efficiency Xe/Kr Separation

**DOI:** 10.1002/advs.76155

**Published:** 2026-06-15

**Authors:** Tao Zhao, Xue Wang, Youjin Gong, Siqi Dong, Shunshun Xiong, Hui Xu, Aziz Bakhtiyarovich Ibragimov, Thamraa Alshahrani, He Zheng, Lin Li, Junkuo Gao

**Affiliations:** ^1^ China‐Uzbekistan Joint Laboratory on Advanced Porous Materials State Key Laboratory of Bio‐based Fiber Materials School of Materials Science and Engineering Zhejiang Sci‐Tech University Hangzhou P. R. China; ^2^ Institute of Nuclear Physics and Chemistry China Academy of Engineering Physics Mianyang Sichuan P. R. China; ^3^ Key Laboratory of Rare Earth Optoelectronic Materials and Devices of Zhejiang Province Institute of Optoelectronic Materials and Devices College of Optical and Electronic Technology China Jiliang University Hangzhou P. R. China; ^4^ Institute of General and Inorganic Chemistry Uzbekistan Academy of Sciences Tashkent Uzbekistan; ^5^ Department of Physics College of Science Princess Nourah bint Abdulrahman University Riyadh Saudi Arabia; ^6^ Zhejiang Key Laboratory of Advanced Catalysis and Adsorption Materials Key Laboratory of the Ministry of Education for Advanced Catalysis Materials College of Chemistry and Materials Science Zhejiang Normal University Jinhua P. R. China

**Keywords:** metal‐organic frameworks, mixed ligand, partial polarization, pore modification, Xe/Kr separation

## Abstract

The separation of xenon (Xe) and krypton (Kr), two inert gases with nearly identical physicochemical properties, remains a formidable industrial challenge. Flexible metal–organic frameworks (MOFs) offer guest‐specific recognition via structural responsiveness; however, their application in low‐concentration separations is limited by intrinsic flexibility and high gate‐opening pressures, as exemplified by ZIF‐7. Herein, we propose and experimentally substantiate a synergistic regulation strategy that concurrently modulates the pore size and polarity of the framework, thereby overcoming the intrinsic flexibility of MOFs and enabling exceptional Xe/Kr separation performance. By incorporating 20% chlorine into the framework (ZIF‐7‐Cl(20)), the pore environment is effectively polarized, promoting highly selective Xe recognition while preserving adsorption capacity. The optimized material exhibits an IAST selectivity of 30.8 for Xe/Kr (20/80, v/v). Grand Canonical Monte Carlo simulations reveal strong Xe─Cl interactions as the origin of the enhanced selectivity. Dynamic breakthrough experiments further demonstrate excellent separation performance, affording high‐purity Kr (>99.9%) with a productivity of 129 L kg^−^^1^ and a retention time of 84 min g^−^^1^. This work provides a generalizable strategy for converting flexible MOFs into high‐performance adsorbents for challenging gas separations.

## Graphical abstract

Through a mixed‐ligand strategy that precisely regulates pore size and framework polarity, the Xe adsorption behavior is transformed from flexible to near‐rigid. ZIF‐7‐Cl(20) achieves sensitive recognition, efficient capture, and high selectivity for Xe, enabling high‐efficiency separation from Xe/Kr mixtures.

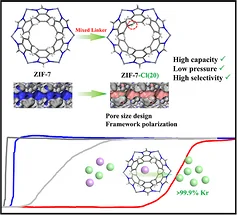

## 1 Introduction

The capture and separation of xenon (Xe) and krypton (Kr) are of critical importance in the industrial production of inert gases [1, 2, 3]. Owing to their unique physicochemical properties, high‐purity Xe and Kr hold significant application value in various fields, including medical imaging, anesthesia, welding, and commercial lighting [4, 5]. Although an Xe/Kr (20/80, v/v) mixture can be obtained as a by‐product during cryogenic air separation [6], obtaining high‐purity Xe and Kr still relies on further traditional cryogenic distillation [7, 8]. Consequently, the development of more energy‐efficient and cost‐effective methods for separating inert gases is both urgent and of strategic importance [9]. The use of solid porous adsorbents for the highly efficient separation of Xe/Kr mixtures under near‐ambient temperature and pressure conditions presents considerable potential for energy savings and cost reduction [3, 10, 11]. This approach demonstrates strong competitiveness and is regarded as a highly promising alternative strategy for the separation and purification of Xe/Kr mixtures.

However, the highly efficient separation of Xe and Kr represents a considerable challenge owing to their nearly identical physicochemical properties, including similar kinetic diameters (Xe: 4.047 Å, Kr: 3.655 Å) and zero dipole and quadrupole moments [12, 13]. Conventional porous adsorbents such as activated carbon and zeolites, while capable of achieving partial Xe/Kr separation under ambient conditions, typically exhibit limited Xe uptake capacity and selectivity, which severely restricts their practical separation efficiency [14, 15]. Metal‐organic frameworks (MOFs) have emerged as promising candidates for gas separation applications due to their highly designable pore architectures and tunable surface functionalities [16, 17, 18]. In particular, flexible MOFs, a distinctive subclass, demonstrate stimuli‐responsive “breathing” or “gate‐opening” behavior upon interaction with specific guest molecules [19, 20]. Such structural transitions endow them with markedly distinct adsorption affinities toward different gases, rendering them attractive for challenging separation processes [21, 22]. Nevertheless, the gate‐opening pressure (P_go_) is often high and difficult to precisely tailor, especially for weakly interacting guests like Xe, rendering their practical application challenging [23].

A promising strategy to address this limitation is the mixed‐ligand approach, which involves the incorporation of a second isomorphic ligand into the parent framework without altering its overall topology [24]. By controlling the ratio of the secondary ligand, the framework flexibility and energetics can be tuned to modulate adsorption properties while maintaining structural integrity, thereby enhancing separation performance [25]. It should be noted, however, that excessive introduction of the secondary ligand may induce pronounced pore distortion, accumulated internal stress, and even degradation of crystallinity, ultimately leading to diminished adsorption capacity [26]. Moreover, certain secondary ligands may reduce the effective pore aperture, resulting in retarded gas diffusion kinetics or reduced accessibility to adsorption sites, which adversely affects the separation process [27]. Therefore, in the construction of flexible MOFs via mixed‐ligand functionalization, precise control over the secondary ligand content is crucial. An optimal degree of ligand substitution can effectively tailor the Xe/Kr adsorption selectivity while maintaining robust framework stability, thereby offering a viable and sophisticated materials design strategy for high‐performance Xe/Kr separation.

The mixed‐ligand approach offers a powerful strategy for precise engineering of pore architecture and chemical microenvironments in MOFs [27, 28]. Herein, we hypothesize that by incorporating a secondary ligand with both steric bulk and polarity (5‐ClBzIM) into the flexible ZIF‐7 framework, we can achieve a synergistic effect: the steric hindrance from the Cl atom can lower the gate‐opening pressure and rigidify the framework, while its electronegativity can create favorable polar sites to enhance Xe affinity (Xe: 40.44 × 10^−25^ cm^3^; Kr: 24.84 × 10^−25^ cm^3^) [29]. By incorporating a chlorine‐functionalized co‐linker into the flexible ZIF‐7 framework, we synthesized ZIF‐7‐Cl(x) materials with different linker ratios (x = molar percentage of 5‐ClBzIM). While pristine ZIF‐7 exhibits limited Xe/Kr separation capability due to its high gate‐opening pressure, optimal 5‐ClBzIM incorporation (x = 20) in ZIF‐7‐Cl(20) induces a polar pore environment with slightly reduced aperture, transforming the adsorption profile from pronounced flexible breathing toward near‐rigid adsorption behavior. This structural modification enables enhanced low‐pressure Xe uptake and superior Xe/Kr separation, demonstrating IAST and Henry's selectivity of 30.8 and 25.2, respectively, for Xe/Kr (20/80, v/v) mixtures. Breakthrough experiments confirm exceptional separation performance under dynamic conditions, while GCMC simulations reveal that localized polarization from Cl groups strengthens framework‐Xe interactions, providing mechanistic insight into the separation process.

## 2 Results and Discussion

**FIGURE 1:**
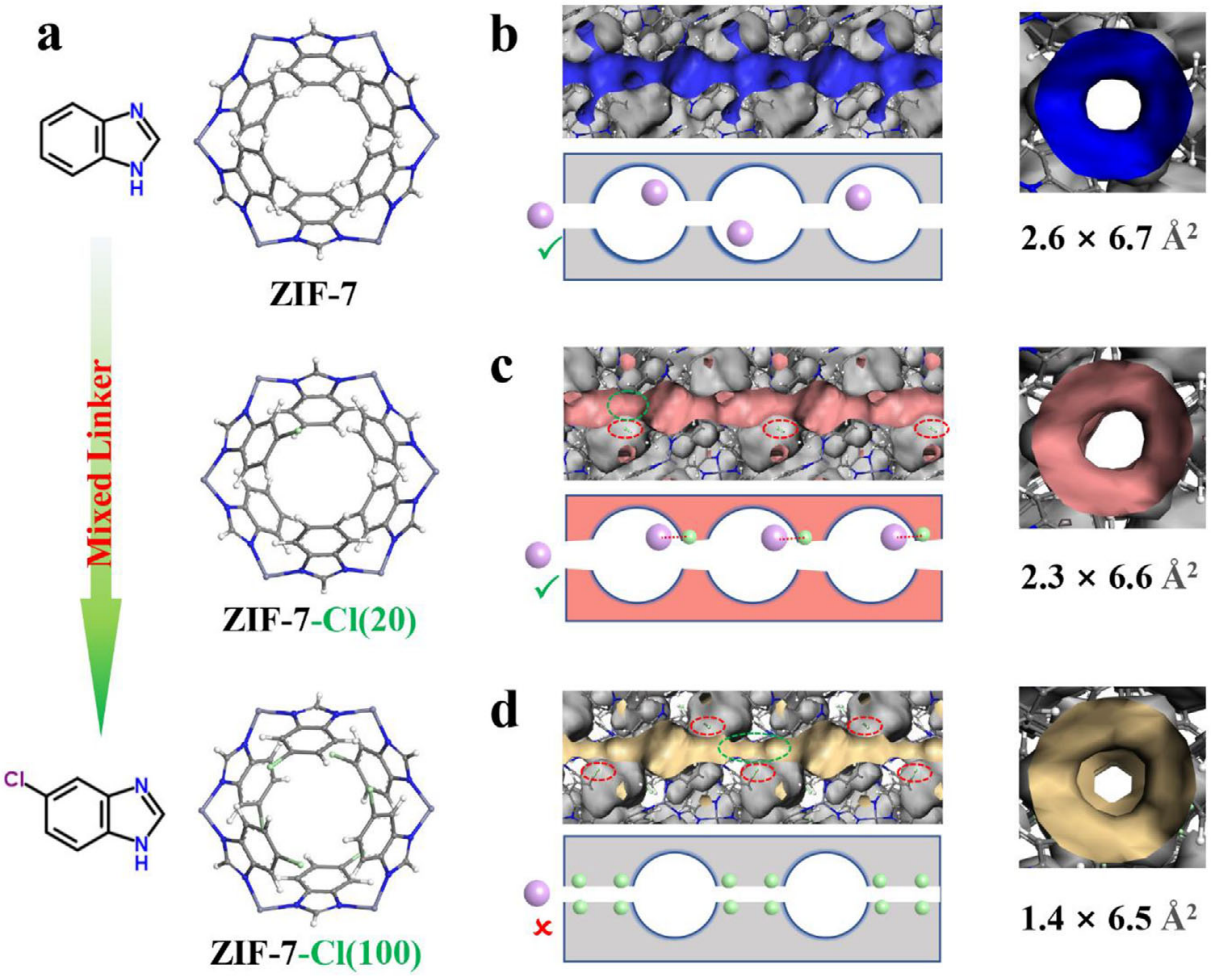
(a) Schematic diagram of introducing 5‐ClBzIM into the ZIF‐7 framework using a mixed linker strategy; Visualized pore channels running along the c‐axis, schematic diagrams of Xe adsorption, and dimensions of the largest pore and narrow window: (b) ZIF‐7; (c) ZIF‐7‐Cl(20) and (d) ZIF‐7‐Cl(100).

Adjusting the pore size and controlling the framework polarity are common strategies to improve the adsorption capacity of Xe [30]. In this regard, we propose a new strategy to partially adjust the pore size of the ZIF‐7 framework by using a mixed ligand strategy, and to prepare a novel mixed ligand adsorbent with sensitive recognition and high efficiency by locally strengthening the polarity of the ZIF‐7 framework with Cl atoms. To further verify our hypothesis, using the reported crystal structure of ZIF‐7 as a reference [31], structural models of ZIF‐7‐Cl(20) and ZIF‐7‐Cl(100) were constructed to simulate the effect of ligand substitution (Figure 1a). The introduction of chlorine functional groups induces precise structural modulation in the framework through the synergistic effects of ligand torsion and steric hindrance. In the prototypical ZIF‐7 structure, each benzimidazole ligand coordinates to the zinc nodes, forming a “candied hawthorn skewer”‐like channel with a maximum cavity diameter of 6.7 Å and a narrow window of 2.6 Å (Figure 1b). Upon partial ligand substitution with 5‐ClBzIM in ZIF‐7‐Cl(20), the combined electronic influence and steric demand of chlorine atoms trigger ligand twisting. This conformational adjustment propagates through the zinc nodes, leading to a slight framework contraction that reduces the cavity size to 6.6 Å. More significantly, the radial extension of chlorine atoms into the pore window creates a confinement effect, shrinking the aperture to 2.3 Å (Figure 1c). Such precise dimensional control preserves channel accessibility while introducing localized polarity, enabling discrimination of gas molecules based on polarizability. In the fully substituted ZIF‐7‐Cl(100), the ligand torsional effect is further amplified. The cooperative steric hindrance from multiple chlorine atoms within the window region causes a drastic reduction of the aperture to only 1.4 Å (Figure 1d). This excessive contraction not only restricts the framework's dynamic flexibility but also significantly impedes gas diffusion by elevating the transition‐state energy barrier—a mechanistic insight that explains the material's inefficacy in gas separation applications.

**FIGURE 2:**
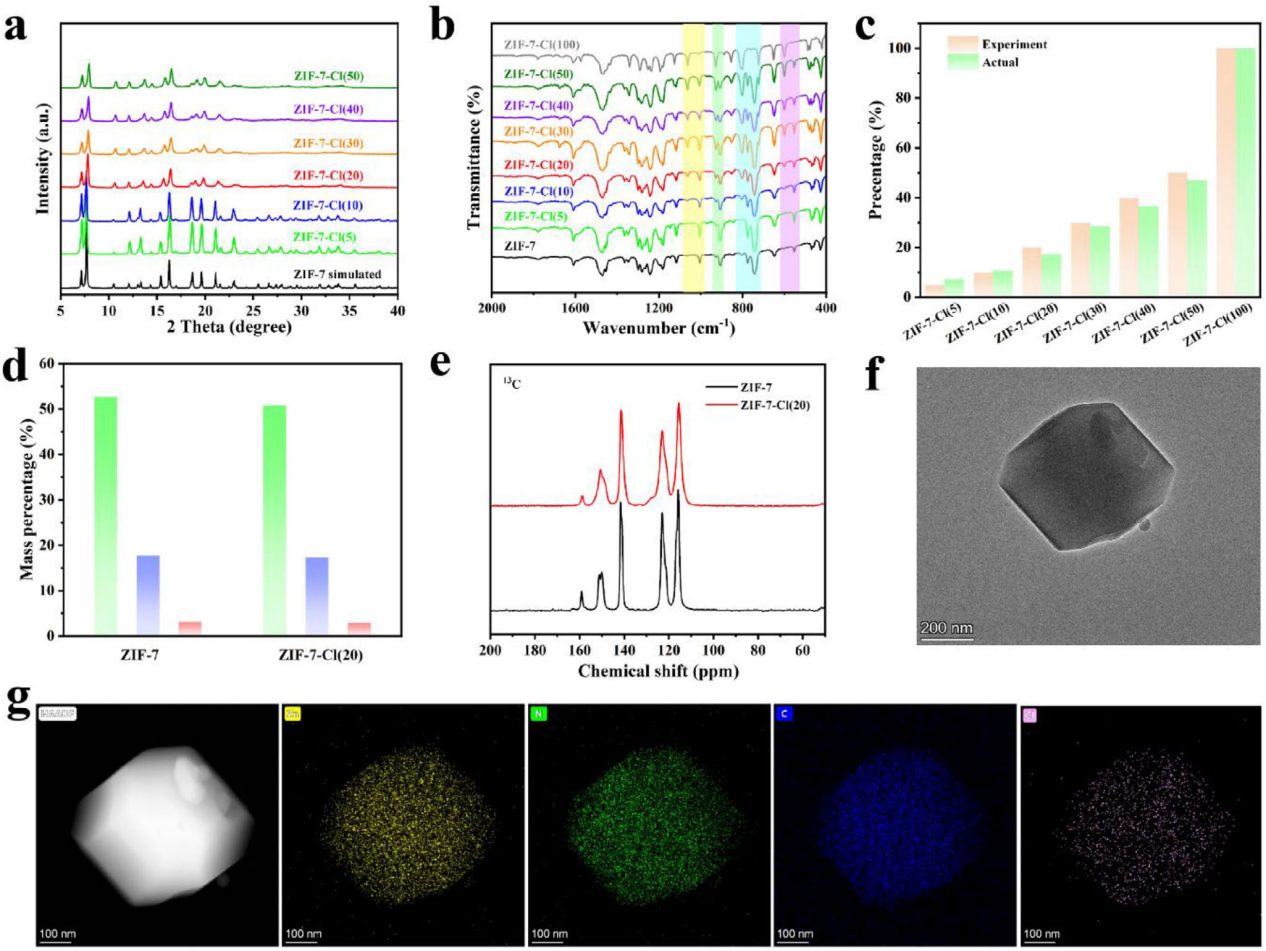
(a) PXRD pattern of ZIF‐7‐Cl(x); (b) FT‐IR spectra of ZIF‐7 and ZIF‐7‐Cl(x); (c) The percentage of 5‐ClBzIM added during the synthesis process and the actual 5‐ClBzIM content was obtained based on the ^1^H NMR results; (d) Elemental analysis results of ZIF‐7 and ZIF‐7‐Cl(20); (e) ^13^C SSNMR spectra of ZIF‐7 and ZIF‐7‐Cl(20); (f) TEM images and (g) TEM‐mapping element maps of ZIF‐7‐Cl(20).

To further verify our hypothesis, we synthesized ZIF‐7 and its derivative ZIF‐7‐Cl(x). To precisely engineer the pore environment of the archetypal flexible MOF, ZIF‐7, we employed a mixed‐ligand synthetic strategy. The parent ZIF‐7 was first synthesized [32], which, as we will demonstrate, exhibits suboptimal characteristics for Xe/Kr separation due to its high gate‐opening pressure. Subsequently, a series of isostructural ZIF‐7‐Cl(x) analogues were prepared by systematically introducing controlled molar ratios of a dual‐functionality co‐ligand, 5‐chlorobenzimidazole (5‐ClBzIM), in place of the native benzimidazole (BzIM) linker. This molecular engineering approach allowed us to probe the synergistic effects of pore size constriction and localized polarization on the overall separation performance. Powder X‐ray diffraction (PXRD) confirmed the successful synthesis of a series of ZIF‐7‐Cl(x) analogues that maintain the fundamental topology of ZIF‐7. The PXRD patterns of ZIF‐7‐Cl(x) materials match well with the simulated ZIF‐7 pattern, confirming phase purity and the feasibility of room‐temperature synthesis (Figure 2a). Notably, the characteristic diffraction peaks progressively broaden with increasing chlorine content, suggesting that the incorporation of larger chlorine atoms induces local lattice distortion and linker torsion. In contrast, the pattern of ZIF‐7‐Cl(100) shows significant deviation, confirming that excessive chlorine content leads to a twisted crystal framework (Figure S1). Fourier‐transform infrared (FT‐IR) spectroscopy provides supporting evidence for the successful incorporation of chlorine into the framework. The spectra of ZIF‐7‐Cl(x) evolve systematically toward that of ZIF‐7‐Cl(100) with higher chlorine loading (Figure 2b). Importantly, a gradually intensifying vibration band appears at 730 cm^−1^, attributed to C─Cl stretching, accompanied by a shift of the C─H stretching vibration from 900 to 930 cm^−1^ (Figure 2b and Figure S2). Nuclear magnetic resonance (^1^H NMR) spectroscopy further confirms the precise and controllable incorporation of the 5‐ClBzIM linker. The actual chlorine content in the synthesized materials agrees well with the feed ratio, yielding ZIF‐7‐Cl(5) (7.4%), ZIF‐7‐Cl(10) (10.7%), ZIF‐7‐Cl(20) (17.3%), ZIF‐7‐Cl(30) (28.5%), ZIF‐7‐Cl(40) (36.6%), and ZIF‐7‐Cl(50) (47%) (Figure 2c, Figure S3 and Table S1). These results demonstrate the versatility of this strategy for achieving precise chemical modification of ZIF‐7. Elemental analysis (EA) further validated the reliability of precisely modulating the ZIF‐7 framework. As shown in Figure 2d and Table S2, the mass percentages of C, N, and H in ZIF‐7 were 52.72%, 17.70%, and 3.13%, respectively, while those in ZIF‐7‐Cl(20) were 50.83%, 17.14%, and 2.91%, respectively. Based on the proportions of C, N, and H in BzIM and 5‐ClBzIM within the framework, it was found that 5‐ClBzIM accounted for 17.4% of the ZIF‐7‐Cl(20) framework (Figure S4), which is similar to the results of 1H NMR, strongly confirming the reliability of the precisely modulated mixed ligand strategy. Scanning electron microscopy (SEM) images reveal that all ZIF‐7‐Cl(x) materials exhibit a block‐like morphology similar to that of the parent ZIF‐7 (Figure S5), highlighting the robustness of the synthetic approach.

To further probe the spatial distribution of the chlorine‐functionalized linker within the framework, ^13^C Solid‐state nuclear magnetic resonance (SSNMR) and TEM‐EDS mapping analyses were performed on ZIF‐7‐Cl(20). The ^13^C SSNMR spectrum of pristine ZIF‐7 exhibits characteristic resonances in the 145–155 ppm region, which can be assigned to the imidazolate C═N carbons, while the signals between 110 and 140 ppm correspond to the aromatic carbons of the benzimidazolate linker [33]. Compared with pristine ZIF‐7, ZIF‐7‐Cl(20) retains the overall spectral profile, confirming preservation of the framework structure after mixed‐linker incorporation. Notably, the aromatic carbon resonances in the 120–140 ppm region become broader and slightly shifted upon Cl substitution (Figure 2e). This behavior can be attributed to the electron‐withdrawing effect of Cl atoms, which modifies the local electronic environments of neighboring aromatic carbons and generates a distribution of chemically inequivalent carbon sites [34, 35]. Importantly, no additional distinct resonance set corresponding to a separated Cl‐rich phase was observed. Instead, the continuous peak broadening suggests that the chlorine‐functionalized linkers are statistically dispersed within the framework rather than forming locally segregated domains. Furthermore, TEM‐EDS elemental mapping demonstrates that Cl is homogeneously distributed throughout the entire crystal and spatially overlaps well with Zn, N, and C signals, with no detectable Cl‐rich domains or phase separation (Figure 2g). These results are consistent with a homogeneous, non‐segregated distribution of chlorine‐functionalized linkers at the crystal scale, although atomic‐level randomness cannot be unambiguously resolved by these techniques.

**FIGURE 3:**
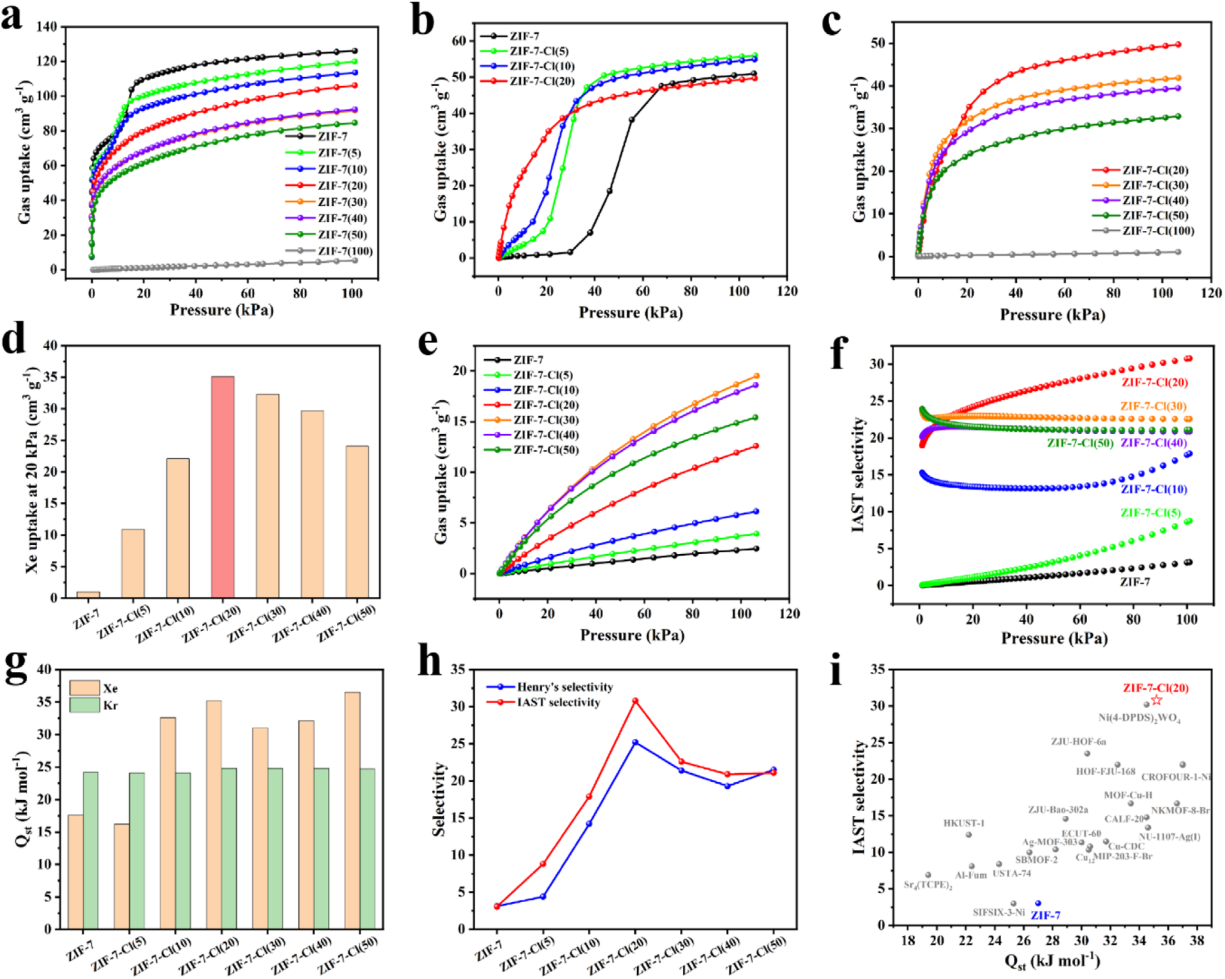
(a) CO_2_ adsorption isotherms of ZIF‐7 and ZIF‐7‐Cl(x) at 195 K; (b) and (c) Xe adsorption isotherms of ZIF‐7 and ZIF‐7‐Cl(x) at 298 K; (d) Xe capture at 20 kPa for ZIF‐7 and ZIF‐7‐Cl(x) at 298 K; (e) Kr adsorption isotherms of ZIF‐7 and ZIF‐7‐Cl(x) at 298 K; (f) IAST selectivity of ZIF‐7 and ZIF‐7‐Cl(x) for Xe/Kr (20/80, v/v) at 298 K; (g) Isostetic heats of adsorption (Q_st_) of ZIF‐7 and ZIF‐7‐Cl(x) for Xe and Kr at zero coverage; (h) Comparison of IAST selectivity and Henry's selectivity of ZIF‐7 and ZIF‐7‐Cl(x); (i) Comparison of IAST selectivity and Q_st_ of ZIF‐7‐Cl(20) with other advanced Xe/Kr separation adsorbents.

Thermogravimetric analysis (TGA) demonstrated excellent thermal stability for both ZIF‐7 and its chlorinated derivatives ZIF‐7‐Cl(x) (Figure S6). The mass loss observed below 150°C is attributed to the removal of residual solvents (methanol and DMF), while no framework decomposition occurred below 500°C for any of the materials. Subsequent N_2_ adsorption‐desorption isotherms measured at 77 K revealed the limitations of using N_2_ as a probe molecule for porosity evaluation in these flexible frameworks. ZIF‐7 exhibits negligible N_2_ uptake, yielding an apparent BET surface area of only 38 m^2^ g^−1^, significantly lower than the theoretical value (Figure S7) [36]. Similar N_2_ “closed‐pore” behavior was observed across the ZIF‐7‐Cl(x) series, with BET surface areas remaining below 20 m^2^ g^−1^ (Table S3). To accurately characterize the porous properties, CO_2_ adsorption isotherms were collected at 195 K. As shown in Figure 3a, ZIF‐7 displays a characteristic two‐step adsorption profile, featuring initial uptake at low pressures followed by a distinct gate‐opening transition that significantly enhances CO_2_ capture capacity. Low‐chlorine variants ZIF‐7‐Cl(5) and ZIF‐7‐Cl(10) maintain similar flexible adsorption characteristics, though with progressively reduced gate‐opening pressures. With increasing chlorine content, the materials transition from flexible to rigid‐like adsorption behavior, likely due to modified coordination geometry at Zn nodes that restricts framework flexibility. Concurrently, the steric effects of chlorine atoms systematically reduce pore aperture size and volume, leading to progressively lower CO_2_ saturation capacities. In the extreme case, ZIF‐7‐Cl(100) shows negligible CO_2_ adsorption (5 cm^3^ g^−1^) due to severely narrowed pore windows and substantial suppression of framework flexibility. BET surface areas calculated from the 195 K CO_2_ adsorption data further corroborate this structural evolution: ZIF‐7 (339 m^2^ g^−1^) > ZIF‐7‐Cl(5) (300 m^2^ g^−1^) > ZIF‐7‐Cl(10) (275 m^2^ g^−1^) > ZIF‐7‐Cl(20) (236 m^2^ g^−1^) > ZIF‐7‐Cl(30) (200 m^2^ g^−1^) ≈ ZIF‐7‐Cl(40) (203 m^2^ g^−1^) > ZIF‐7‐Cl(50) (183 m^2^ g^−1^) > ZIF‐7‐Cl(100) (8 m^2^ g^−1^) (Table S3). Pore size distribution (PSD) analysis similarly confirms progressive pore narrowing (Figure S8). PLATON calculations reveal a decreasing trend in accessible solvent volume: ZIF‐7 (26.6%) > ZIF‐7‐Cl(20) (24.6%) > ZIF‐7‐Cl(100) (18.5%), clearly demonstrating the pore‐constricting effect of chlorine substitution. Furthermore, water vapor adsorption tests show comparable uptake for ZIF‐7 (31 mg g^−1^) and ZIF‐7‐Cl(20) (34 mg g^−1^) (Figure S9), indicating that chlorine incorporation does not significantly alter material hydrophobicity, with both materials maintaining effective resistance to moisture under humid conditions.

To investigate the Xe adsorption properties and Xe/Kr separation potential of ZIF‐7 and its chlorinated derivatives ZIF‐7‐Cl(x), we measured single‐component adsorption isotherms for Xe and Kr at 273 and 298 K. At 273 K, ZIF‐7 exhibits typical flexible adsorption behavior, characterized by a distinct gate‐opening pressure (P_go_) (Figure S10). With increasing chlorine content in the framework, this P_go_ gradually decreases, and the adsorption behavior progressively suppresses framework flexibility. At 298 K, ZIF‐7 shows a high P_go_, which is unfavorable for efficient separation at 20 kPa. However, the introduction of small amounts of chlorine significantly lowers the P_go_, with values decreasing to 18.5 kPa for ZIF‐7‐Cl(5) and 14.3 kPa for ZIF‐7‐Cl(10) (Figure 3b). Despite this improvement, their Xe uptake at 20 kPa remains limited to 10.9 and 22.1 cm^3^ g^−1^, respectively (Figure 3d), substantially lower than their saturation capacities at 1 bar, thus restricting separation efficiency. Notably, when the molar fraction of 5‐ClBzIM reaches 20%, ZIF‐7‐Cl(20) begins to display nearly rigid‐like adsorption behavior while maintaining a high Xe saturation capacity of 49.6 cm^3^ g^−1^, comparable to ZIF‐7. As the secondary linker proportion increases further, the enhanced framework polarity improves low‐pressure Xe affinity, but the steric effects of chlorine atoms narrow the pore size, resulting in a systematic decrease in Xe capacity to 41.8 cm^3^ g^−1^ for ZIF‐7‐Cl(30), 39.4 cm^3^ g^−1^ for ZIF‐7‐Cl(40), 32.8 cm^3^ g^−1^ for ZIF‐7‐Cl(50), and merely 1 cm^3^ g^−1^ for ZIF‐7‐Cl(100) (Figure 3c). Crucially, at the separation‐relevant pressure of 20 kPa, ZIF‐7‐Cl(20) achieves an exceptional Xe uptake of 35.1 cm^3^ g^−1^, outperforming all other samples. This result indicates an optimal balance between adsorption behavior (flexibility/rigidity) and capacity achieved through secondary linker incorporation – insufficient substitution fails to adequately suppress framework flexibility, while excessive substitution severely compromises adsorption capacity. To gain deeper insights into the transient transport mechanisms and structural responses of the flexible frameworks during the adsorption process, time‐resolved Xe adsorption kinetic measurements were performed for both ZIF‐7 and ZIF‐7‐Cl(20) at 298 K under 20 and 100 kPa, respectively (Figure S11). As illustrated in Figure S11a, under a lower Xe pressure of 20 kPa, pristine ZIF‐7 exhibits an extremely low Xe adsorption capacity (5.1 cm^3^ g^−1^) and instantly plateaus within 1 min, demonstrating that its pore windows remain completely locked in the narrow‐pore phase. Notably, ZIF‐7‐Cl(20) displays an S‐shaped kinetic curve featuring three distinct regimes, a minor initial induction phase (<2 min), a rapid cooperative gate‐opening acceleration phase (2–15 min), and a subsequent diffusion‐controlled saturation plateau. The transient curve provides conclusive kinetic evidence that the local polarity and pore engineering achieved through chlorine functionalization successfully improve kinetics and low‐pressure pore accessibility, enabling the structure to undergo intelligent gated opening transitions even under low xenon pressures. When the pressure elevated to 100 kPa (Figure S11b), the enormous thermodynamic potential drives both samples to open their gates aggressively. Consequently, both ZIF‐7 and ZIF‐7‐Cl(20) show ultra‐fast uptake rates, with the pore channels being almost filled within the initial several minutes, reaching their thermodynamic equilibrium capacities (52 and 48 cm^3^ g^−1^). Kr exhibits kinetic adsorption behavior similar to that of rigid materials, characterized by rapid adsorption and equilibrium (Figure S11c). This comprehensive kinetic analysis firmly establishes that the taming of framework flexibility in ZIF‐7‐Cl(20) works as a dual thermodynamic‐kinetic, fundamentally underpinning its exceptional dynamic Xe/Kr breakthrough separation efficiency under practical operating conditions. Furthermore, frameworks doped with other halogens, such as F and Br also exhibits rigid CO_2_ and Xe adsorption behavior (Figures S13, S14). However, while the smaller F atoms maintain a high Xe trapping capacity, their lower polarity results in a slightly lower Xe recognition ability compared to Cl in the low‐pressure region. Furthermore, due to the larger atomic radius of Br, the adsorption capacity of Xe is significantly reduced, which is not conducive to Xe/Kr separation. In contrast, all adsorbents exhibit near‐rigid adsorption characteristics toward Kr (Figure 3e and Figure S12). Kr uptake initially increases with chlorine introduction, reaching a maximum of 19.5 cm^3^ g^−1^ for ZIF‐7‐Cl(30), before declining due to excessive pore narrowing. ZIF‐7‐Cl(20) combines a rigid‐like Xe adsorption profile, exceptional Xe working capacity at 20 kPa, and low Kr uptake, making it a highly promising adsorbent for efficient Xe extraction from Xe/Kr (20/80, v/v) mixtures.

The separation potential of the materials for Xe/Kr (20/80, v/v) mixtures was evaluated using the Ideal Adsorbed Solution Theory (IAST). The single‐component adsorption isotherms at 273 and 298 K were fitted with single‐site and dual‐site Langmuir‐Freundlich models to calculate the IAST selectivity (Figures S15–S42). At 273 K and 1 bar, ZIF‐7‐Cl(20) exhibits a remarkably high IAST selectivity of 54.5, significantly outperforming the ZIF‐7 (12.5) and other ZIF‐7‐Cl(x) materials (Figure S43). This superior performance is maintained at 298 K, where ZIF‐7‐Cl(20) achieves a selectivity of 30.8, in stark contrast to ZIF‐7 (Figure 3f). These results unequivocally demonstrate that incorporating an optimal amount of the secondary linker is an effective strategy for enhancing separation selectivity. The IAST selectivity as a function of chlorine content reveals a distinct volcano‐shaped trend (Figure 3h). The selectivity increases with the 5‐ClBzIM fraction up to 20% (ZIF‐7‐Cl(5): 8.8; ZIF‐7‐Cl(10): 17.9), peaks at this optimal composition, and subsequently decreases with further chlorine incorporation (ZIF‐7‐Cl(30): 22.6; ZIF‐7‐Cl(40): 20.9; ZIF‐7‐Cl(50): 21.1). This optimal performance is attributed to a balanced trade‐off: moderate chlorine functionalization enhances framework polarity and thus Xe affinity, without excessively compromising adsorption capacity due to steric effects, achieving an optimal balance between polarity and pore architecture. Benchmarking against other advanced adsorbents further highlights the competitiveness of ZIF‐7‐Cl(20). Its IAST selectivity (30.8) is comparable to the high‐performing Ni(4‐DPDS)_2_WO_4_ (30.2) [37] and substantially surpasses many reported materials, including HOF‐FJU‐168 (22) [38], MOF‐Cu‐H (16.7) [39], NKMOF‐8‐Br (16.7) [40], MIP‐203‐F‐Br (10.8) [41], ECUT‐60 (11.4) [42] and SIFSIX‐3‐Ni (3) [43] (Figure 3i). Furthermore, Henry's selectivity was calculated by fitting the adsorption isotherms in the very low‐pressure region to assess the separation capability at trace concentrations (Figures S44–S57). ZIF‐7‐Cl(20) shows an outstanding Henry's selectivity of 25.2, following the same trend observed for IAST selectivity (Figure 3h), and exceeds the performance of benchmarks such as Zr‐Fum‐Me (14.8) [44] and UiO‐66‐NH_2_Me)_2_ (14.4) [45]. This consistency across different pressure regimes confirms the exceptional Xe/Kr separation potential of ZIF‐7‐Cl(20) over a wide range of operating conditions.

The adsorption isotherms for Xe and Kr measured at 273 and 298 K were analyzed using the virial method to determine the isosteric heats of adsorption (Q_st_) at zero coverage (Figures S58–S71). As shown in Figure 3g and Figure S72, ZIF‐7‐Cl(20) exhibits a notably high initial Q_st_ for Xe of 35.2 kJ mol^−1^, significantly surpassing that of pristine ZIF‐7 (27 kJ mol^−1^). The binding strength of ZIF‐7‐Cl(20) for Xe ranks among the highest reported for advanced adsorbents. Its Q_st_ is lower than CROFOUR‐1‐Ni (37 kJ mol^−1^) [46] and substantially exceeds those of prominent materials including Sr_4_(TCPE)_2_ (19.4 kJ mol^−1^) [47], USTA‐74 (24.3 kJ mol^−1^) [48], SBMOF‐2 (26.4 kJ mol^−1^) [49], Cu_12_ (30.5 kJ mol^−1^) [50], and Cu‐CDC (31.7 kJ mol^−1^) [51] (Figure 3i). In contrast, the Q_st_ for Kr adsorption across the ZIF‐7‐Cl(x) series remains consistently low (24.1–24.8 kJ mol^−1^, Figure S73). This pronounced differential in adsorption enthalpy between Xe and Kr unambiguously demonstrates the higher affinity of ZIF‐7‐Cl(20) for Xe, providing a fundamental thermodynamic basis for its exceptional potential in selective separation of Xe/Kr mixtures.

**FIGURE 4:**
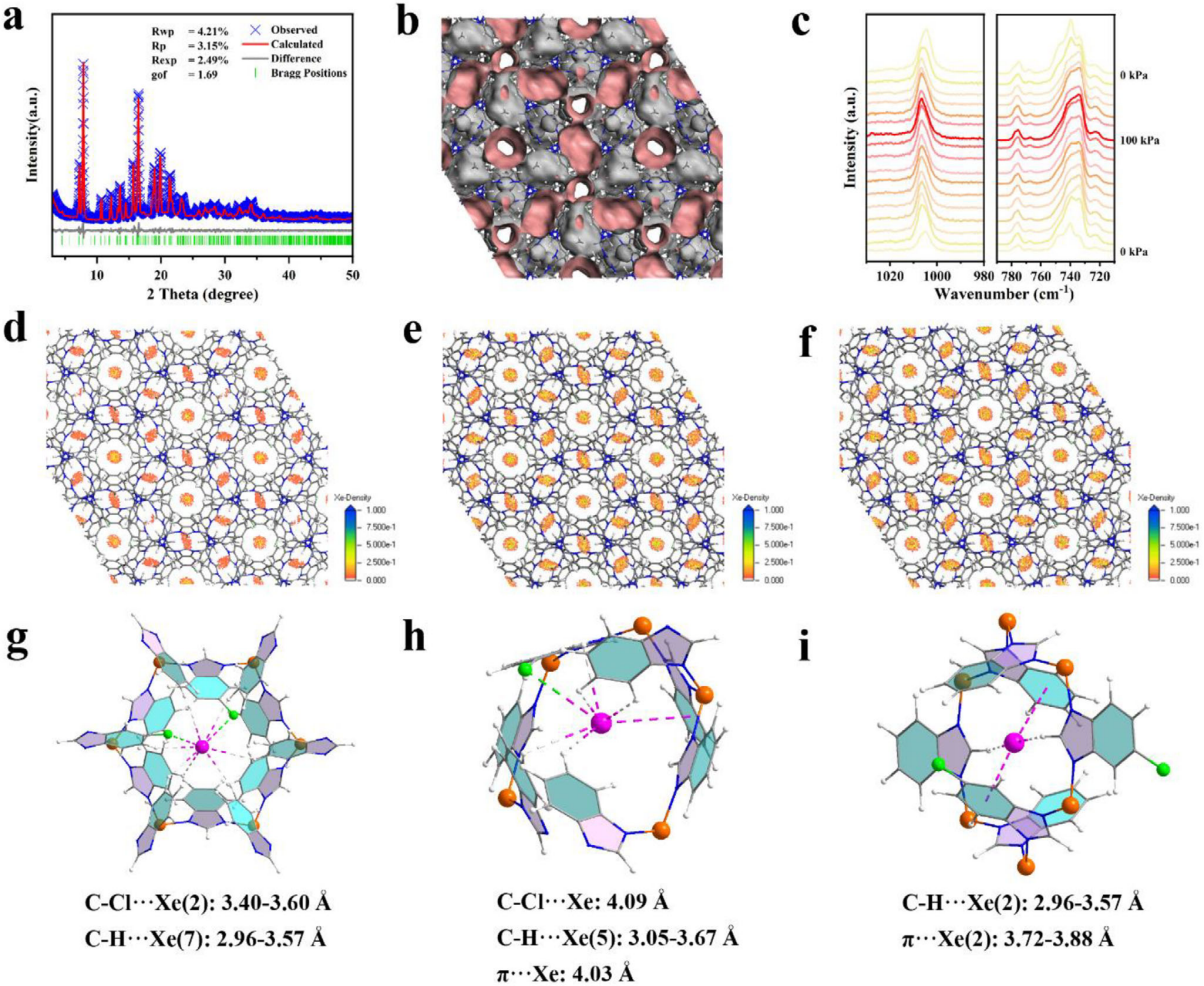
(a) Rietveld refinement of ZIF‐7‐Cl(20); (b) Visualized channels along the c‐axis of the refined ZIF‐7‐Cl(20) framework; (c) In situ FT‐IR spectra of ZIF‐7‐Cl(20) loaded with Xe under different pressures; GCMC simulations of xenon loading density at different pressures at 298 K: (d) 1 kPa; (e) 20 kPa; (f) 100 kPa; Xe binding sites in the ZIF‐7‐Cl(20) framework: (g) Site I, the pores distributed along the c‐axis through the channel; (h) Site II, the pores connected to the c‐channel constructed with the participation of 5‐ClBzIM; (i) Site III, another pore cavity connected to the c channel (Color codes: Zn, orange; Cl, green; Xe, purplish‐red; C, grey; N, blue; H, white).

To elucidate the Xe adsorption mechanism within the ZIF‐7‐Cl(20) framework, the PXRD patterns were refined using Topas v5.0 with Rietveld to construct an average structural model consistent with CI incorporation (CCDC number: 2529844, Table S4). The refinement results show excellent agreement with experimental data (Figure 4a), confirming that Cl atoms are strategically distributed within the pore cavities, consistent with prior structural simulations. This Cl‐functionalization significantly modulates the pore architecture, the framework features narrow windows (2.3 × 2.6 Å^2^) and larger cavities (6.5 × 6.6 Å^2^) along the c‐axis, while the interconnected channels exhibit dimensions of 2.9 × 4.3 Å^2^ (Figure 4b). These features underscore the pronounced steric and pore‐modifying effects induced by the chlorine substituents (Figures S74 and S75). Furthermore, in situ FT‐IR spectroscopy under varying Xe pressures was employed to investigate the adsorption mechanism (Figure S76 and Figure 4c). At 10 kPa, the characteristic C─Cl stretching vibration at 739 cm^−1^ shifted to 734 cm^−1^ with a concomitant increase in intensity, providing supporting evidence of a polar interaction between the C─Cl bond of 5‐ClBzIM and the Xe guests. Furthermore, the shifts of the peaks at 1004 and 1117 cm^−1^ to 1006 and 1119 cm^−1^ respectively, are attributed to weak interactions between Xe and the C─H bonds or the aromatic π‐systems of the benzimidazole ligands. These shifts were evident starting from 10 kPa and persisted up to 100 kPa. Notably, the shifts remained pronounced even as the pressure was reduced back to 10 kPa, demonstrating the near‐rigid adsorption behavior of ZIF‐7‐Cl(20) and its capability for sensitive Xe capture at low pressures.

Grand Canonical Monte Carlo (GCMC) simulations were further conducted to study the Xe adsorption behavior in ZIF‐7‐Cl(20). The adsorption density distributions under different Xe pressures (1, 20, and 100 kPa) at 298 K were simulated (Figure 4d–f). The results reveal significant Xe density within the framework even at 1 kPa, confirming its excellent low‐pressure capture capability. At 1 kPa, the density profile clearly identifies three distinct adsorption sites, with site III showing relatively low occupancy (Figure S77), underscoring the important role of polar Cl atoms in Xe recognition. As the pressure increases to 20 and 100 kPa, the adsorption density further rises, with distributions at these two pressures being similar—consistent with the low‐pressure saturation observed in the experimental adsorption isotherm. This indicates that near‐saturation Xe adsorption can be achieved at low partial pressures. Further site analysis reveals a unique adsorption mechanism. Xe molecules are primarily located in two types of regions: the main channels extending along the c‐axis, lined by aromatic rings of six benzimidazole ligands, and the interconnected secondary ring‐shaped cavities arranged in a staggered bilayer (Figure S77). At adsorption site I (Figure 4g), a strong C─Cl···Xe polar interaction is observed with distances of 3.40–3.60 Å, significantly shorter than the sum of the van der Waals radii (3.91 Å), indicating strong binding. Notably, the introduction of 5‐ClBzIM creates a heterogeneous chemical environment in the secondary cavities. At site II (Figure 4h), the replacement of H by Cl enhances framework polarity, leading to a polar Cl···Xe interaction (4.09 Å). This interaction causes a displacement of Xe within the cavity, thereby strengthening weak C─H···Xe interactions (3.05–3.67 Å) with adjacent aromatic rings. The synergy of multiple interactions enhances the overall binding affinity. Moreover, regions of unsubstituted BzIM remain in the framework (site III, Figure 4i), where the pore environment resembles that of pristine ZIF‐7. Here, Xe exhibits weaker C─H···Xe (2.96–3.57 Å) and π···Xe (4.00–4.59 Å) interactions. In summary, by precisely tuning the 5‐ClBzIM incorporation ratio, we have successfully preserved the effective diffusion pathways while creating a high density of Cl‐based polar sites. The synergy between steric confinement and enhanced framework‐guest interactions provides a clear microscopic rationale for the high Xe capacity and affinity of ZIF‐7‐Cl(20). This rational linker‐mixing strategy offers a powerful template for the design of high‐performance adsorbents for noble gas separation.

**FIGURE 5:**
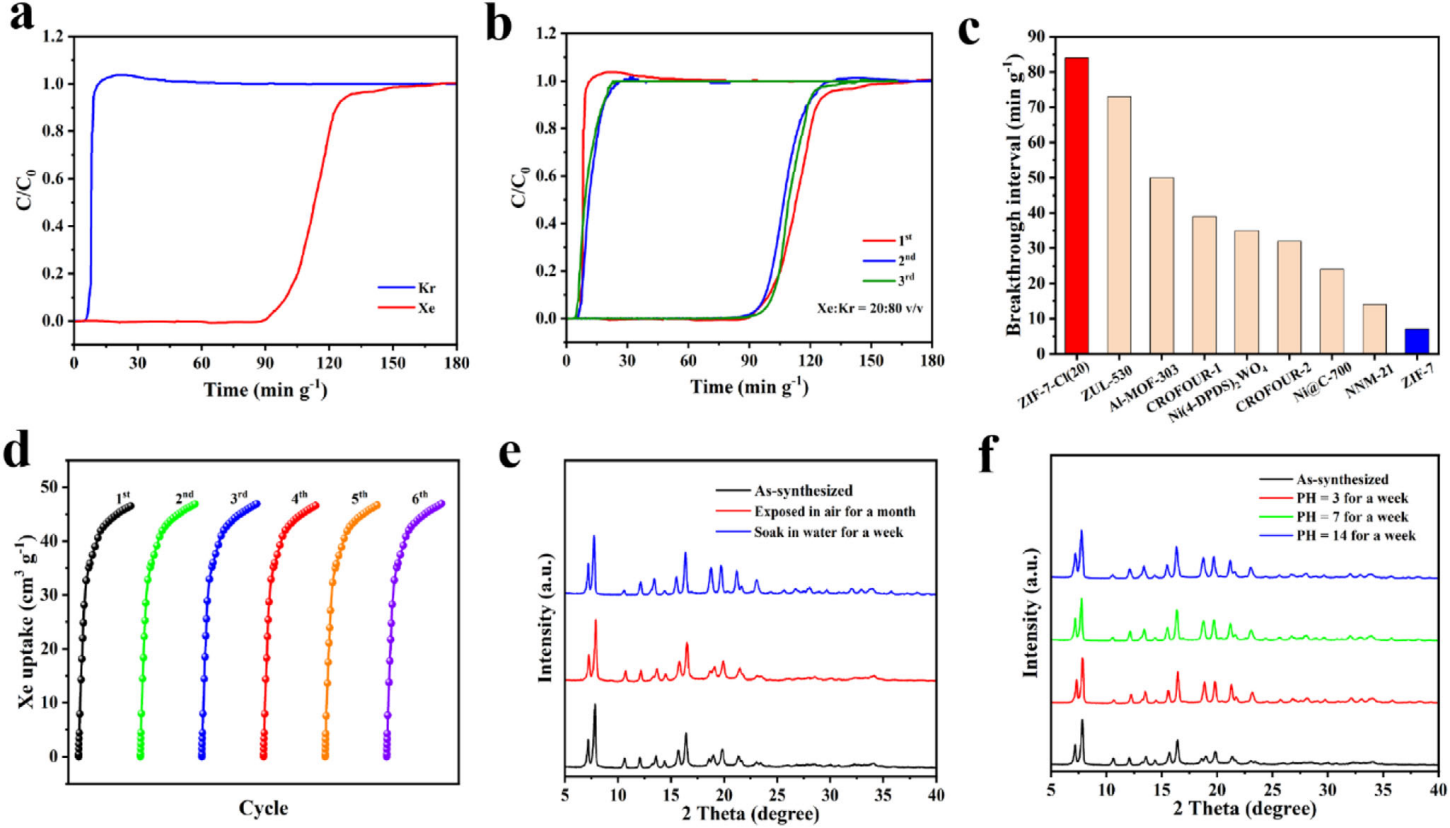
(a) Dynamic column curves of Xe/Kr (20:80, v/v) in ZIF‐7‐Cl(20) at a flow rate of 2 mL min^−1^ at 298 K. (b) Three‐cycle dynamic breakthrough curves at a flow rate of 2 mL min^−1^ at 298 K; (c) Comparison of the Xe/Kr breakthrough intervals of ZIF‐7‐Cl(20) with other reported Xe/Kr separation materials. (d) Six‐cycle Xe adsorption curves of ZIF‐7‐Cl(20) at 298 K; (e) PXRD patterns of ZIF‐7‐Cl(20) in water and after exposure to air; (f) PXRD patterns of ZIF‐7‐Cl(20) after treatment in acidic and alkaline environments.

Inspired by the rigid‐like Xe adsorption behavior and exceptional Xe/Kr selectivity of ZIF‐7‐Cl(20), we further conducted dynamic column breakthrough experiments to evaluate its practical separation performance for a Xe/Kr (20:80, v/v) binary mixture. At 298 K, the gas mixture was introduced at a flow rate of 2 mL min^−1^ into a fixed‐bed column packed with approximately 1 g of adsorbent. The results show that both ZIF‐7 and ZIF‐7‐Cl(20) enable Xe/Kr separation (Figure 5a and Figure S78). However, due to its higher gate‐opening pressure for Xe, ZIF‐7 exhibits limited dynamic separation performance. Kr breaks through at 1.7 min g^−1^, followed by Xe elution at 11.3 min g^−1^, resulting in a narrow breakthrough interval of only 9.6 min g^−1^ (Figure S79). In contrast, ZIF‐7‐Cl(20) demonstrates outstanding dynamic separation, Kr breaks through at 5.3 min g^−^^1^, while Xe is retained until 89.8 min g^−1^, yielding an extensive breakthrough interval of 84.5 min g^−1^ (Figure 5a). This not only significantly surpasses that of ZIF‐7, but also exceeds those of most reported adsorbents, such as Ni(4‐DPDS)_2_WO_4_ (35 min g^−1^) [37] and ZUL‐530 (73 min g^−1^) [52] (Figure 5c). The dynamic Xe uptake of ZIF‐7‐Cl(20), calculated from the breakthrough curve, reaches 45.4 cm^3^ g^−1^, which is comparable to its single‐component adsorption capacity and substantially higher than that of ZIF‐7 (15.4 cm^3^ g^−1^) (Figures S80 and S81). These results confirm that chlorine functionalization not only optimizes low‐pressure Xe recognition but also leads to superior adsorption separation under dynamic mixed‐gas conditions. Furthermore, ZIF‐7‐Cl(20) exhibits a Xe/Kr separation factor of 4, and the selectivity calculated from the breakthrough curve (until Xe reaches feed concentration) reaches 16, which is significantly higher than that of ZIF‐7 (9). In addition, ZIF‐7‐Cl(20) achieves a high‐purity Kr (>99.9%) productivity of 129 L kg^−1^, outperforming benchmark materials such as HOF‐40 (44.4 L kg^−1^) [53] and ECUT‐60 (96 L kg^−1^) [42]. Over three consecutive adsorption–desorption cycles, no obvious degradation in breakthrough time or adsorption capacity was observed, indicating excellent cyclic stability and regenerability (Figure 5b). With high dynamic capacity, high selectivity, and outstanding recyclability, ZIF‐7‐Cl(20) demonstrates exceptional performance in Xe/Kr separation. Compared to energy‐intensive cryogenic distillation, this adsorption‐based strategy offers a highly promising alternative for efficient Xe/Kr separation.

Given the demanding conditions of practical industrial separation, we systematically evaluated the structural stability and applicability of ZIF‐7‐Cl(20) from multiple perspectives. First, the material retained its original PXRD patterns after one month of exposure to air and one week of immersion in water, confirming its structural integrity and preserved crystallinity (Figure 5e). Water vapor adsorption tests further verified that ZIF‐7‐Cl(20) maintains hydrophobicity similar to that of pristine ZIF‐7 (Figure S9), indicating good moisture tolerance. Chemically, ZIF‐7‐Cl(20) retained its structural integrity after being soaked in aqueous solutions with pH values ranging from 3 to 14 for one week (Figure 5f). Moreover, treatment in various organic solvents, including DMF, methanol, ethanol, dichloromethane, acetonitrile, and acetone for one week, resulted in PXRD patterns identical to the as‐synthesized sample, with no signs of crystallinity degradation (Figure S83), highlighting its exceptional chemical stability in diverse environments. To evaluate its recyclability, six consecutive Xe adsorption–desorption cycles were performed at 298 K, during which regeneration could be readily achieved by simple desorption at 60°C for 30 min. The results demonstrate that ZIF‐7‐Cl(20) maintained highly consistent adsorption behavior and Xe uptake capacity throughout the repeated cycles (Figure 5d), in agreement with the cycling stability observed in the dynamic breakthrough experiments. Over the six cycles, ZIF‐7‐Cl(20) exhibited an average Xe capacity of 46.6 cm^3^ g^−1^ with only a 0.6% fluctuation. Importantly, the adsorption–desorption isotherms nearly overlapped across all six cycles (Figure S84), indicating that the adsorption behavior remained unchanged during repeated operation and confirming the excellent cyclic stability of ZIF‐7‐Cl(20). These findings demonstrate that ZIF‐7‐Cl(20) not only exhibits outstanding Xe/Kr separation performance but also possesses the structural robustness and regeneration capability essential for long‐term operation under complex chemical conditions. Combined with its mild room‐temperature synthesis and low‐cost raw materials, this adsorbent shows significant application potential and competitiveness in industrial gas separation, particularly for energy‐efficient Xe/Kr separation.

## 3 Conclusion

Through a mixed‐ligand strategy under ambient conditions, we developed a series of isostructural ZIF‐7 adsorbents functionalized with precisely controlled chlorine‐substituted co‐ligands. The unique pore configuration of ZIF‐7‐Cl(20), characterized by inwardly convex cavities and localized polarization induced by Cl atoms, enables sensitive Xe recognition and triggers a framework that substantially suppresses framework flexibility and induces near‐rigid Xe adsorption characteristics. This transformation effectively overcomes the low‐pressure uptake limitation typical of flexible adsorbents. Combining high Xe capacity with enhanced low‐pressure affinity, ZIF‐7‐Cl(20) demonstrates exceptional Xe/Kr (20/80, v/v) separation performance, exhibiting an IAST selectivity of 30.8 at 298 K and 1 bar. Dynamic breakthrough measurements further confirm its superior separation capability, showing a remarkable Xe/Kr breakthrough interval of 84 min g^−1^ and producing high‐purity Kr (>99.9%) with a productivity of 129 L kg^−1^. These metrics position ZIF‐7‐Cl(20) among the top‐performing Xe/Kr separators reported to date. Grand canonical Monte Carlo simulations reveal that strong Xe···Cl interactions serve as the fundamental mechanism for the observed sensitive Xe capture. Excellent regenerability and framework robustness underscore its potential for practical implementation. This work not only provides strategic insights into the rational design of flexible porous materials through mixed‐ligand functionalization but also represents a significant advancement in developing efficient separation technologies for noble gases.

## 4 Experimental Section

### 4.1 Synthesis of ZIF‐7

The synthesis of ZIF‐7 is consistent with that previously reported [32]. Zn(OAc)_2_·2H_2_O (3.7 mmol, 0.81 g) was first dissolved in a mixed solvent (20 mL, DMF: ethanol: DI water: ammonium hydroxide (30%) = 5:5:7.5:2.5, v/v). BIM (10.8 mmol, 1.27 g) was slowly added to the zinc solution while stirring. The reaction mixture was then stirred at 150 rpm at room temperature for 4 days. After centrifugation, the resulting white powder was rinsed with DMF (20 mL) and then washed with methanol (3 × 20 mL). The washed white powder was dried in a vacuum oven at 60°C overnight.

### 4.2 Synthesis of ZIF‐7‐Cl(x)

The synthesis of ZIF‐7‐Cl(x) is similar to ZIF‐7. During the synthesis process, different proportions of 5‐Cl‐BzIM were added to replace BzIM to synthesize ZIF‐7‐Cl(x) (x = 5, 10, 20, 30, 40, 50, and 100), where x represents the proportion of 5‐Cl‐BzIM. As the 5‐Cl‐BzIM content increases, the obtained powder product gradually changes from white to brown. Before evaluating the gas adsorption performance, all adsorbents underwent a solvent exchange process, where they were soaked in methanol for 3 days and replaced with new methanol every 12 h. Subsequently, the material was dried under vacuum at 423 K for 12 h to prepare an activated material suitable for measurement.

### 4.3 Materials Characterization

Powder X‐ray diffraction (PXRD) patterns were recorded by a Bruker D8 Advance diffractometer using Cu Ka radiation operating at 40 kV, 44 mA, scanning in the range of 2θ from 3° to 40° at a rate of 8.0° min^−1^. Thermogravimetric analysis (TGA) was performed using a Pyris Diamond TGA under N_2_ atmosphere from 303 to 1073 K at a heating rate of 10°C min^−1^. Liquid nuclear magnetic resonance (NMR) spectroscopy was performed using a Bruker Avance III at 400 MHz to calibrate the H spectrum (^1^H‐NMR) to determine the content of the mixed ligands. The deuterated reagent was acetic acid‐d_4_. Fourier transform infrared (FTIR) spectra were recorded in the wavenumber range of 4000–400 cm^−1^ using an IS50 FTIR spectrometer from Thermo Fisher Scientific (formerly Thermo Electron). High‐resolution solid‐state nuclear magnetic resonance (SSNMR) spectra were recorded on a Bruker 400 MHz spectrometer using a 4 mm MAS rotor. The measurements were performed at a magic‐angle spinning (MAS) rate of 10 kHz with a recycle delay of 4 s. A cross‐polarization (CP) pulse sequence was employed for spectral acquisition, and the pre‐scan delay was set to 6.5 µs. Transmission electron microscopy (TEM) measurements were carried out on an FEI Talos F200S transmission electron microscope. The adsorption isotherm was collected by an automatic adsorption instrument (BSD‐660 M). About 150 mg of the sample was first activated in a vacuum at 423 K, and then reactivated in a vacuum environment at 333 K for each repeated test. The BET specific surface area and permanent pores were obtained by analyzing the N_2_ adsorption‐desorption isotherm at 77 K in liquid nitrogen and the CO_2_ adsorption‐desorption isotherm in a mixed bath of dry ice and acetone at 195 K, respectively.

Deposition number CCDC 2529844 (ZIF‐7‐Cl(20)) contains the supplementary crystallographic data for this paper. These data can be obtained free of charge from The Cambridge Crystallographic Data Centre via www.ccdc.cam.ac.uk/data_request/cif.

## Conflicts of Interest

The authors declare no conflicts of interest.

## Supporting information




**Supporting File 1**: advs76155‐sup‐0001‐SuppMat.docx.

**Supporting File 2**: advs76155‐sup‐0002‐SuppMat.cif.

## Data Availability

The data that support the findings of this study are available from the corresponding author upon reasonable request.
