# Supplementary material for: Taming Flexibility: Synergistic Pore and Polarity Engineering in a MOF for High‐Efficiency Xe/Kr Separation
Source: Adv Sci (Weinh). 2026 Jun 15:e76155. Online ahead of print. doi: 10.1002/advs.76155 (PMC13336414; doi:10.1002/advs.76155)
Supplement: Supplementary file 1 — Supporting File 1: advs76155‐sup‐0001‐SuppMat.docx. [file ADVS-9999-e76155-s002.docx]

Supporting Information

Taming Flexibility: Synergistic Pore and Polarity Engineering in a MOF for High-Efficiency Xe/Kr Separation

Tao Zhao, Xue Wang, Youjin Gong, Siqi Dong, Shunshun Xiong,* Hui Xu, Aziz Bakhtiyarovich Ibragimov, Thamraa Alshahrani, He Zheng, Lin Li, Junkuo Gao*

T. Zhao, X. Wang, Prof. J. Gao

China-Uzbekistan Joint Laboratory on Advanced Porous Materials, State Key Laboratory of Bio-based Fiber Materials, School of Materials Science and Engineering, Zhejiang Sci-Tech University, Hangzhou 310018 (China)

E-mail: jkgao@zstu.edu.cn

Y. Gong, S. Dong, Prof. S. Xiong

Institute of Nuclear Physics and Chemistry, China Academy of Engineering Physics, Mianyang, Sichuan,621900, PR China.

E-mail: ssxiong@caep.cn

Dr. H. Xu

Key Laboratory of Rare Earth Optoelectronic Materials and Devices of Zhejiang Province, Institute of Optoelectronic Materials and Devices, Collage of Optical and Electronic Technology, China Jiliang University, Hangzhou 310018, People's Republic of China

Prof. Aziz Bakhtiyarovich Ibragimov

Institute of General and Inorganic Chemistry, Uzbekistan Academy of Sciences, M. Ulugbek Str., 77a, Tashkent 100170, Uzbekistan

Prof. Thamraa Alshahrani

Department of Physics, College of Science, Princess Nourah bint Abdulrahman University, Riyadh, 11671, Saudi Arabia

H. Zheng, Dr. L. Li

Key Laboratory of the Ministry of Education for Advanced Catalysis Materials, Zhejiang Key Laboratory of Advanced Catalysis and Adsorption Materials,College of Chemistry and Materials Science, Zhejiang Normal University, Jinhua, China

**Experimental section**

**Materials**

Zinc acetate dihydrate (Zn(OAc)_2_·2H_2_O), benzimidazole (BzIM, C_7_H_6_N_2_) and 5-chlorbenzimidazole (5-Cl-BzIM, C_7_H_5_ClN_2_). All chemicals are purchased from Macklin. The chemicals used in this study are commercially available in analytical grade and can be used without further purification. Pure component gases of Xe (99.999%), Kr (99.999%), N_2_ (99.999%), O_2_ (99.999%), and He (99.999%) were offered by Jingong Co., Ltd. (China).

**Gas adsorption and separation measurements**

The as-synthesized samples were activated under vacuum at 423 K for 12 h (overnight). The adsorption isotherms of the samples were measured at 273 K and 298 K in the 0-1 bar pressure range, using ultrahigh-purity (99.999%) gases including Xe, Kr, N_2_, O_2_ and CO_2_.

The breakthrough experiment used a homemade dynamic gas breakthrough device. A column with a length of 200 mm and an inner diameter of 4.0 mm was filled with about 0.8 g of activated ZIF-7 or ZIF-7-Cl(20). A mixed gas Xe/Kr (20/80, v/v) was used as the feed gas, and the outlet gas was analyzed using a Hiden mass spectrometer (HPR 20).

Based on the mass balance, the kinetic gas adsorption capacities can be determined as follows:

$q_{i}=\frac{C_{i}V}{22.4 \times m} \times\int_{0}^{t} (1-\frac{F}{F_{0}})dt$

Where *q_i_* is the equilibrium adsorption capacity of gas *i* (mmol g^-1^), *C_i_* is the feed gas concentration, *V* is the volumetric feed flow rate (cm^3^ min^-1^), t is the adsorption time (min), *F* and *F_0_* are the inlet and outlet gas molar flow rates, and *m* is the mass of the adsorbent (g).

The selectivity was then calculated according to the equation:

$$S= \frac{{x_{a}}/{X_{b}}}{{y_{a}}/{y_{b}}}$$

where $x_{a}$ and $X_{b}$ are the mole fractions of the gases *a* and *b* in the adsorbed phase and $y_{a}$ and $y_{b}$ are the mole fractions of the gases *a* and *b* in the bulk phase.

**PXRD measurements for stability test of material**

The as-synthesized samples, after being washed with MeOH, approximately 0.2 g for each batch, were immersed in 20 mL of aqueous solutions of PH = 3 (HCl) and pH = 14 (NaOH), deionized water and a range of organic solvents at room temperature for a month. The treated samples were washed with MeOH several times and dried at room temperature before PXRD measurements.

**IAST selectivity**

The IAST selectivity approach was used to evaluate the separation degree of two gas mixtures. The C_2_H_2_ and CO_2_ isotherms were fitted to the single-site Langmuir-Freundlich (LF) and doublt-site Langmuir-Freundlich (DSLF) equation:

$$q=q_{A, sat}\frac{b_{1}p^{c_{A}}}{1+b_{A}p^{c_{A}}}$$

$$q=q_{A, sat}\frac{b_{1}p^{c_{A}}}{1+b_{A}p^{c_{A}}}+q_{B, sat}\frac{b_{B}p^{b_{B}}}{1+b_{B}p^{b_{B}}}$$

where *q* (mmol g^-1^) is the gas capacity of the pertinent gas at pressure P (kPa); qi,sat (mmol g^-1^) represents the saturated uptakes of site A and site B; *b_A_* and *b_B_* (kPa-1)) are the affinity coefficients of the two corresponding sites; *c_A_* and *c_B_* are the system heterogeneity of the two respective sites.

For the separation of binary mixtures of components Xe/Kr (20/80, v/v), Xe/N_2_ (1/99, v/v) and Xe/O_2_ (1/99, v/v), the adsorption selectivity is defined by the following equation:

$S_{ads}=\frac{{q_{A}}/{q_{B}}}{{p_{A}}/{p_{B}}}$

In the equation, *q_A_* and *q_B_* are the proposed molar fractions in the adsorbed phase in equilibrium with the bulk gas phase with partial pressures *p_A_* and *p_B_*.

**Henry’s selectivity**

The Henry’s coefficient value of both gases is obtained from the single-component isotherm that was fit by Langmuir equation:

$$q= q_{sat}\frac{bp}{1+bp}$$

where *q* is the amount of adsorbates on the solid, *P* is the corresponding partial pressure in the gas phase, *qsat* represents the saturation adsorption capacity and *b* is the equilibrium constant or Langmuir constant.

The Henry’s coefficient *H* is defined as:

$$H= q_{sat}b$$

In the very low-pressure range, the gas adsorbed amount q increases linearly with pressure P:

$$q=HP$$

At conditions where each adsorbed component is accurately described by Henry’s law, interactions between adsorbed molecules are (by definition) negligible. In this limit, the ratio of Henry’s coefficient of pure Xe and Kr, was used to evaluate the adsorption selectivity (α).

$$\alpha= \frac{H_{Xe}}{H_{Kr}}$$

**The isosteric enthalpy of adsorption (Q_st_)**

The isosteric enthalpy of adsorption (Q_st_) for Xe and Kr on ZIF-7 and ZIF-7-Cl(x) was collected at two different temperatures, 273 K and 298 K, based on viral fitting using the Clausius-Clapeyron equation:

$$\ln P=-\frac{Q_{\mathrm{st}}}{R}\frac{1}{T}+C$$

where Q_st_ (kJ mol^-1^) represents the isosteric heat of gas, T (K) is the temperature, R is ideal gas constant (8.314 J mol^-1^ K^-1^), P (kPa) is the equilibrium pressure, C is a constant.

**XRD Refinement**

Refinement was performed using Topas v5.0. The background was described using a 24th-order Chebyshev polynomial, the peak shape using a Pseudo-viogt function, and the preferred orientation of the sample using a 4th-order spherical cofunction. The Rietveld refinement process involved sequentially refining the scale bar, atomic coordinates, isotropic temperature factor, atomic occupancy, and preferred orientation.

**Molecular simulation**

Grand Canonical Monte Carlo (GCMC) simulations of ZIF-7-Cl(20) were performed using the adsorption module. During the simulation, the ZIF-7-Cl(20) framework is considered rigid. Partial charges for atoms of ZIF-7-Cl(20) were derived from the QEq method and QEq_neutral1.0 parameter. The simulations were carried out at 298 K, adopting the locate task and fixed pressure, the Metropolis method in the Sorption module, and the universal force field (UFF). The partial charges on the atoms of Xe were also derived from the QEq method. The interaction energy between Xe atoms and the framework was computed through the Coulomb and Lennard-Jones 6–12 (LJ) potentials. The cutoff radius was chosen as 12.5 Å for the LJ potential and the long-range electrostatic interactions were handled using the Ewald & Group summation method. The loading steps and the equilibration steps were 1 × 10^6^, the production steps were 1 × 10^6^.

**Figure and Table**

**
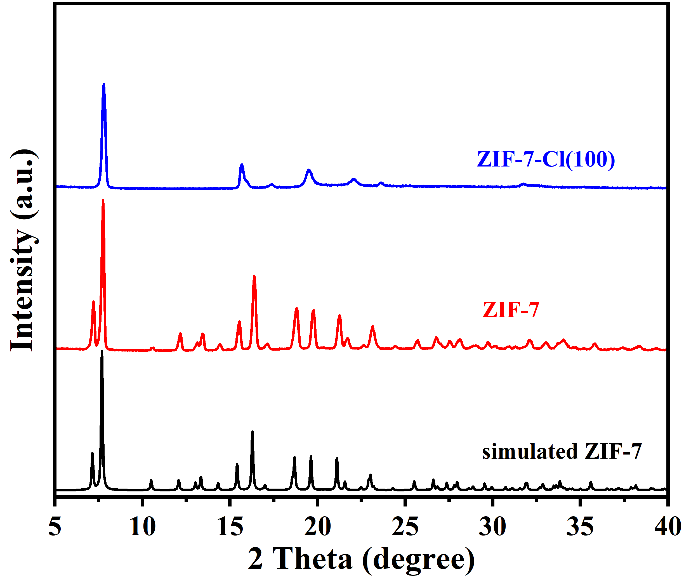
**

**Figure S1.** PXRD patterns of ZIF-7 and ZIF-7-Cl(100).


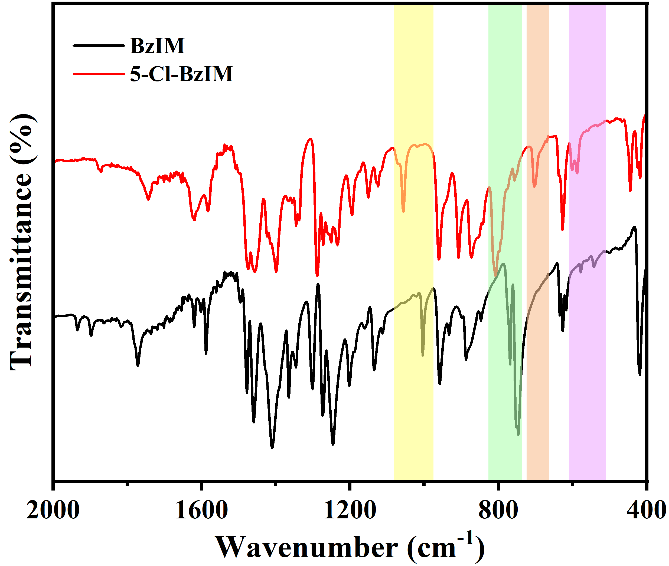


**Figure S2.** FT-IR spectra of organic linkers zIM and 5-ClB zIm.

**
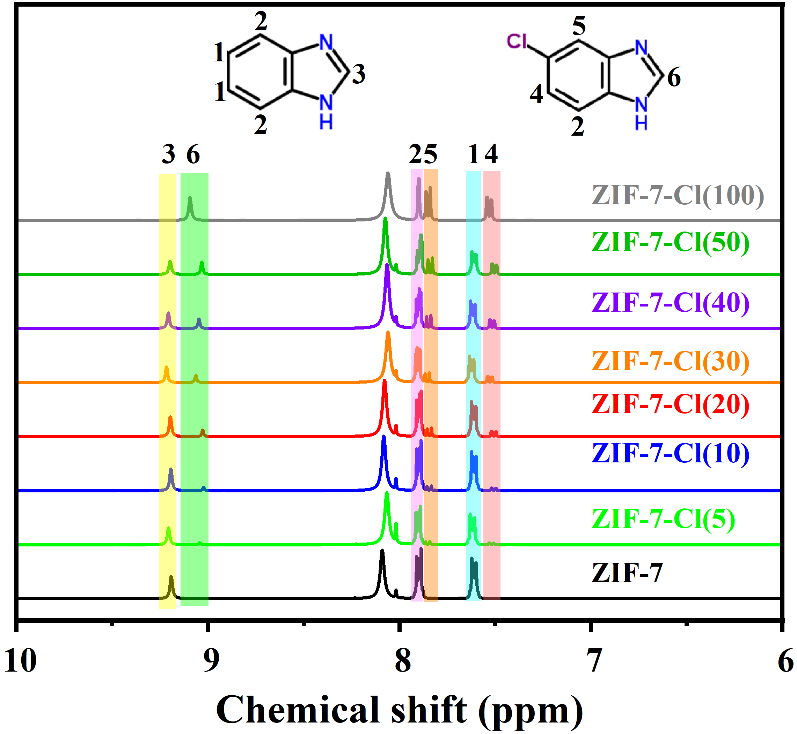
**

**Figure S3.** 1H NMR of ZIF-7 and ZIF-7-Cl(x).


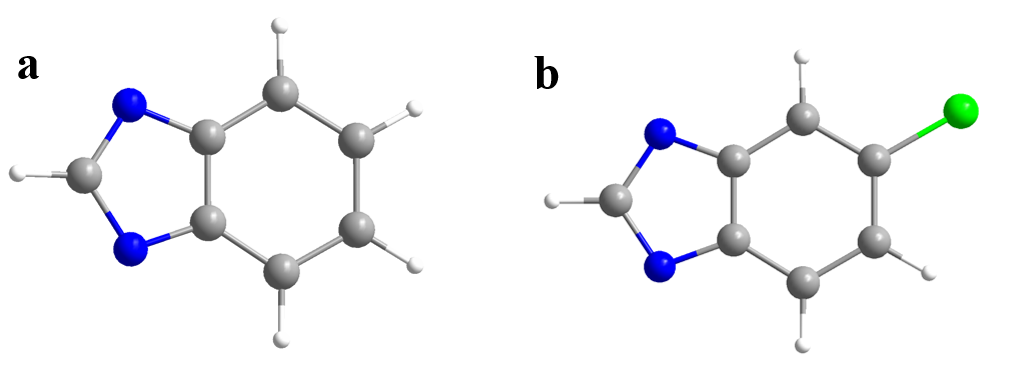


**Figure S4.** Simplified ball-and-stick models of BzIM(a) and 5-ClBzIM(b) within a MOF framework. (Color codes: C, gray; N, blue; H, white; Cl, ​​green)

Based on the ball-and-stick models of BzIM and 5-ClBzIM in the framework, we can obtain that C/H = 7/5 in BzIM and C/H = 7/4 in 5-ClBzIM. The EA results show that the mass percentages of C and H in ZIF-7 are 52.72% and 3.13%, respectively. Therefore, we can obtain:

$$\frac{{52.72}/{M_{C}}}{3.13/M_{H}}\approx\frac{7}{5}$$

In ZIF-7-Cl(20), the proportion of H replaced by Cl in the framework can be calculated above, and thus the content of 5-ClBzIM in the framework can be obtained:

$$\frac{{50.83}/{M_{C}}}{2.92/M_{H}}\approx\frac{7}{x}$$

The calculated value of x is 4.826. Since only one hydrogen atom is substituted in 5-ClBzIM, the actual content of 5-ClBzIM in the framework is 5 - x * 100% = 17.4%.


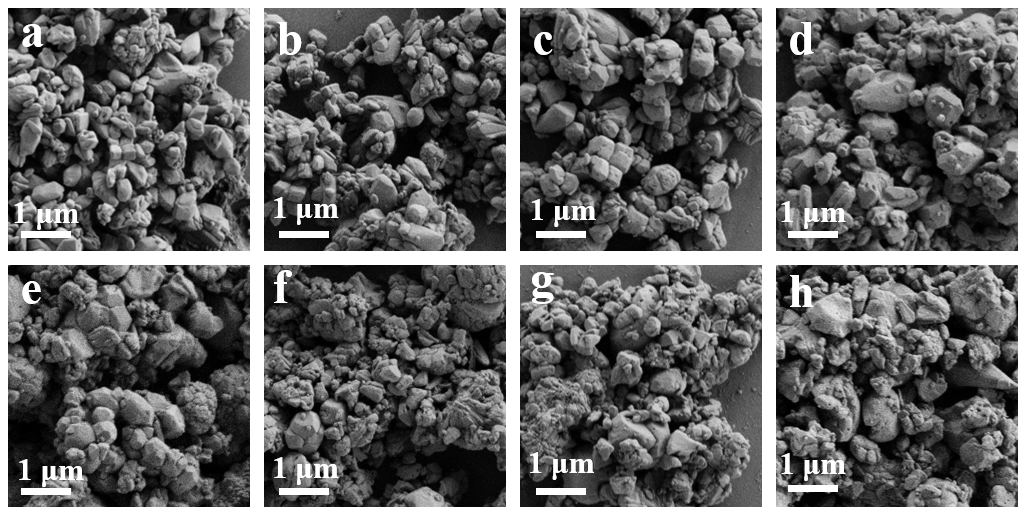


**Figure S5.** SEM image of adsorbent: a) ZIF-7; b) ZIF-7-Cl(5); c) ZIF-7-Cl(10); d) ZIF-7-Cl(20); e) ZIF-7-Cl(30); f) ZIF-7-Cl(40); g) ZIF-7-Cl(50); h) ZIF-7-Cl(100).


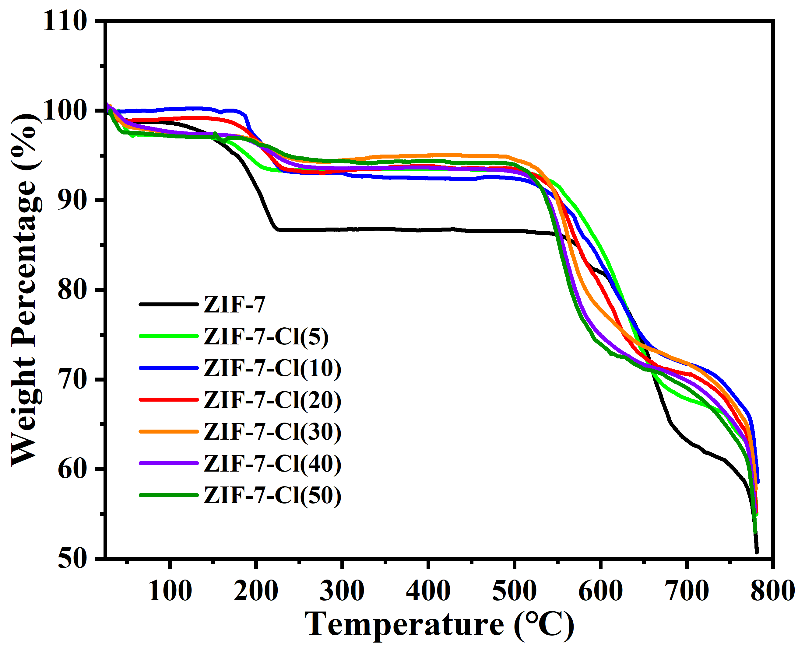


**Figure S6.** The TGA curves of ZIF-7 and ZIF-7-Cl(x).

**
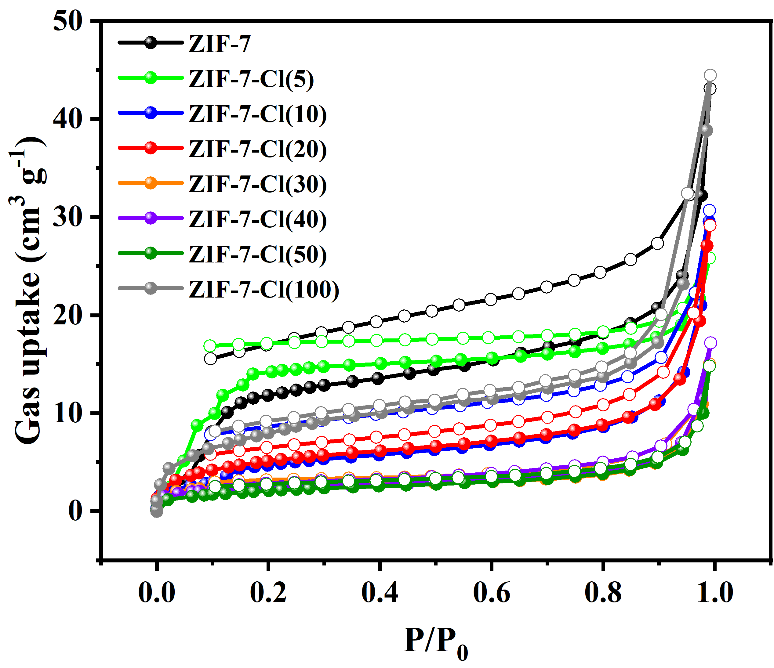
**

**Figure S7.** N_2_ adsorption isotherms of ZIF-7 and ZIF-7-Cl(x) at 77 K.

**
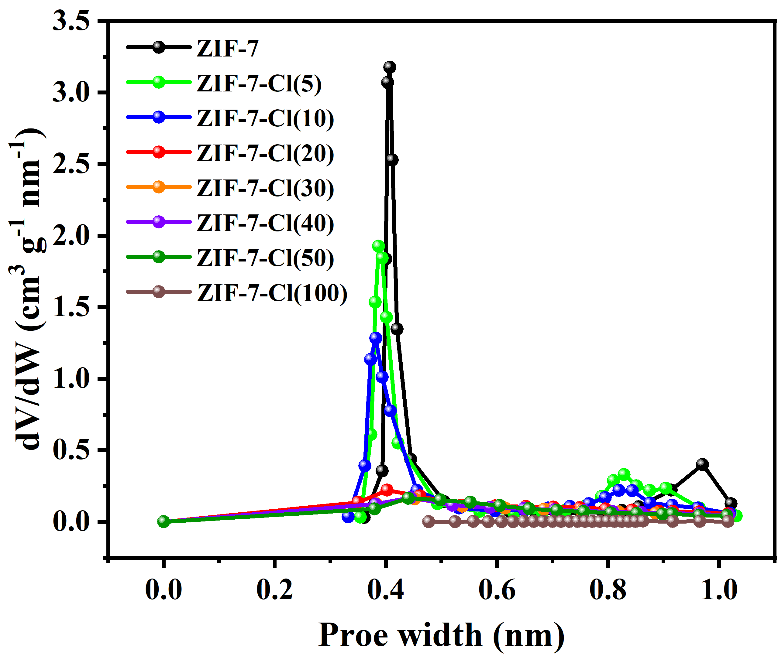
**

**Figure S8.**  The pore size distribution was calculated using the H-K model based on the CO_2_ adsorption isotherm at 195 K.

**
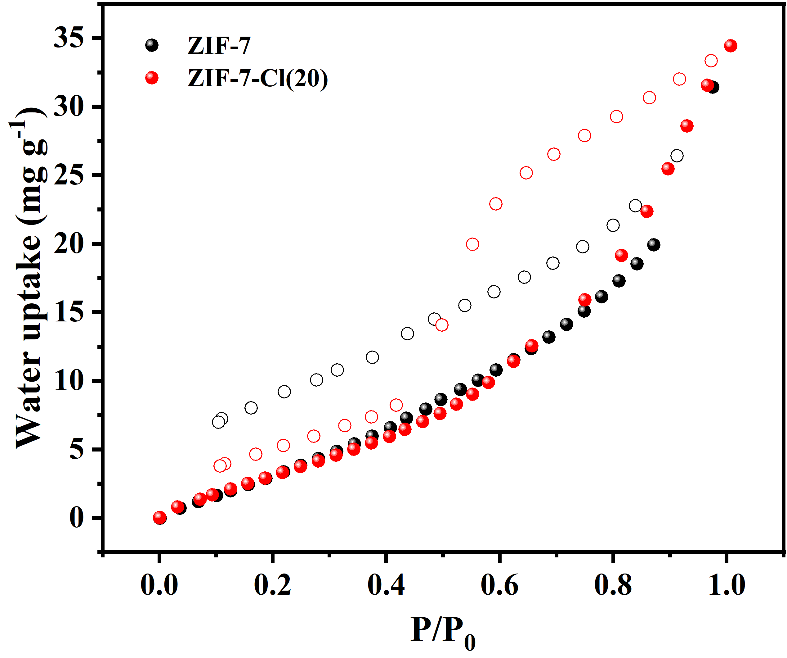
**

**Figure S9.** Water adsorption isotherms of ZIF-7 and ZIF-7-Cl(20) at 298K.


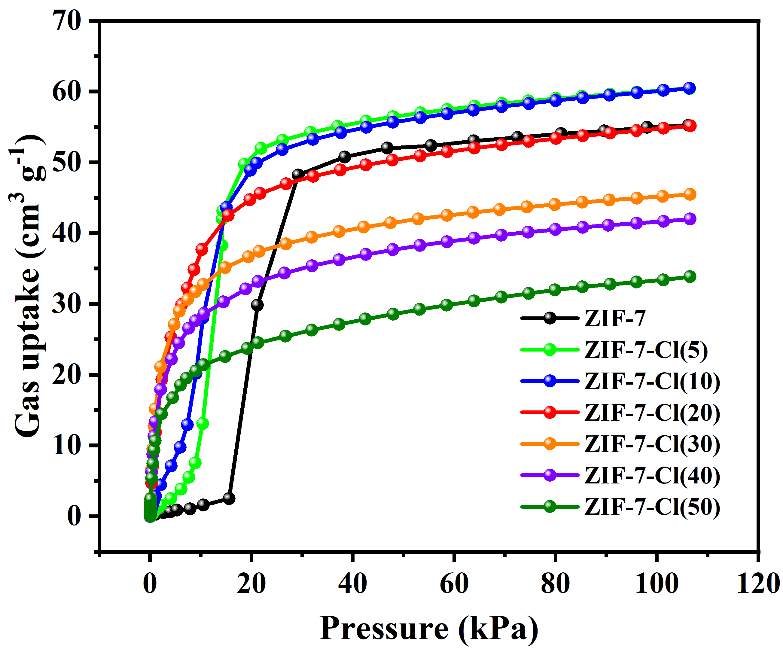


**Figure S10.** Xe adsorption isotherms of ZIF-7 and ZIF-7-Cl(x) at 273K.


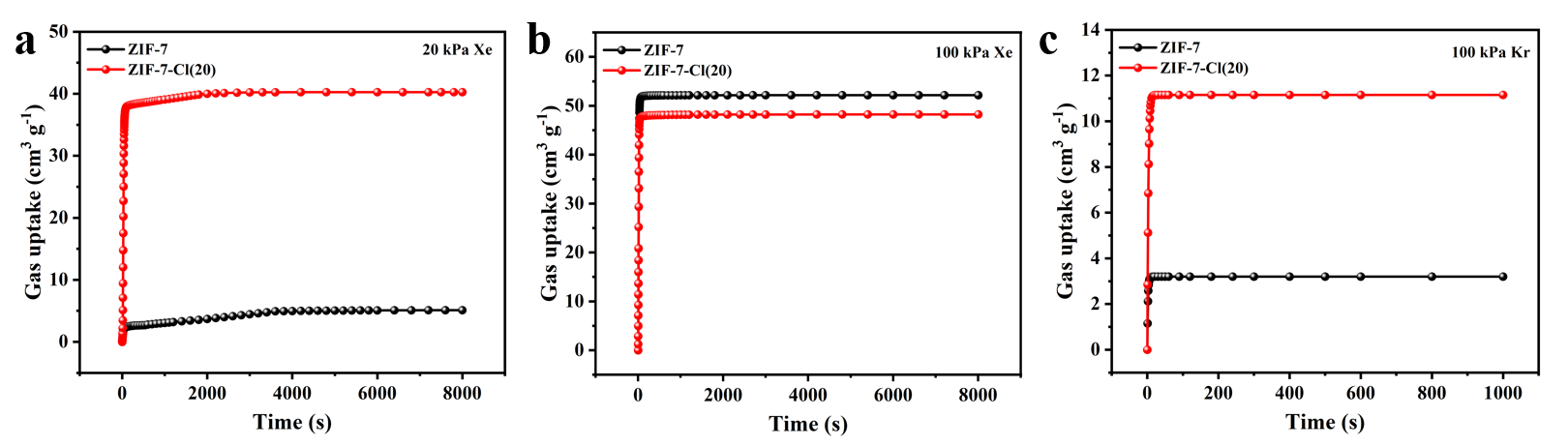


**Figure S11.** Kinetic adsorption curves of ZIF-7 and ZIF-7-Cl(20) under different Xe pressures: (a) 20 kPa; (b) 100 kPa; Kinetic adsorption curves of ZIF-7 and ZIF-7-Cl(20) under 100 kPa Kr.


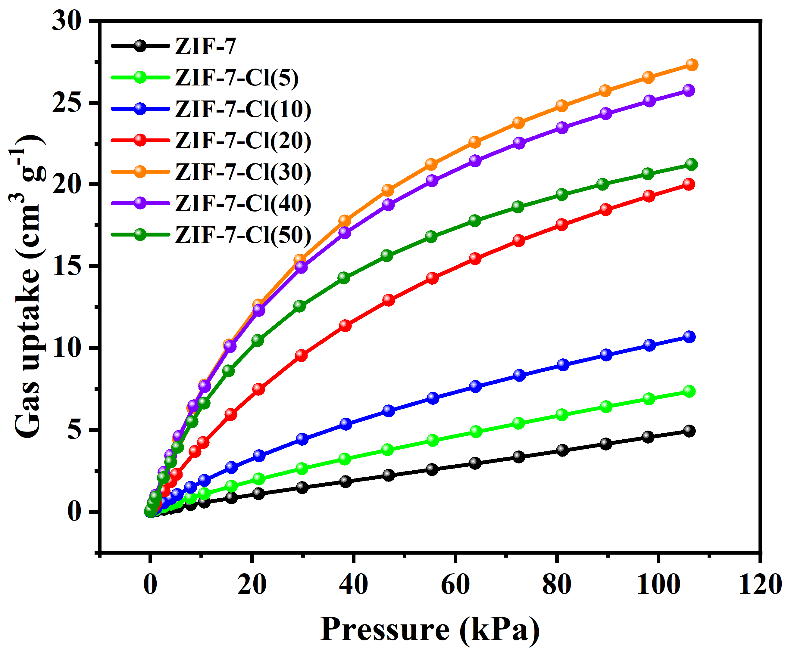


**Figure S12.** Kr adsorption isotherms of ZIF-7 and ZIF-7-Cl(x) at 273K.


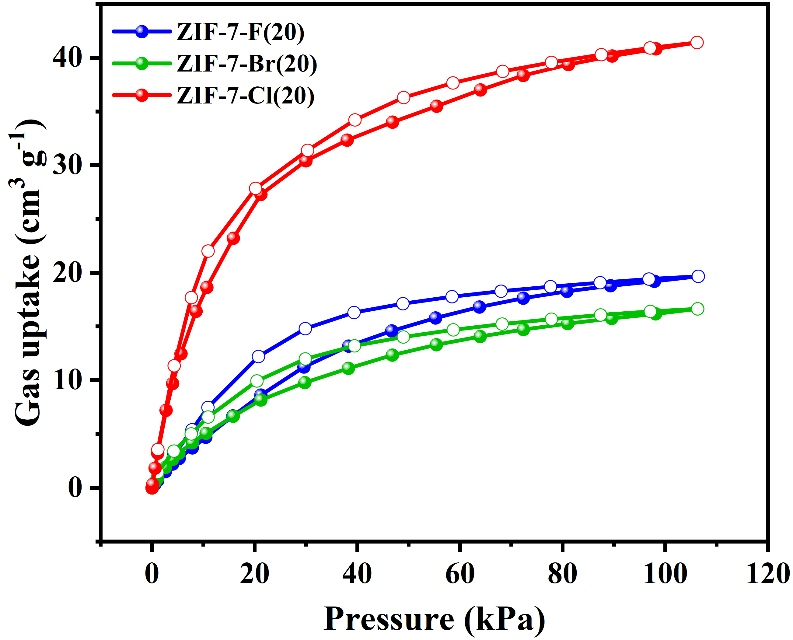


**Figure S13.** CO_2_ adsorption isotherms of ZIF-7-X(20) (X = F, Cl and Br) at 298K.


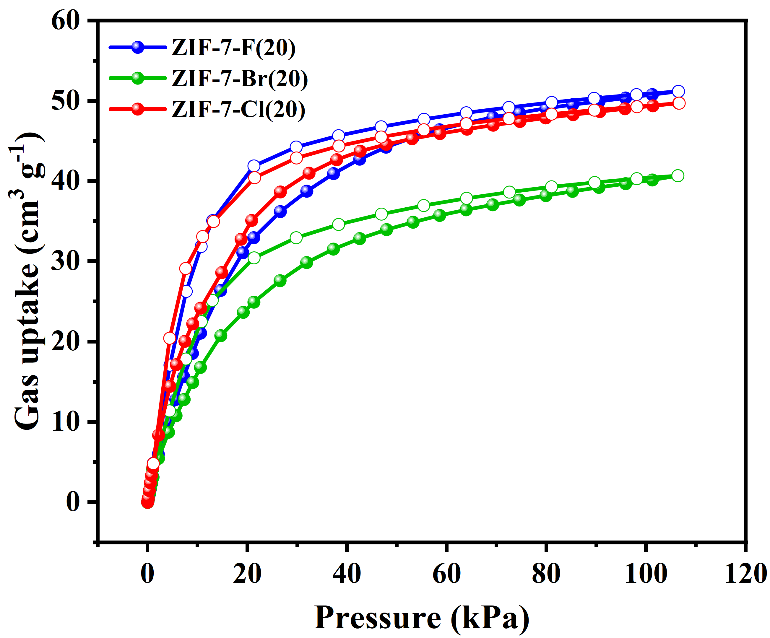


**Figure S14.** Xe adsorption isotherms of ZIF-7-X(20) (X = F, Cl and Br) at 298K.


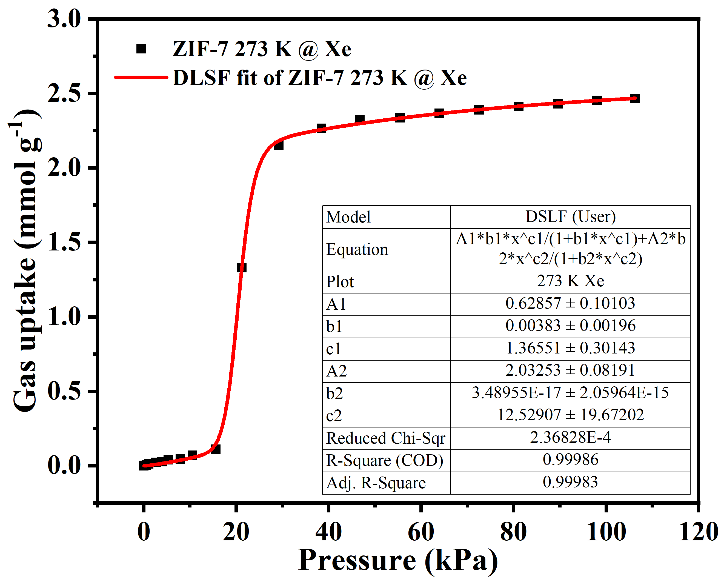


**Figure S15.** The Langmuir-Freundlich fitting results of Xe isotherm at 273 K in ZIF-7.


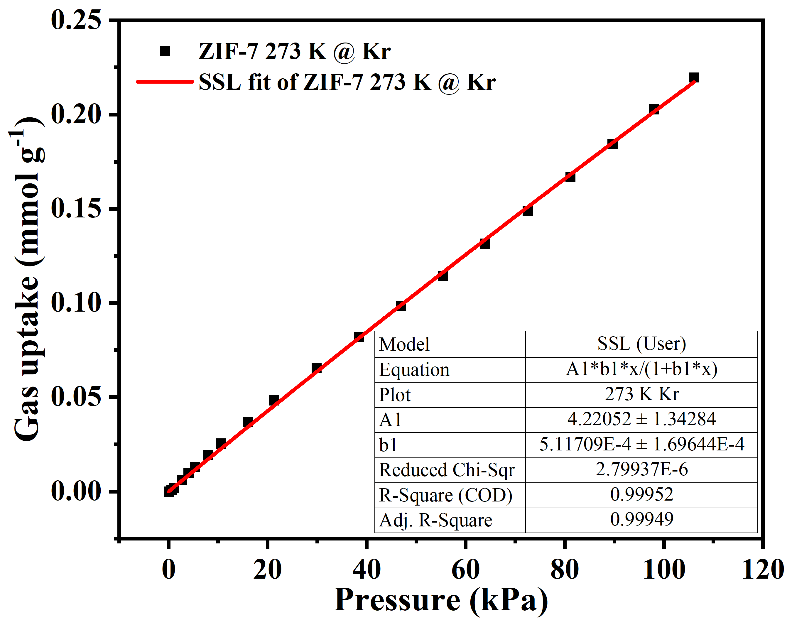


**Figure S16.** The Langmuir fitting results of Kr isotherm at 273 K in ZIF-7.


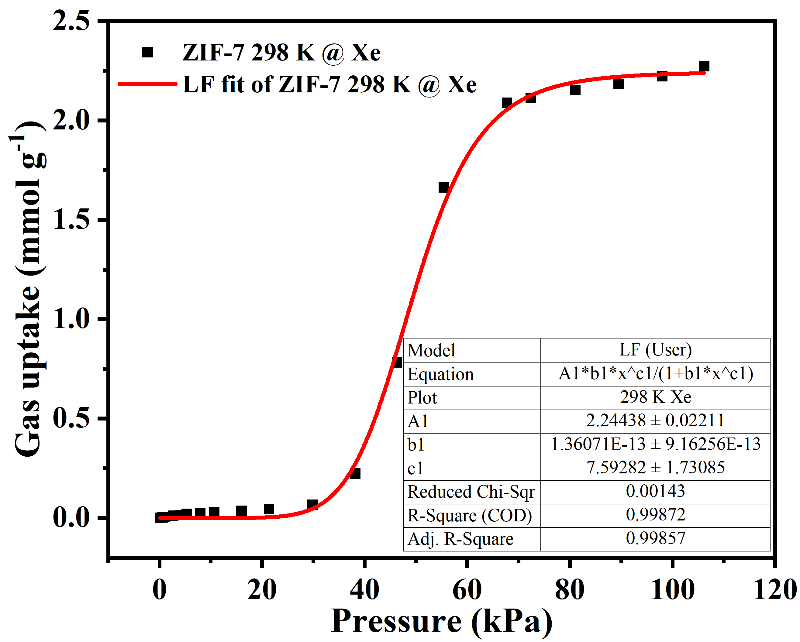


**Figure S17.** The Langmuir-Freundlich fitting results of Xe isotherm at 298 K in ZIF-7.


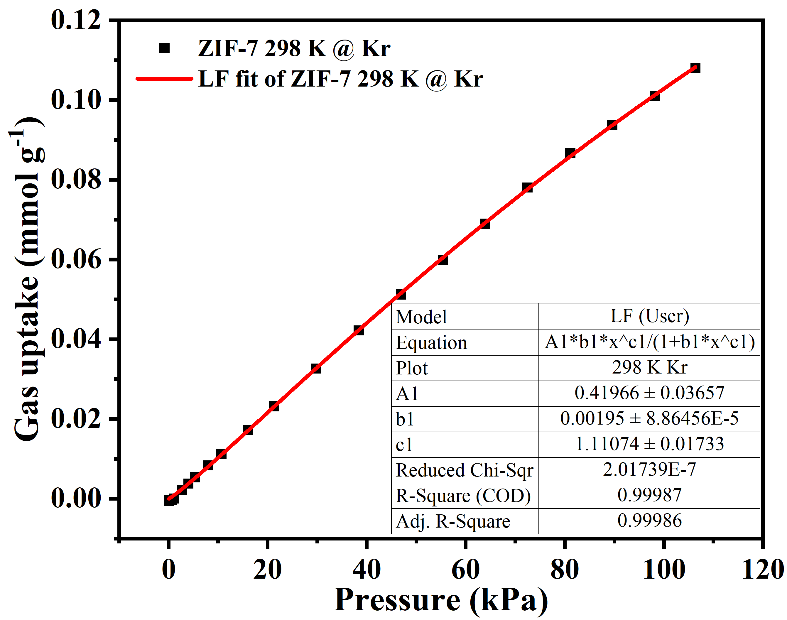


**Figure S18.** The Langmuir-Freundlich fitting results of Kr isotherm at 298 K in ZIF-7.


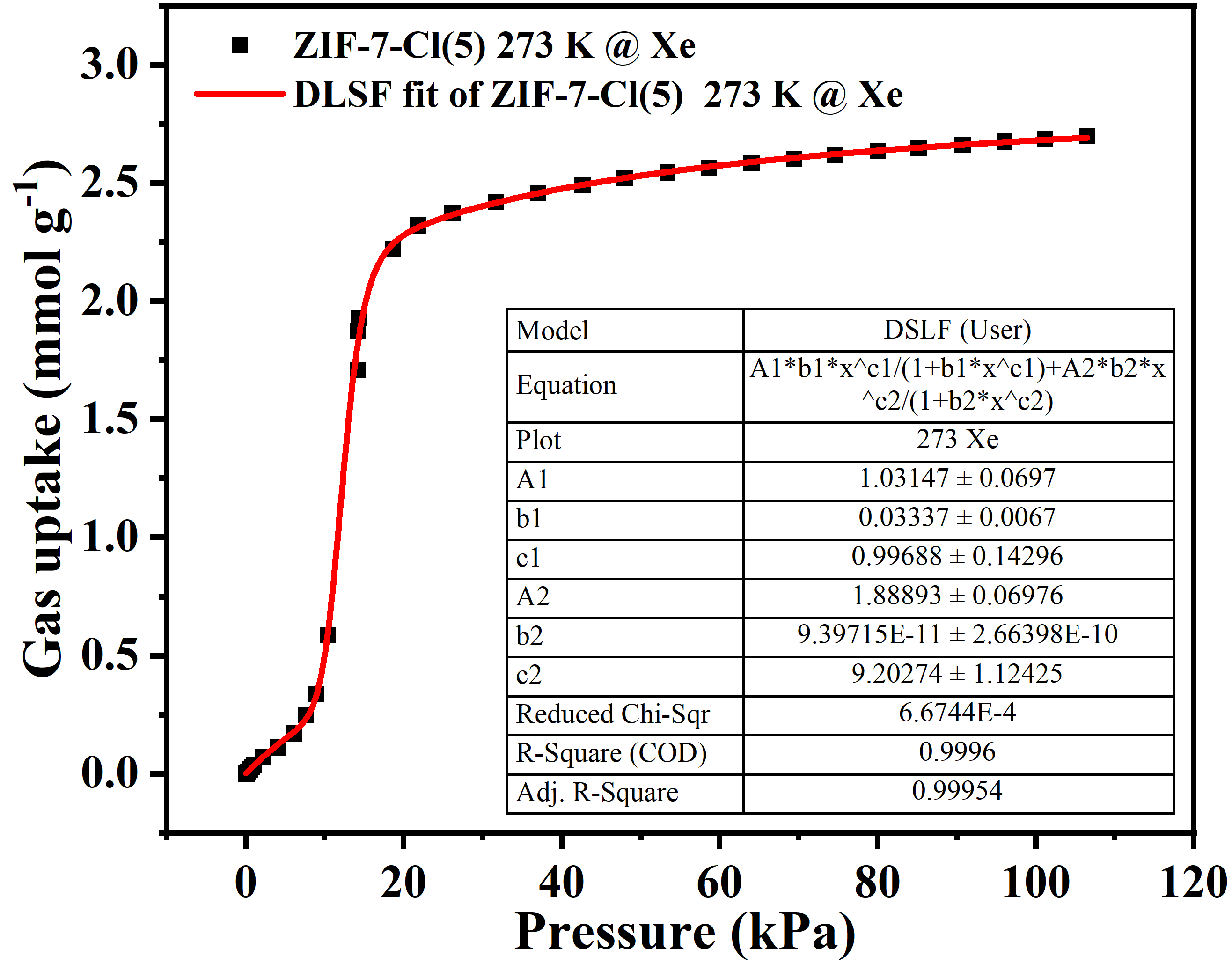


**Figure S19.** The Langmuir-Freundlich fitting results of Xe isotherm at 273 K in ZIF-7-Cl(5).


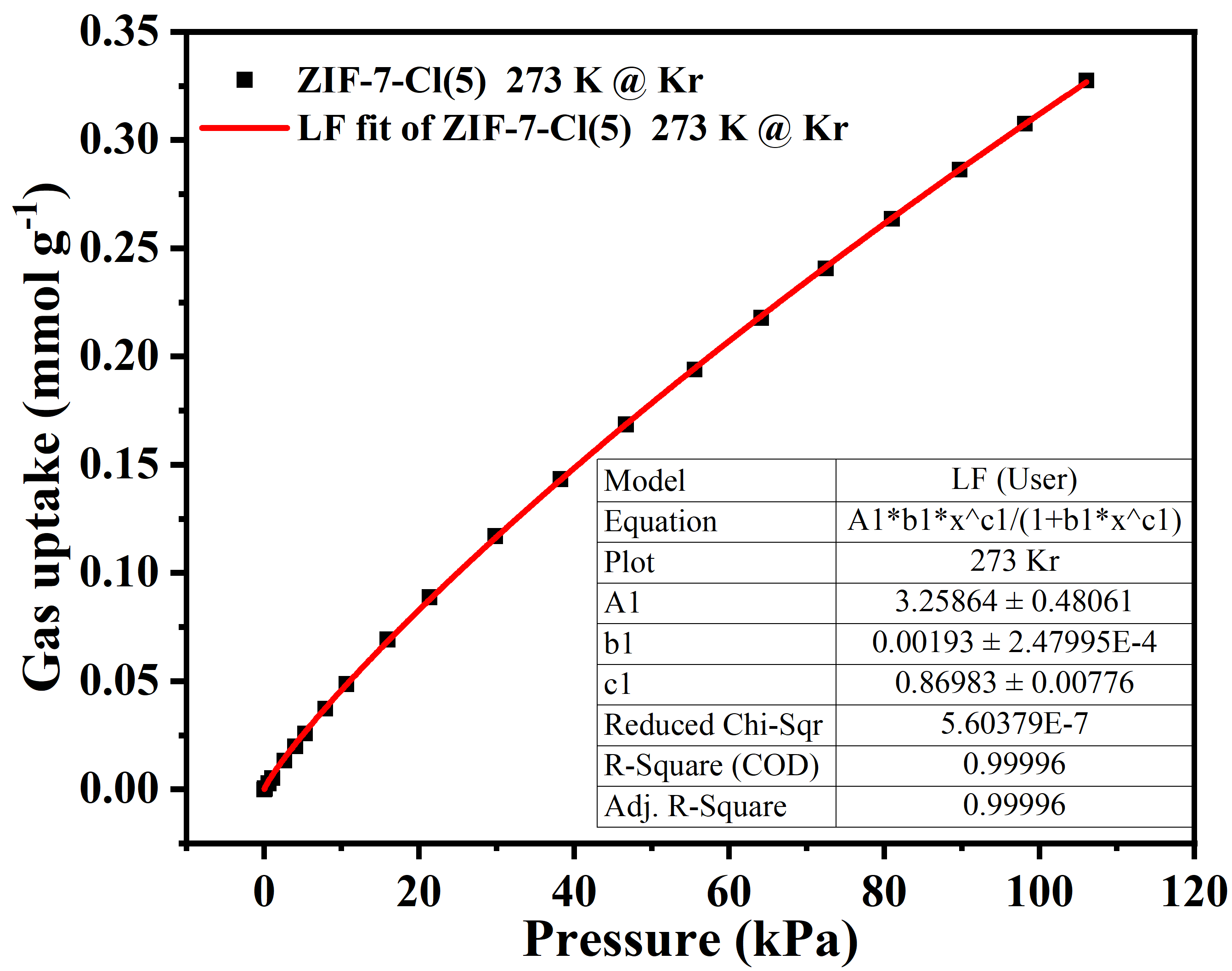


**Figure S20.** The Langmuir-Freundlich fitting results of Kr isotherm at 273 K in ZIF-7-Cl(5).


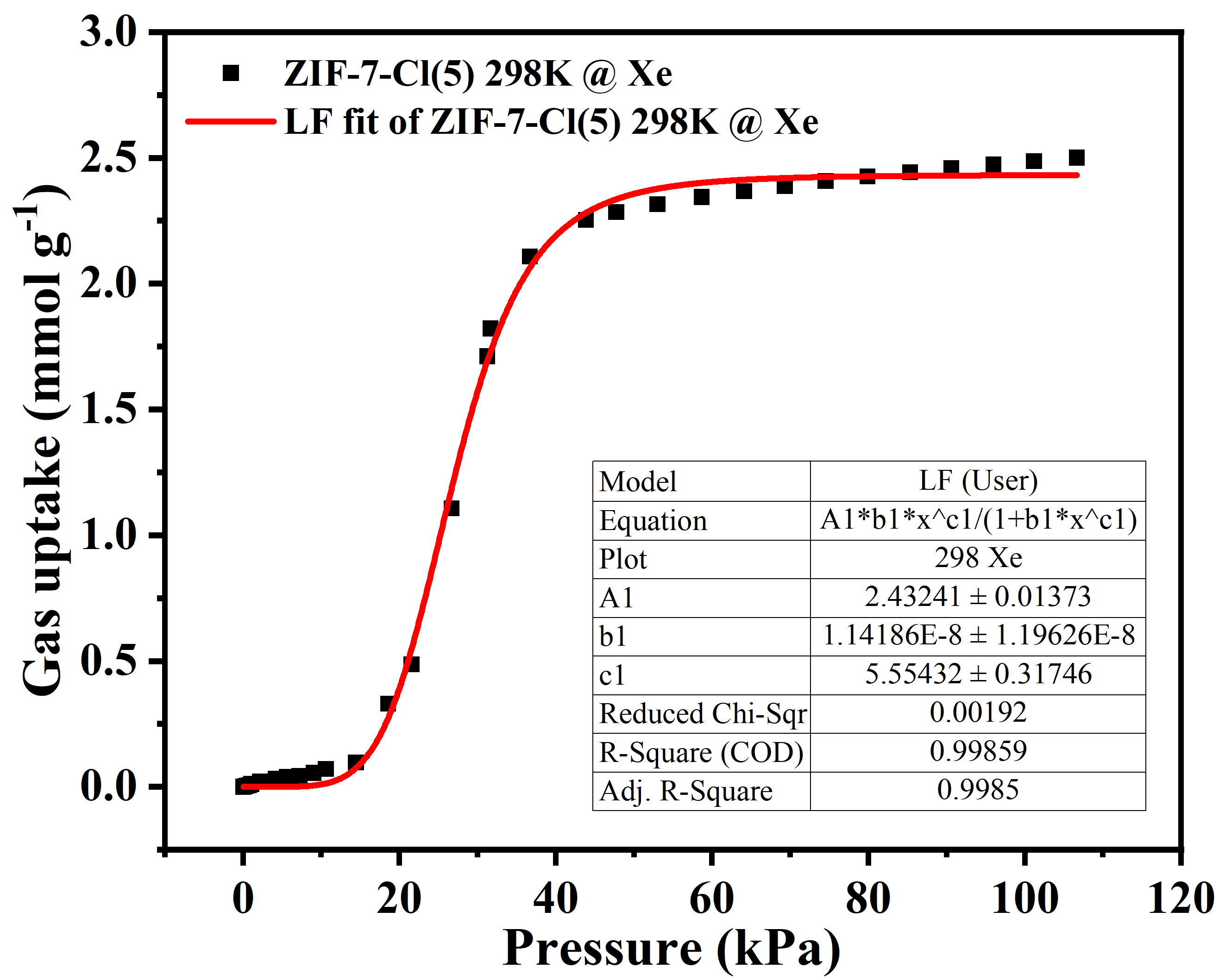


**Figure S21.** The Langmuir-Freundlich fitting results of Xe isotherm at 298 K in ZIF-7-Cl(5).


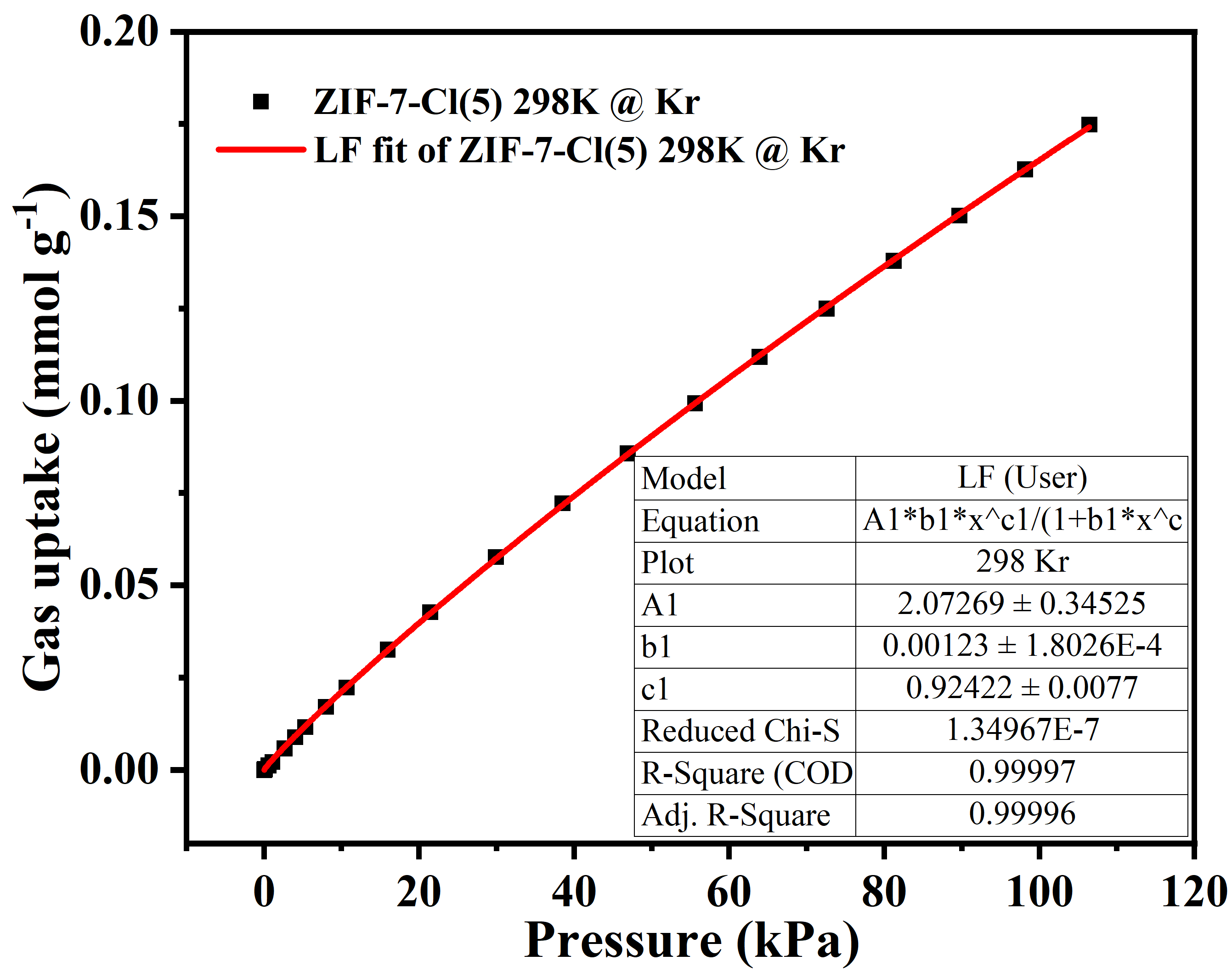


**Figure S22.** The Langmuir-Freundlich fitting results of Kr isotherm at 298 K in ZIF-7-Cl(5).


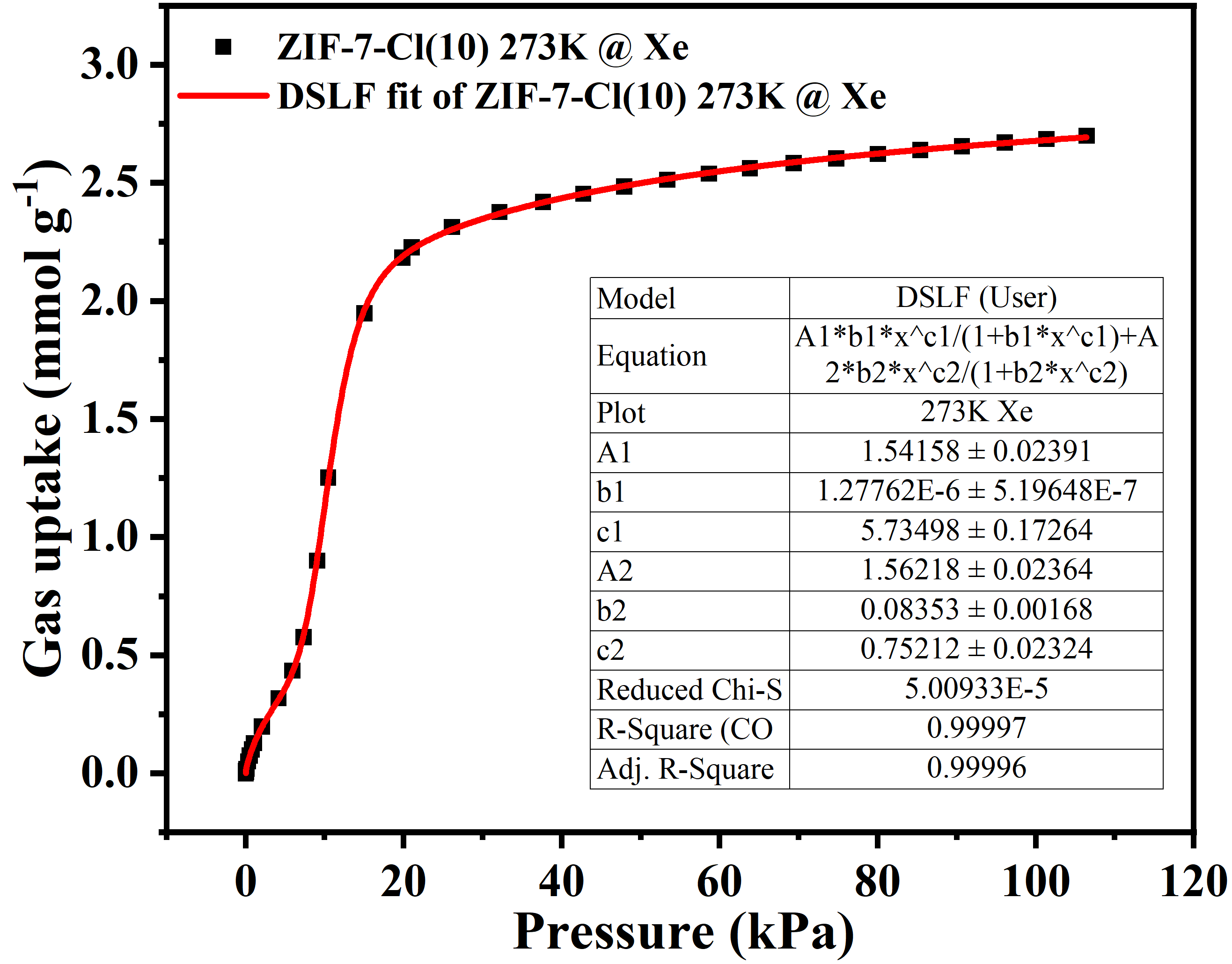


**Figure S23.** The Langmuir-Freundlich fitting results of Xe isotherm at 273 K in ZIF-7-Cl(10).


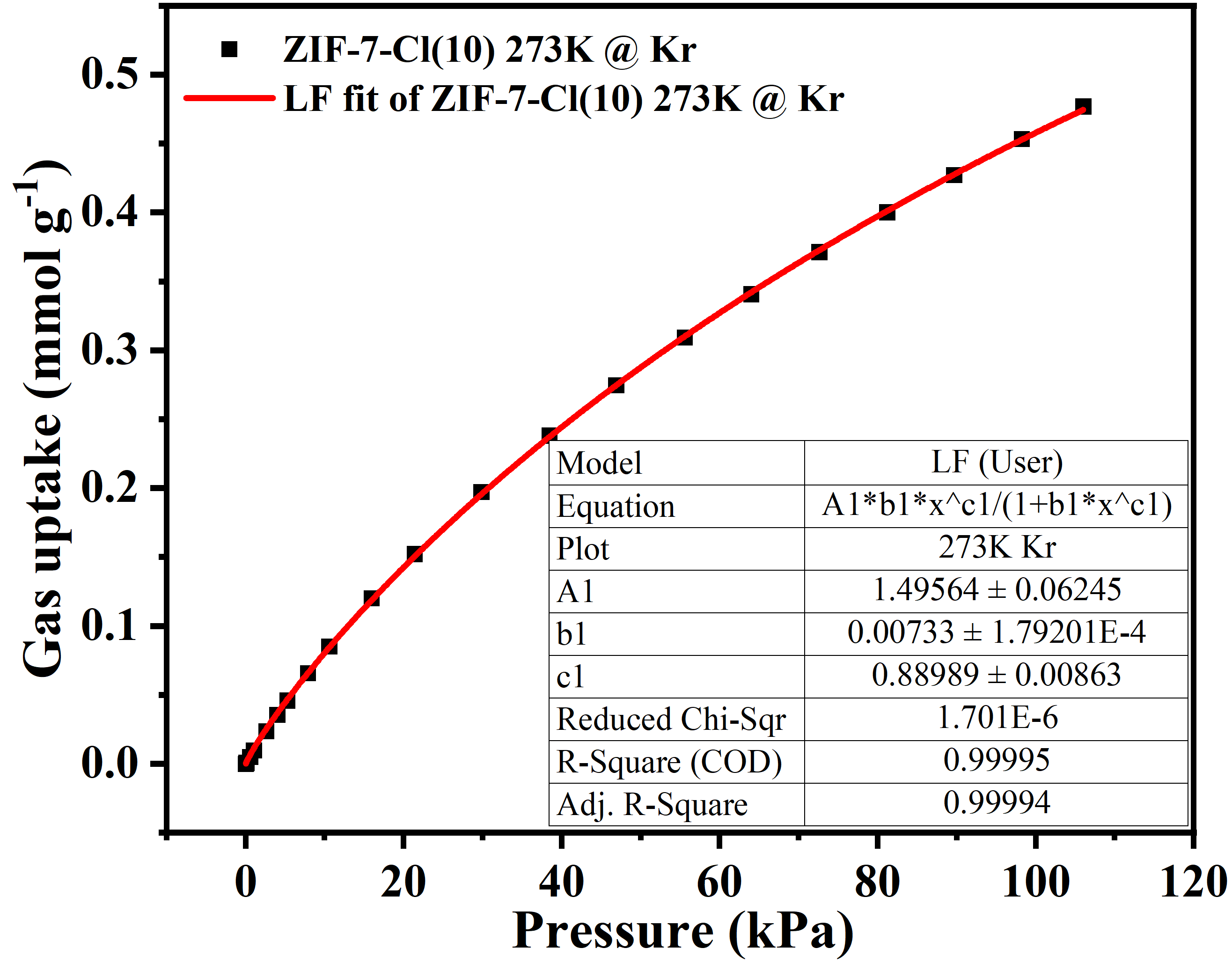


**Figure S24.** The Langmuir-Freundlich fitting results of Kr isotherm at 273 K in ZIF-7-Cl(10).


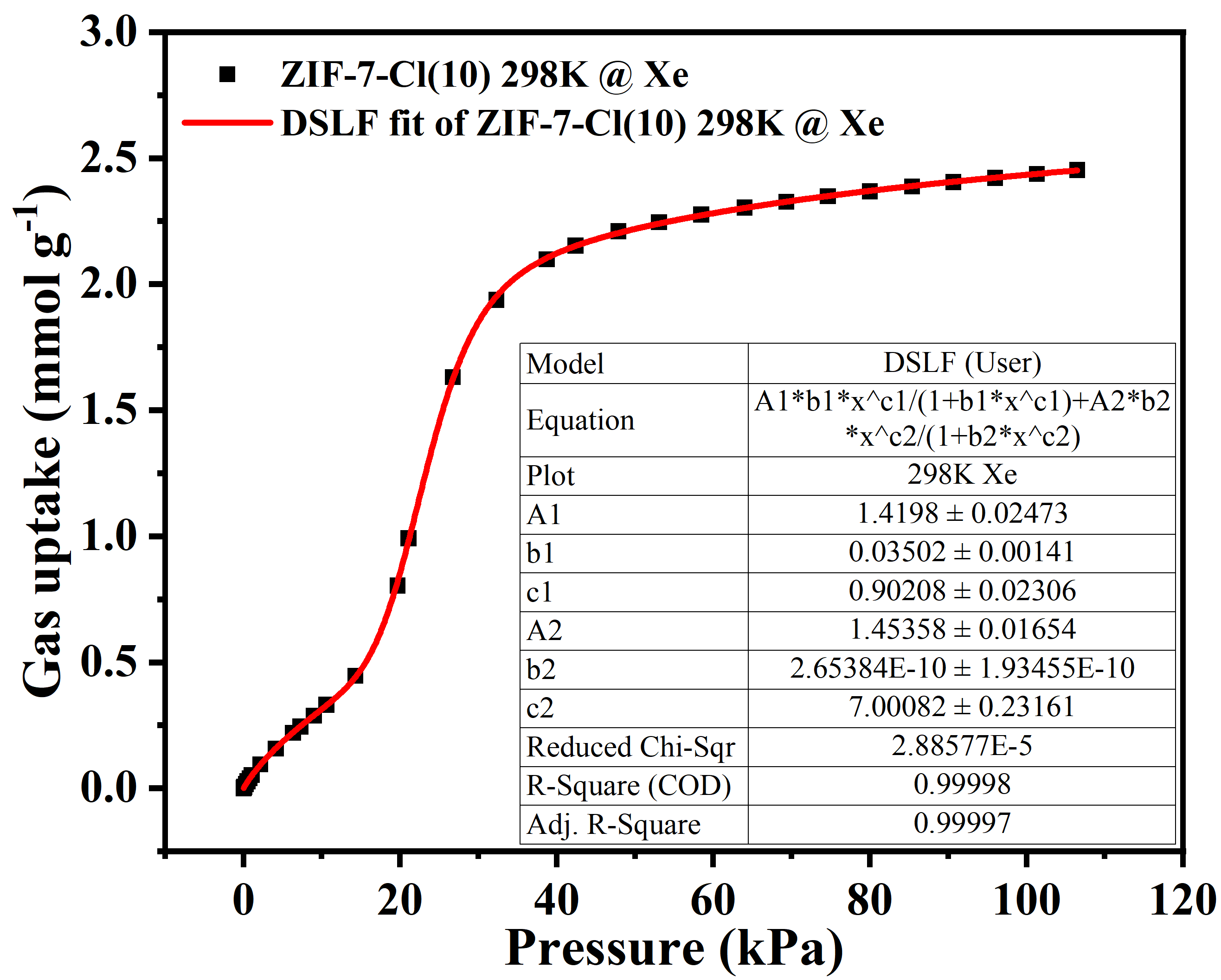


**Figure S25.** The Langmuir-Freundlich fitting results of Xe isotherm at 298 K in ZIF-7-Cl(10).


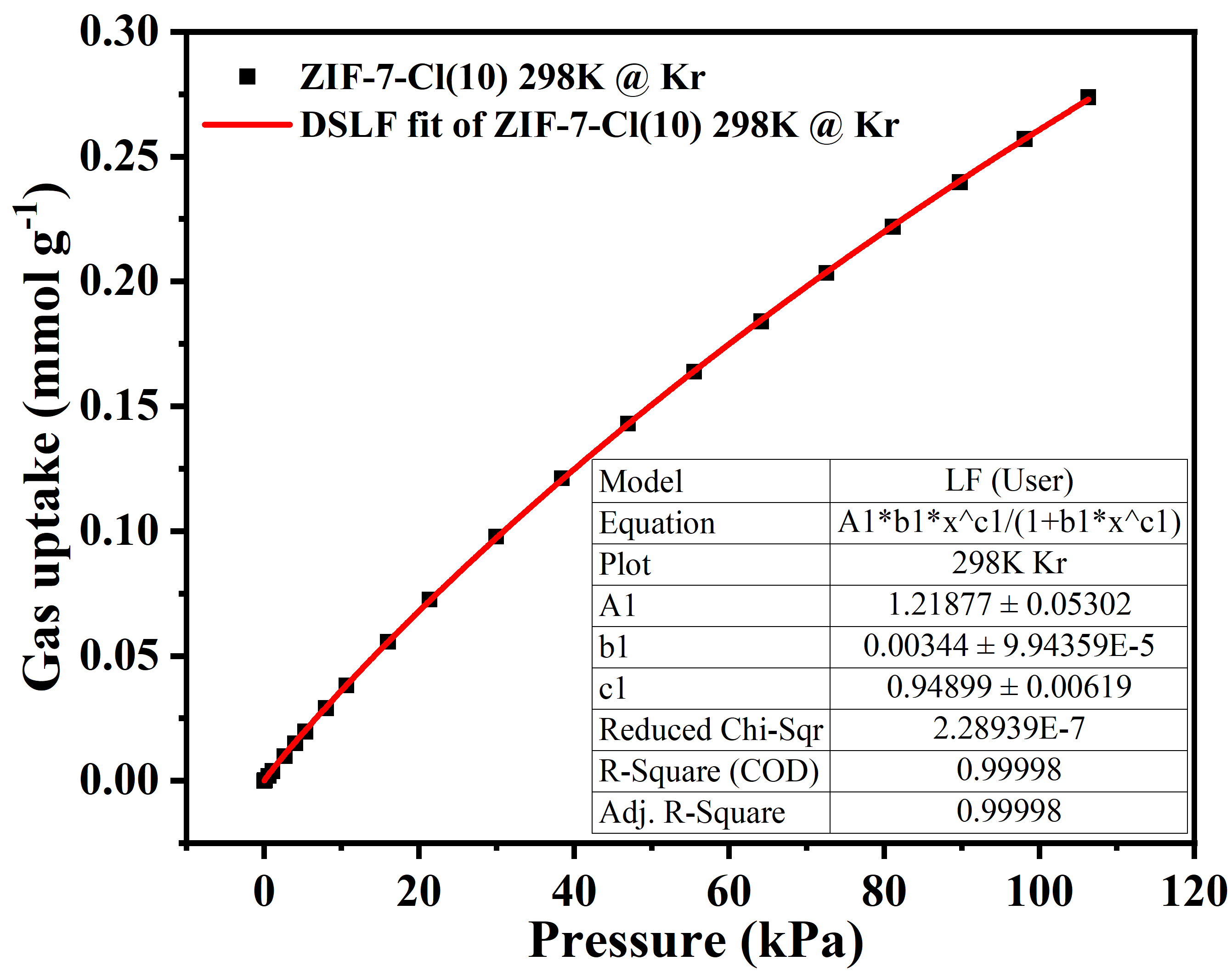


**Figure S26.** The Langmuir-Freundlich fitting results of Kr isotherm at 298 K in ZIF-7-Cl(10).


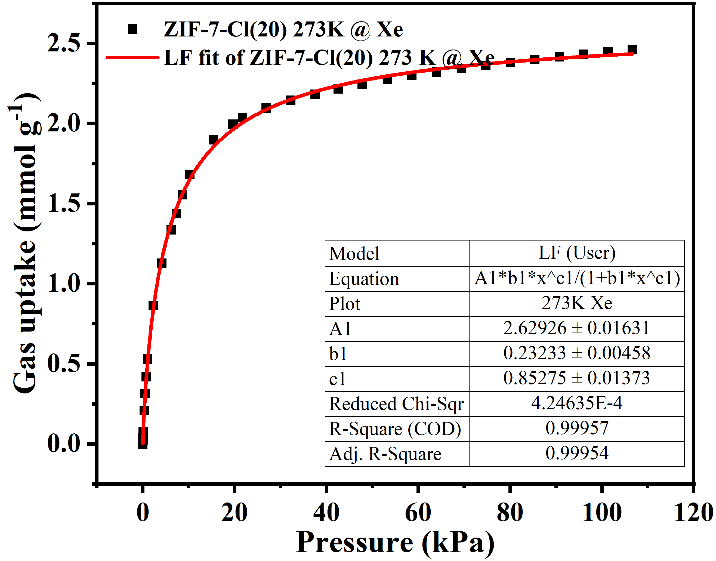


**Figure S27.** The Langmuir-Freundlich fitting results of Xe isotherm at 273 K in ZIF-7-Cl(20).


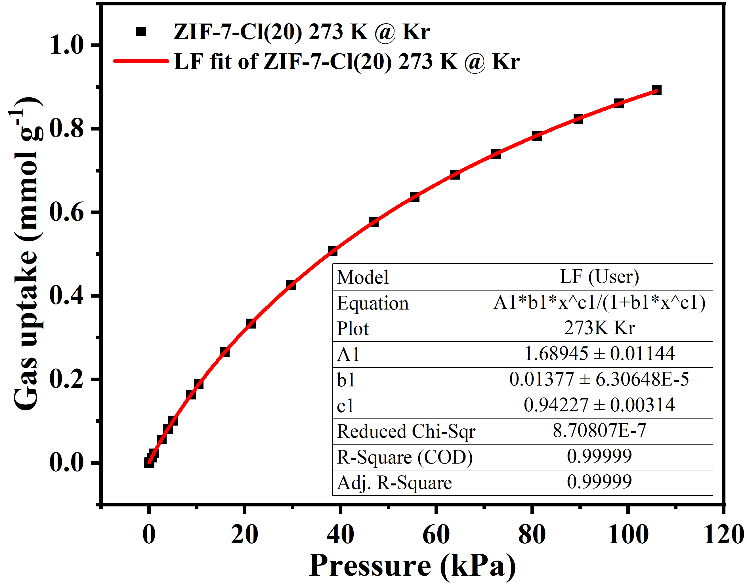


**Figure S28.** The Langmuir-Freundlich fitting results of Kr isotherm at 273 K in ZIF-7-Cl(20).


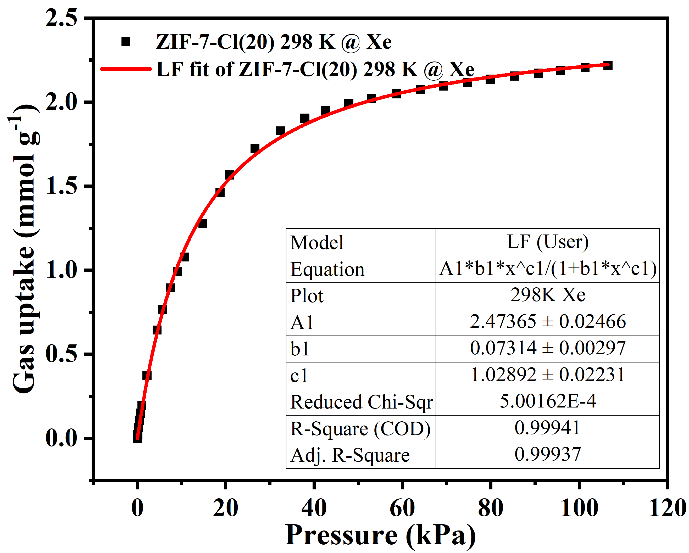


**Figure S29.** The Langmuir-Freundlich fitting results of Xe isotherm at 298 K in ZIF-7-Cl(20).


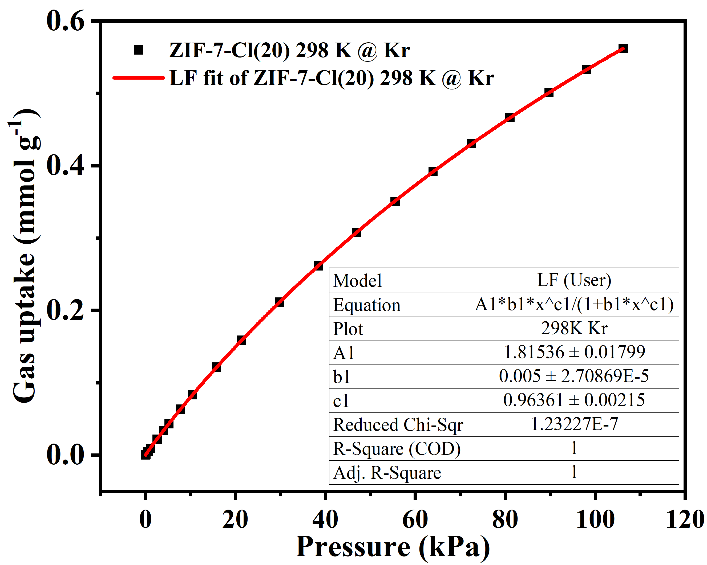


**Figure S30.** The Langmuir-Freundlich fitting results of Kr isotherm at 298 K in ZIF-7-Cl(20).


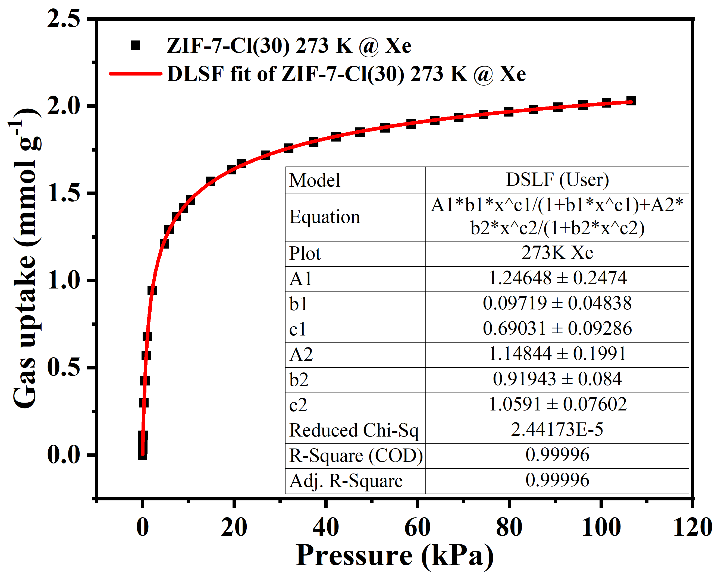


**Figure S31.** The Langmuir-Freundlich fitting results of Xe isotherm at 273 K in ZIF-7-Cl(30).


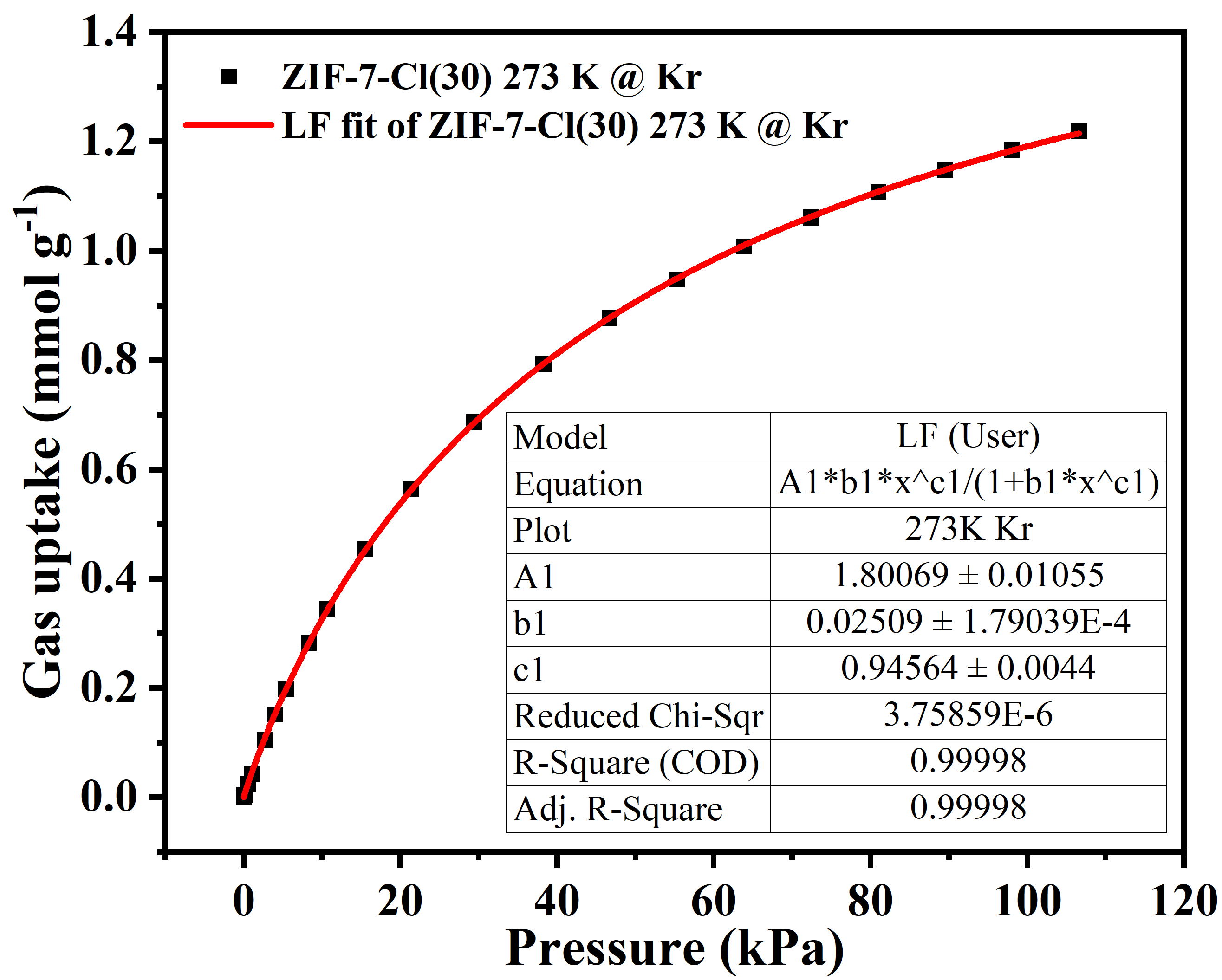


**Figure S32.** The Langmuir-Freundlich fitting results of Kr isotherm at 273 K in ZIF-7-Cl(30).


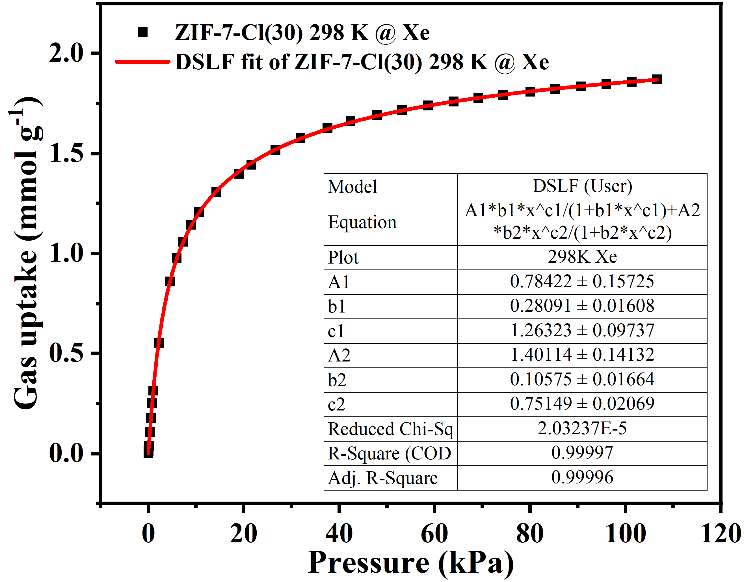


**Figure S33.** The Langmuir-Freundlich fitting results of Xe isotherm at 298 K in ZIF-7-Cl(30).


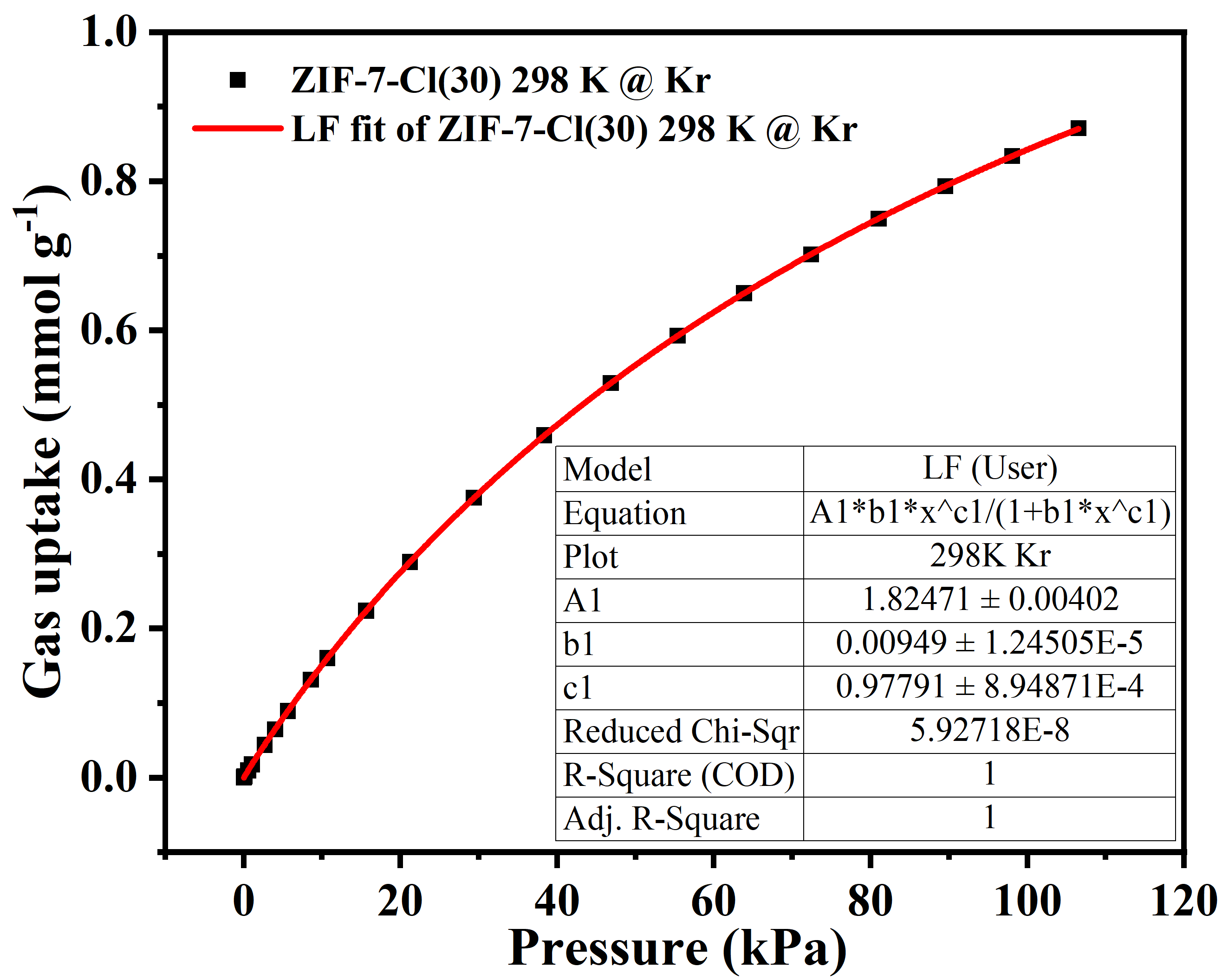


**Figure S34.** The Langmuir-Freundlich fitting results of Kr isotherm at 298 K in ZIF-7-Cl(30).


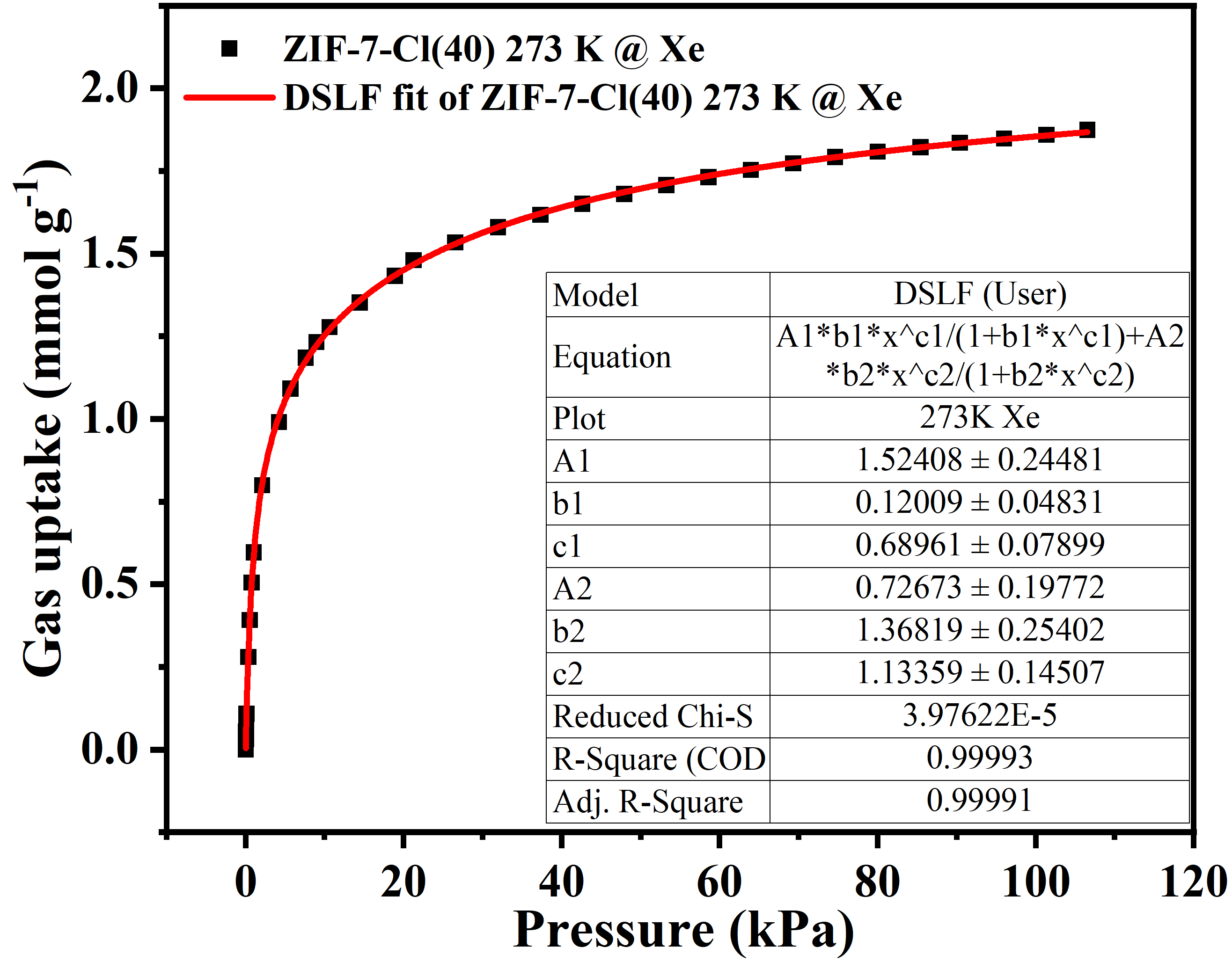


**Figure S35.** The Langmuir-Freundlich fitting results of Xe isotherm at 273 K in ZIF-7-Cl(40).


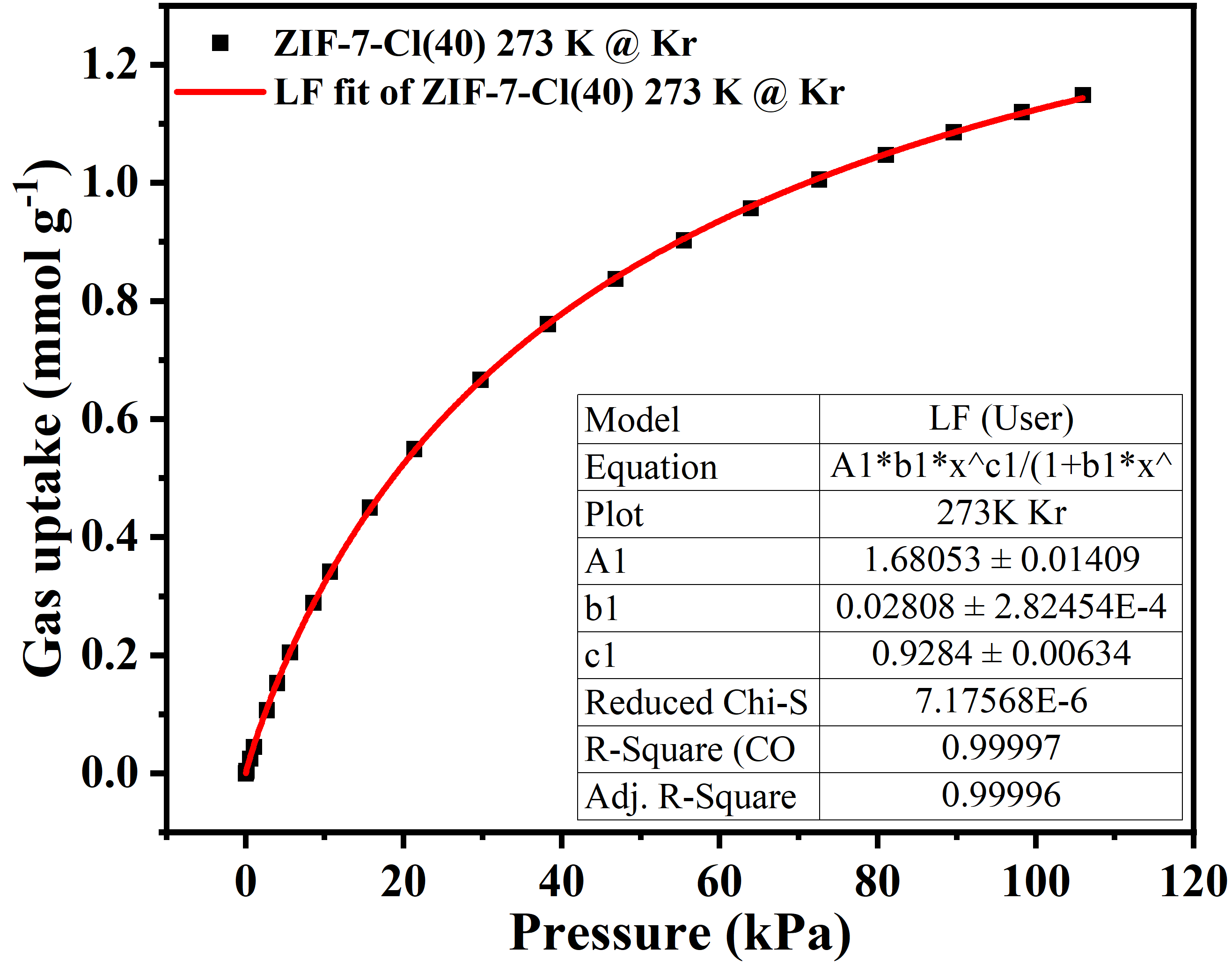


**Figure S36.** The Langmuir-Freundlich fitting results of Kr isotherm at 273 K in ZIF-7-Cl(40).


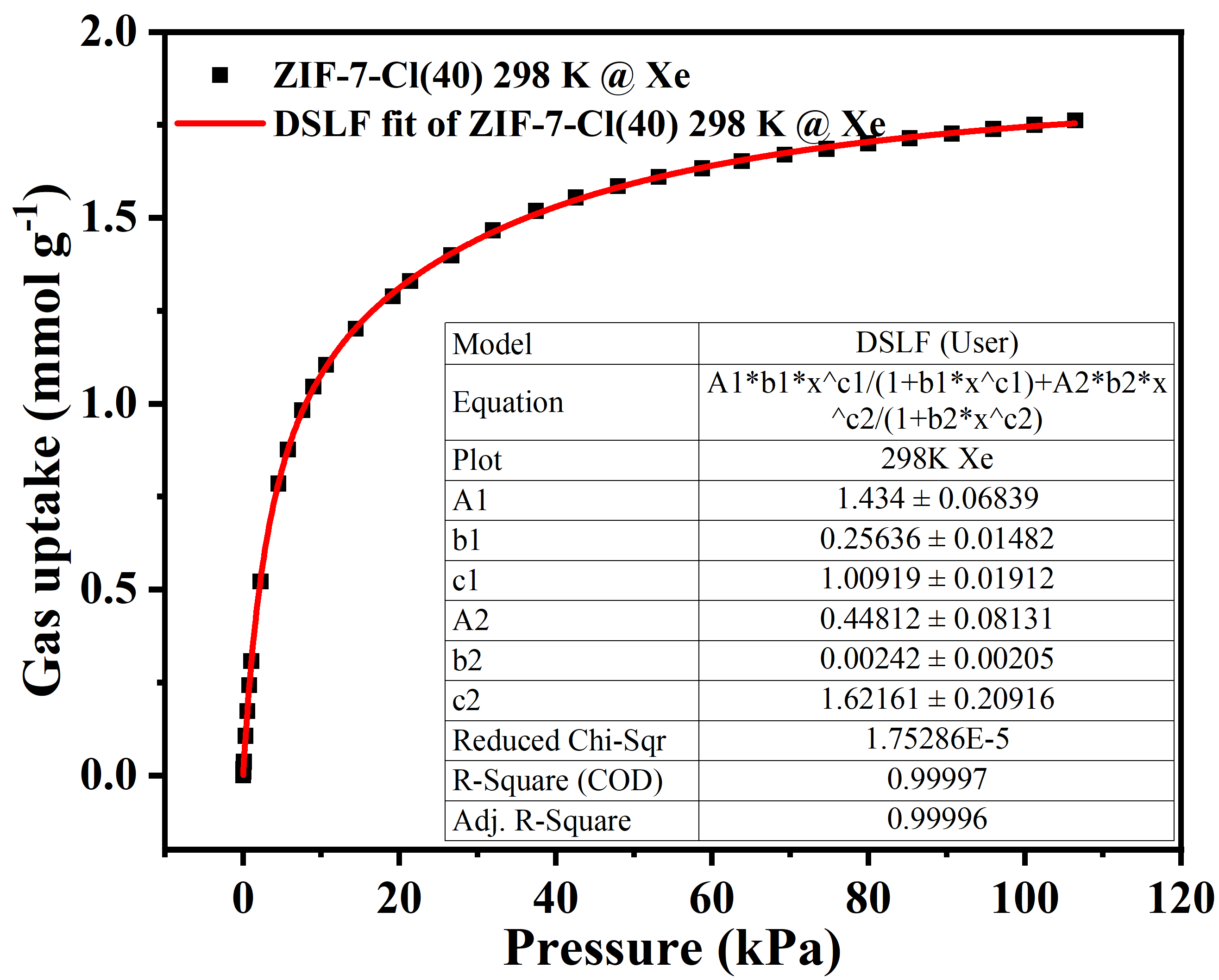


**Figure S37.** The Langmuir-Freundlich fitting results of Xe isotherm at 298 K in ZIF-7-Cl(40).


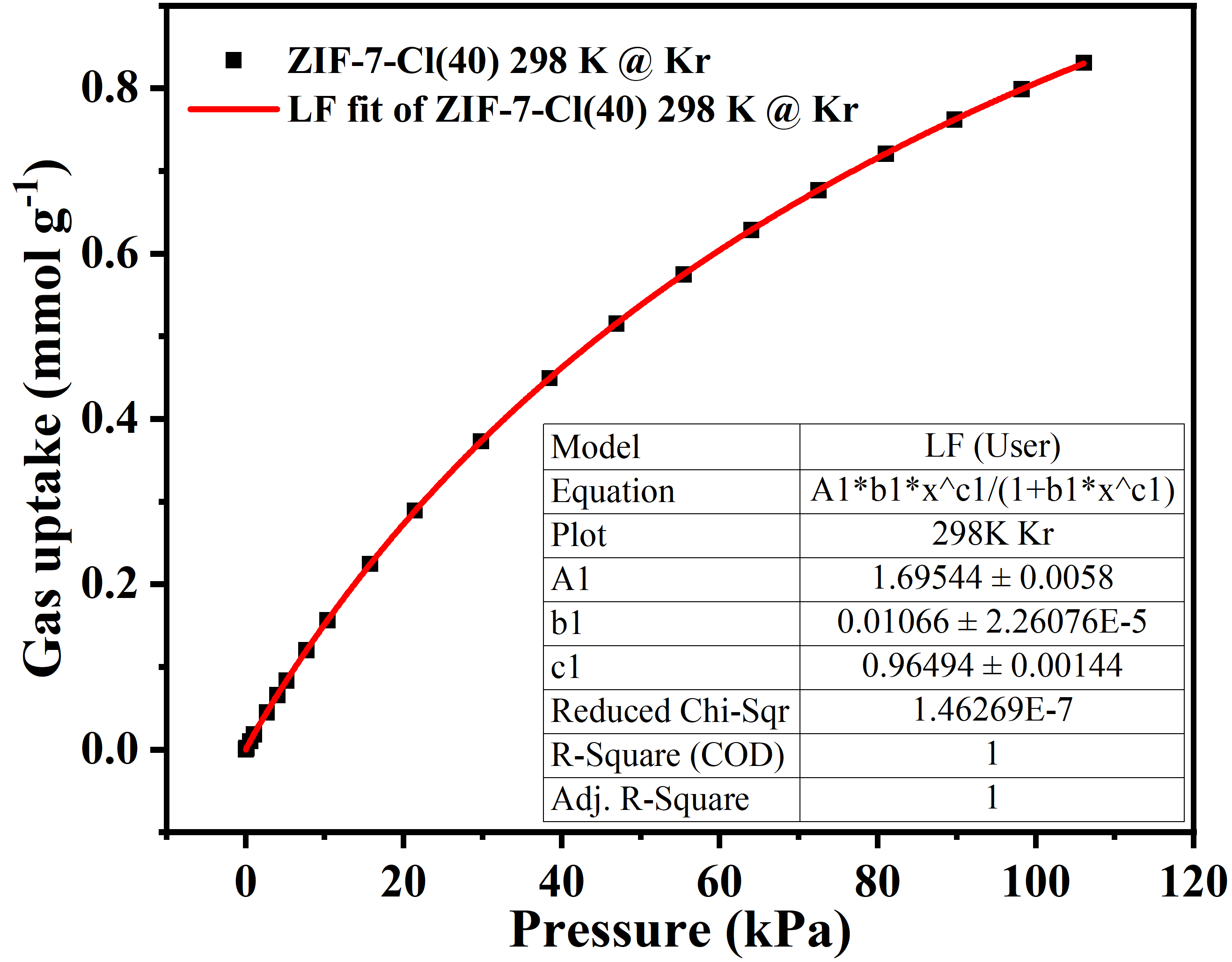


**Figure S38.** The Langmuir-Freundlich fitting results of Kr isotherm at 298 K in ZIF-7-Cl(40).


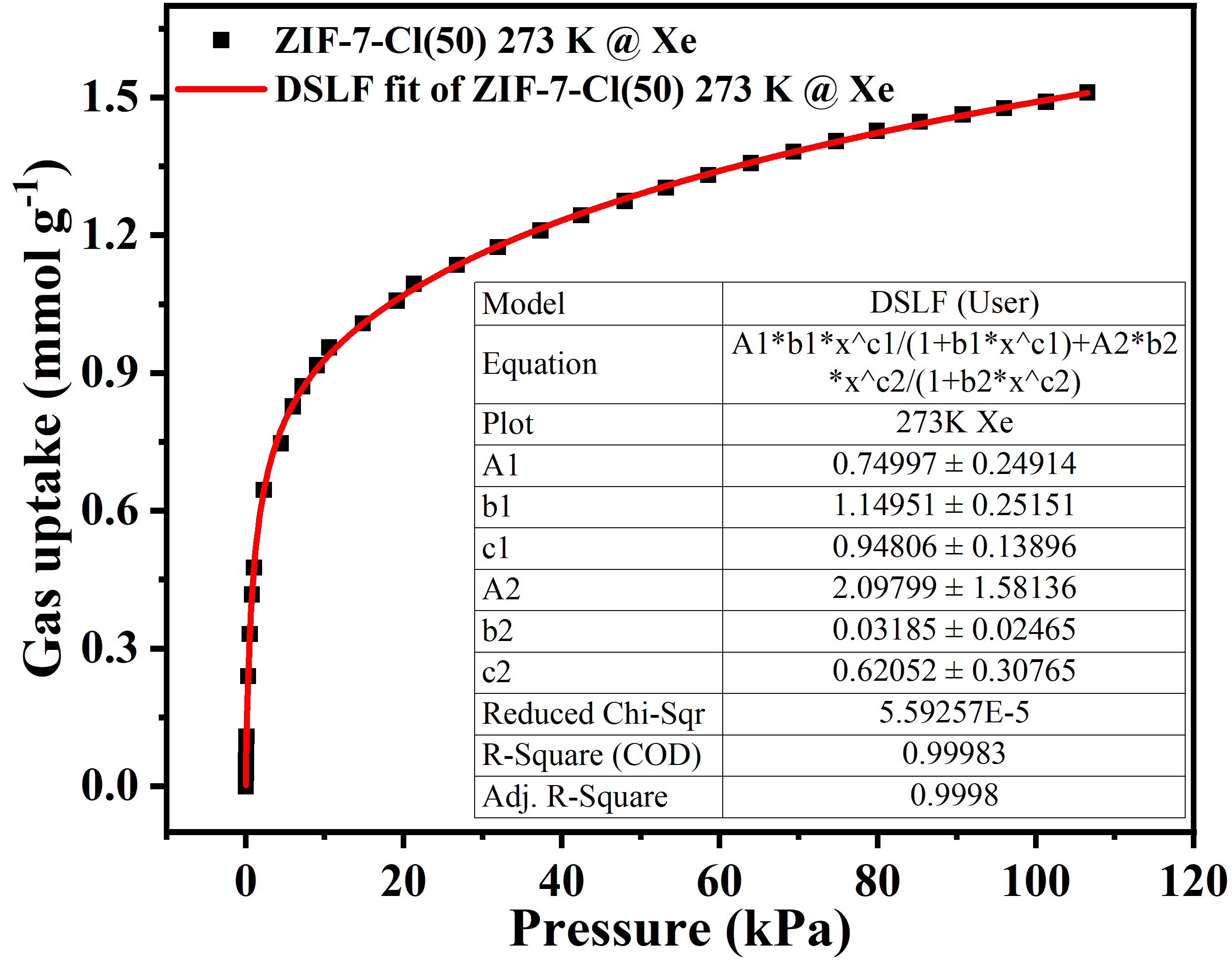


**Figure S39.** The Langmuir-Freundlich fitting results of Xe isotherm at 273 K in ZIF-7-Cl(50).


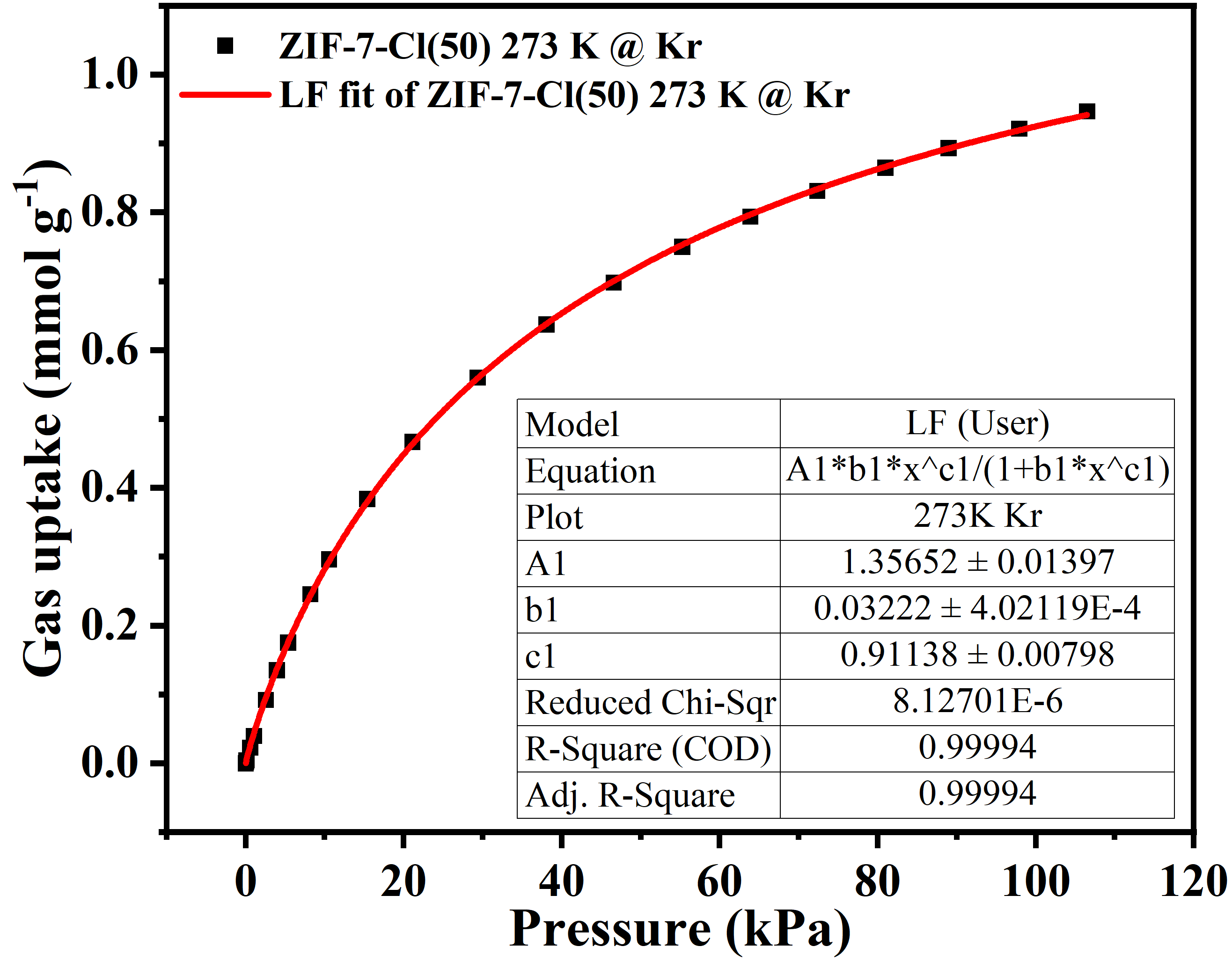


**Figure S40.** The Langmuir-Freundlich fitting results of Kr isotherm at 273 K in ZIF-7-Cl(50).


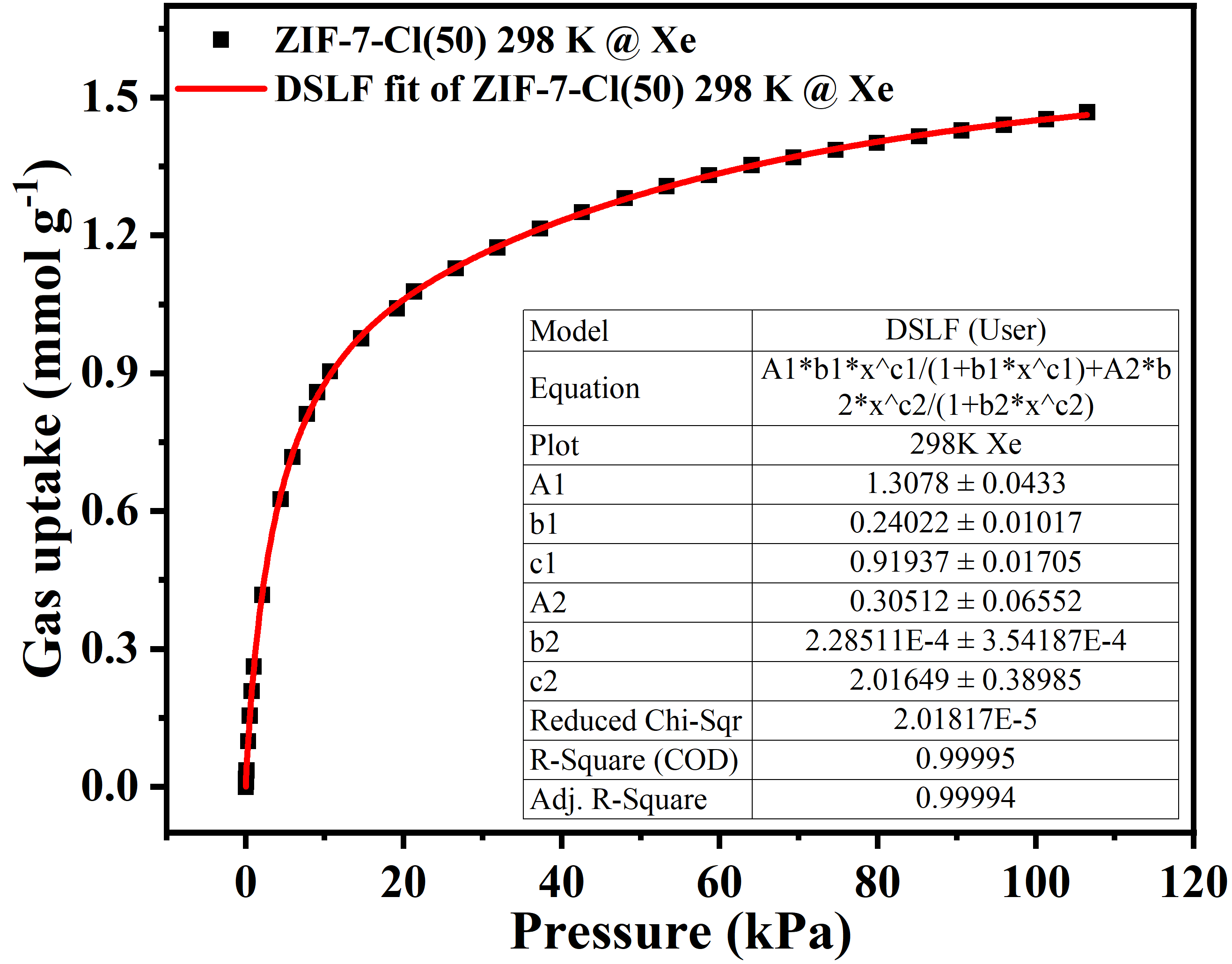


**Figure S41.** The Langmuir-Freundlich fitting results of Xe isotherm at 298 K in ZIF-7-Cl(50).


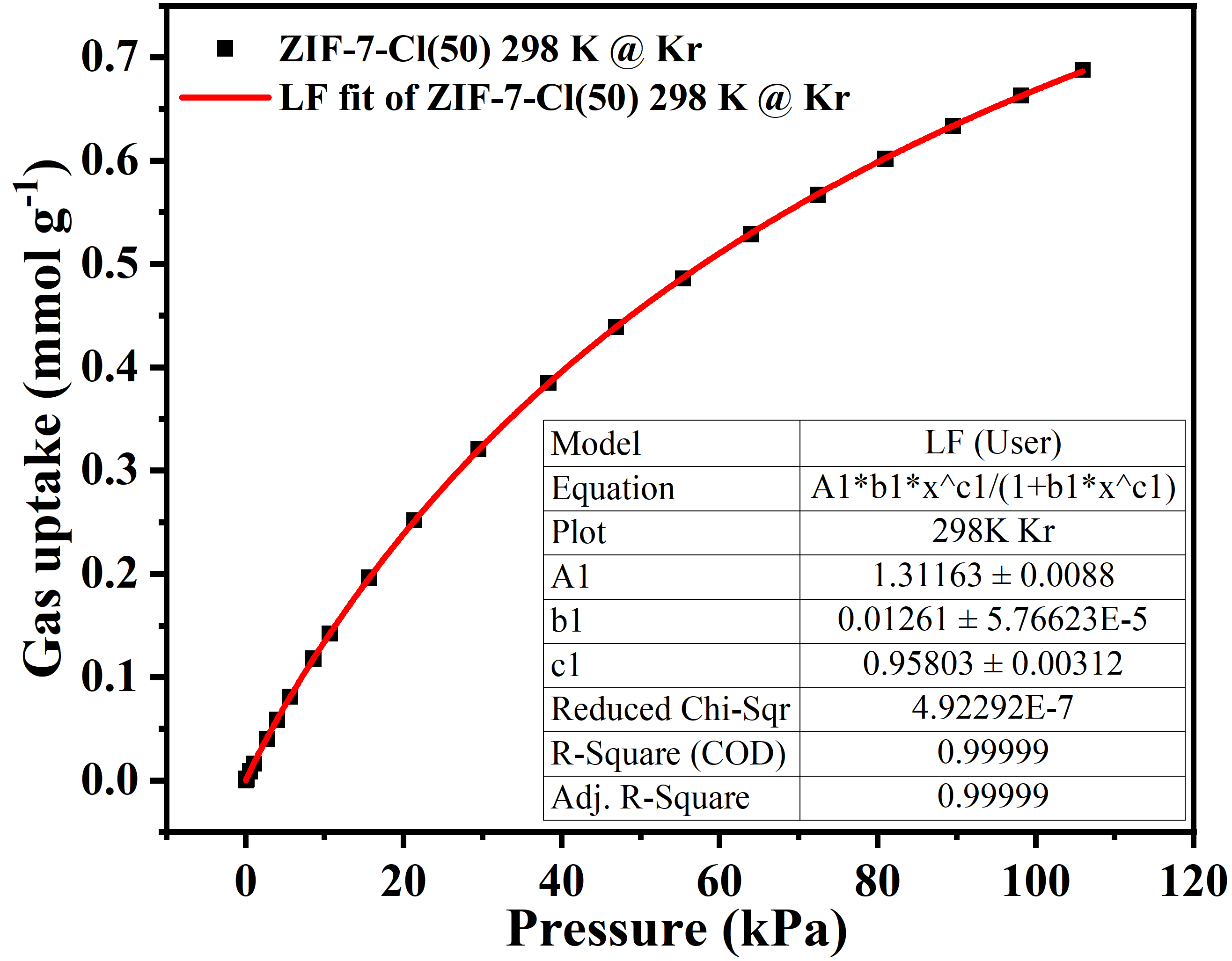


**Figure S42.** The Langmuir-Freundlich fitting results of Kr isotherm at 298 K in ZIF-7-Cl(50).


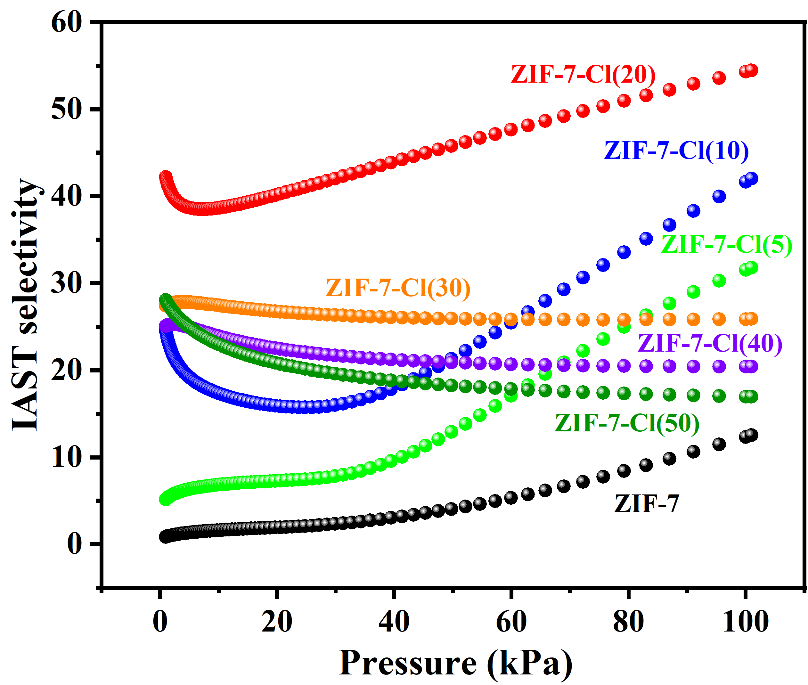


**Figure S43.** IAST selectivity of ZIF-7 and ZIF-7-Cl(x) for Xe/Kr (20/80, v/v) at 273 K.


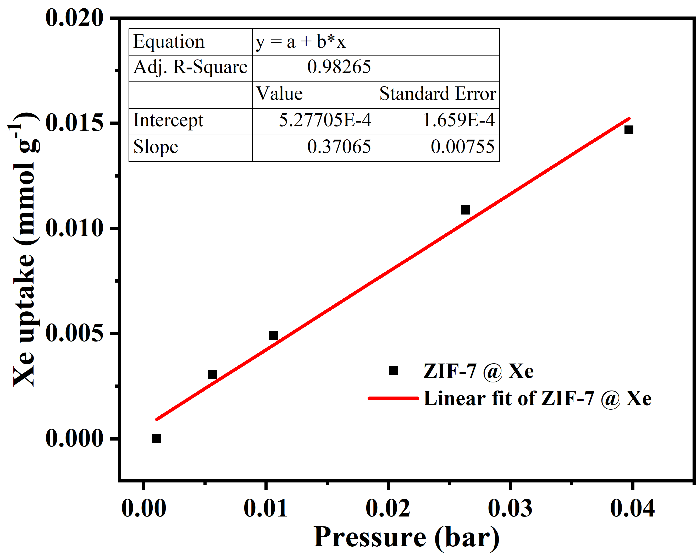


**Figure S44.** Henry coefficient fitting of Xe adsorption isotherm for ZIF-7 at 298 K.


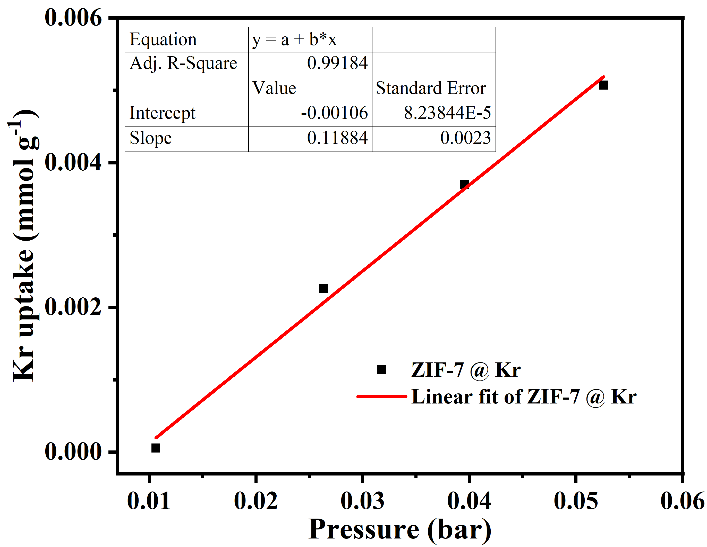


**Figure S45.** Henry coefficient fitting of Kr adsorption isotherm for ZIF-7 at 298 K.


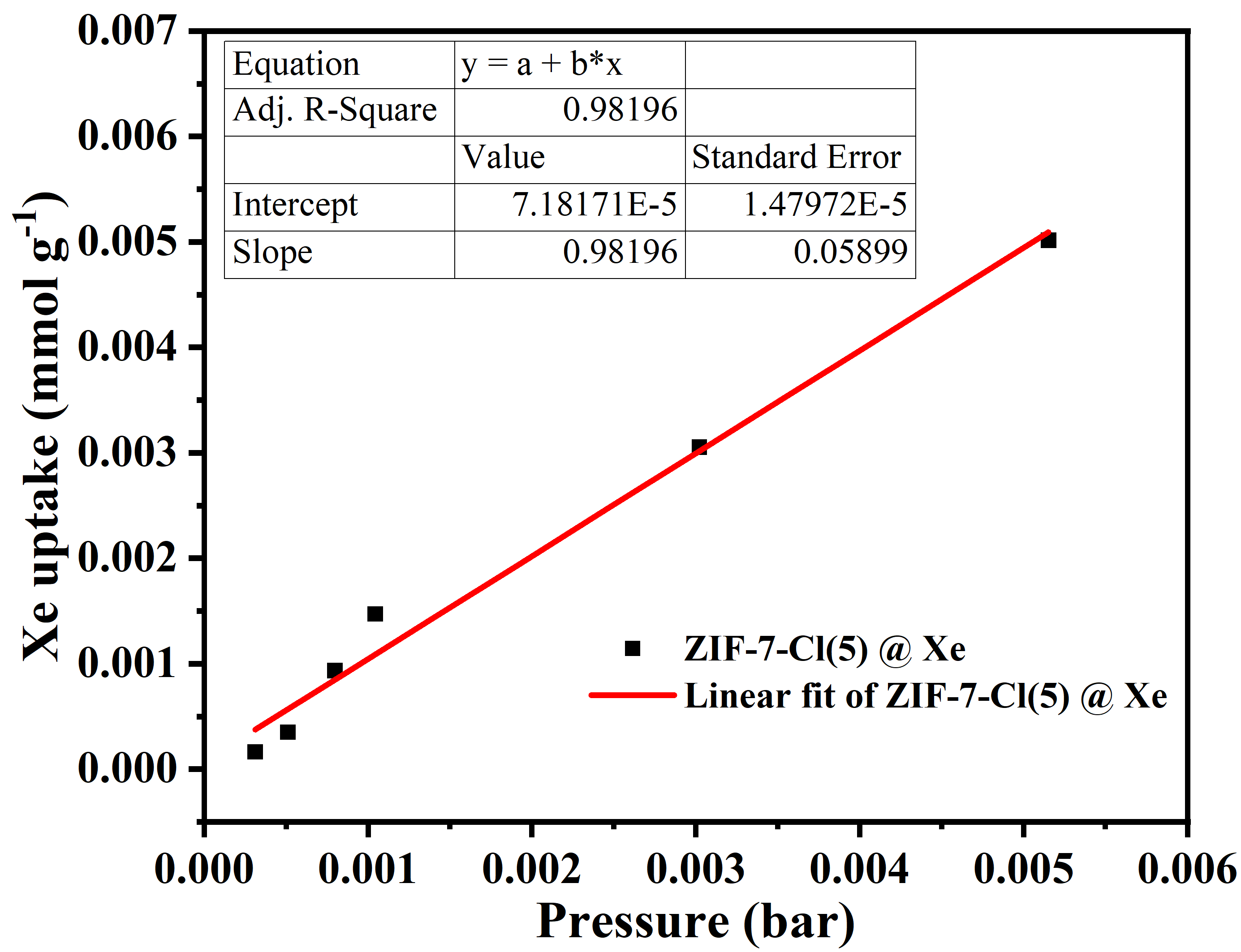


**Figure S46.** Henry coefficient fitting of Xe adsorption isotherm for ZIF-7-Cl(5) at 298 K.


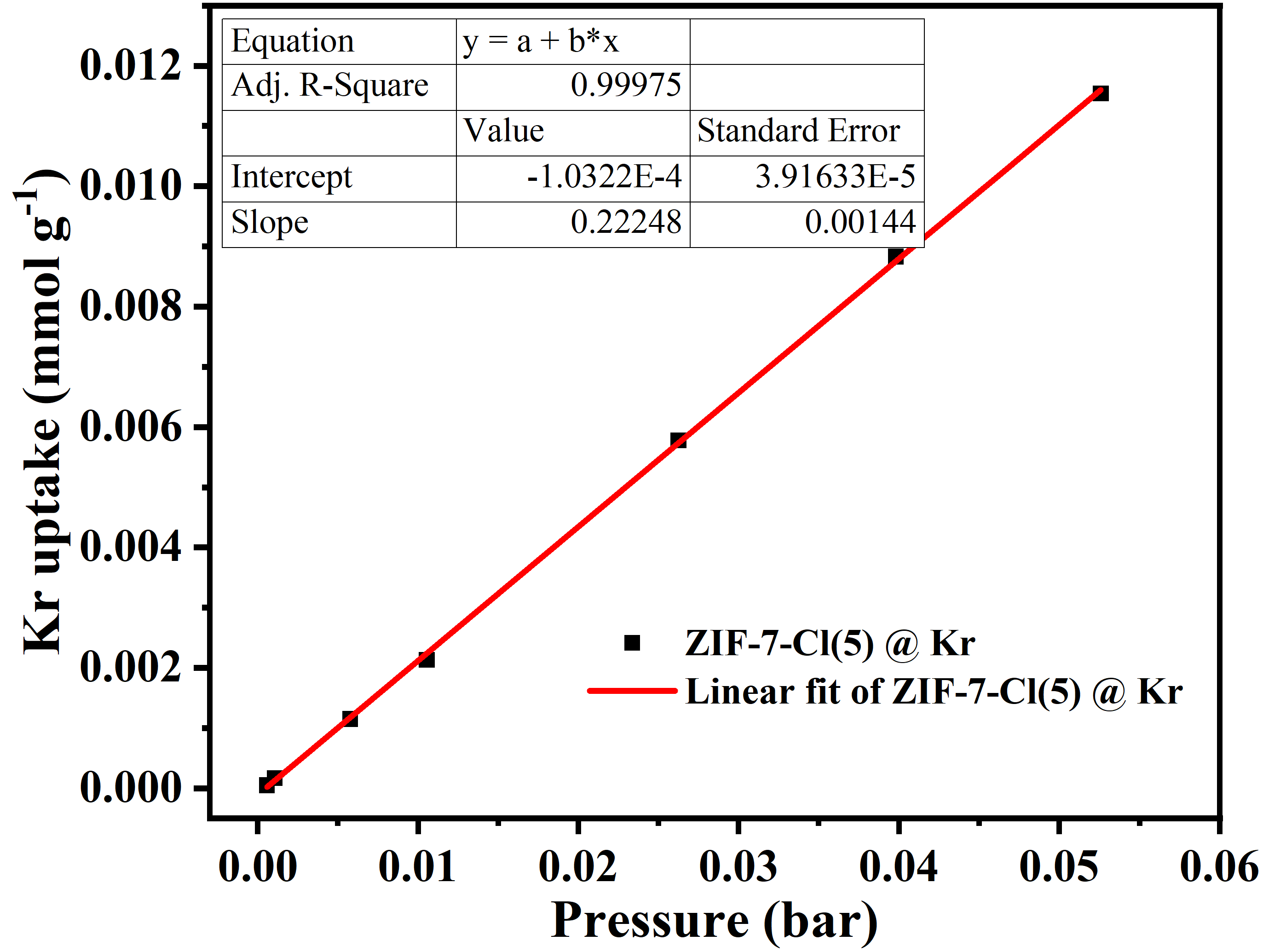


**Figure S47.** Henry coefficient fitting of Kr adsorption isotherm for ZIF-7-Cl(5) at 298 K.


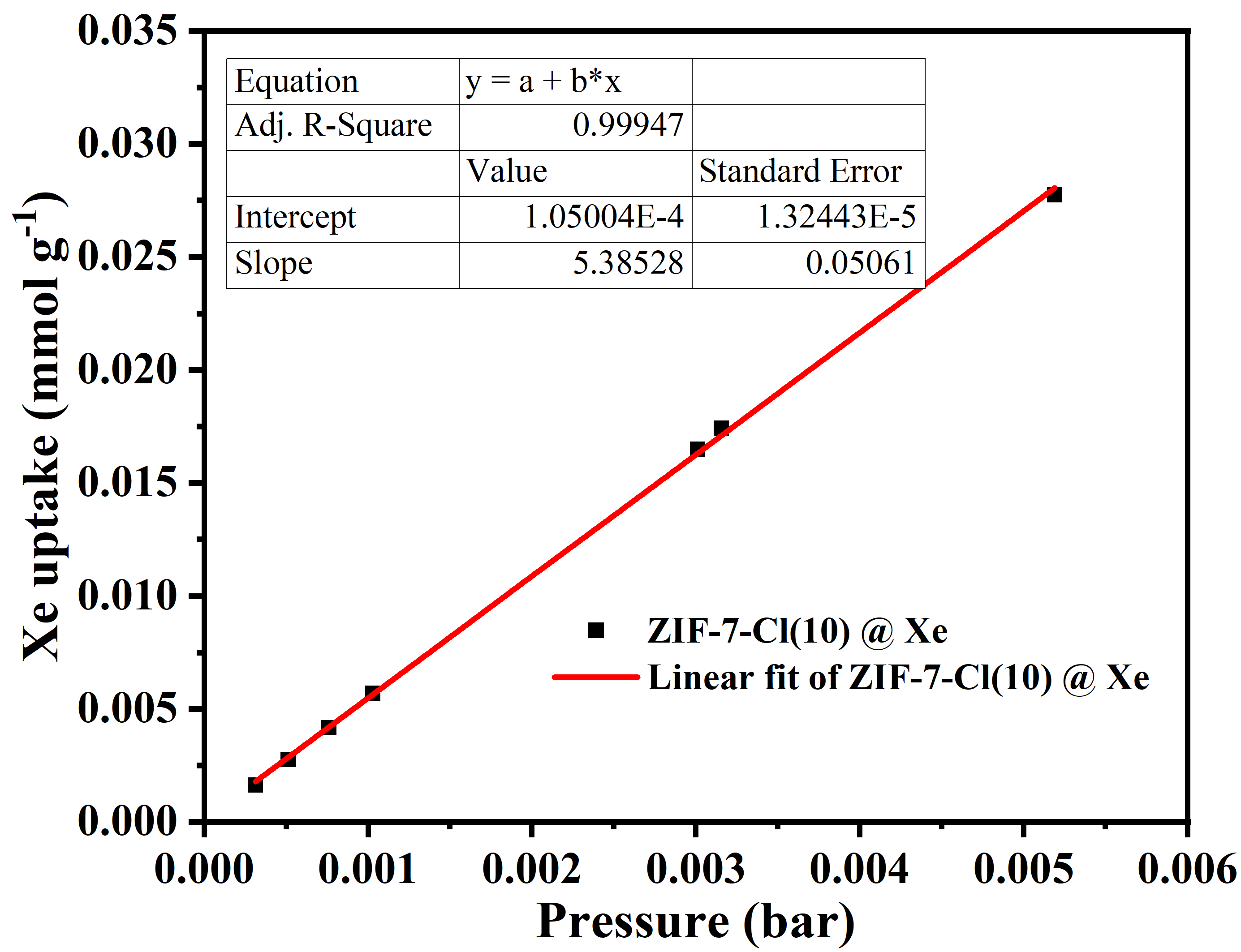


**Figure S48.** Henry coefficient fitting of Xe adsorption isotherm for ZIF-7-Cl(10) at 298 K.


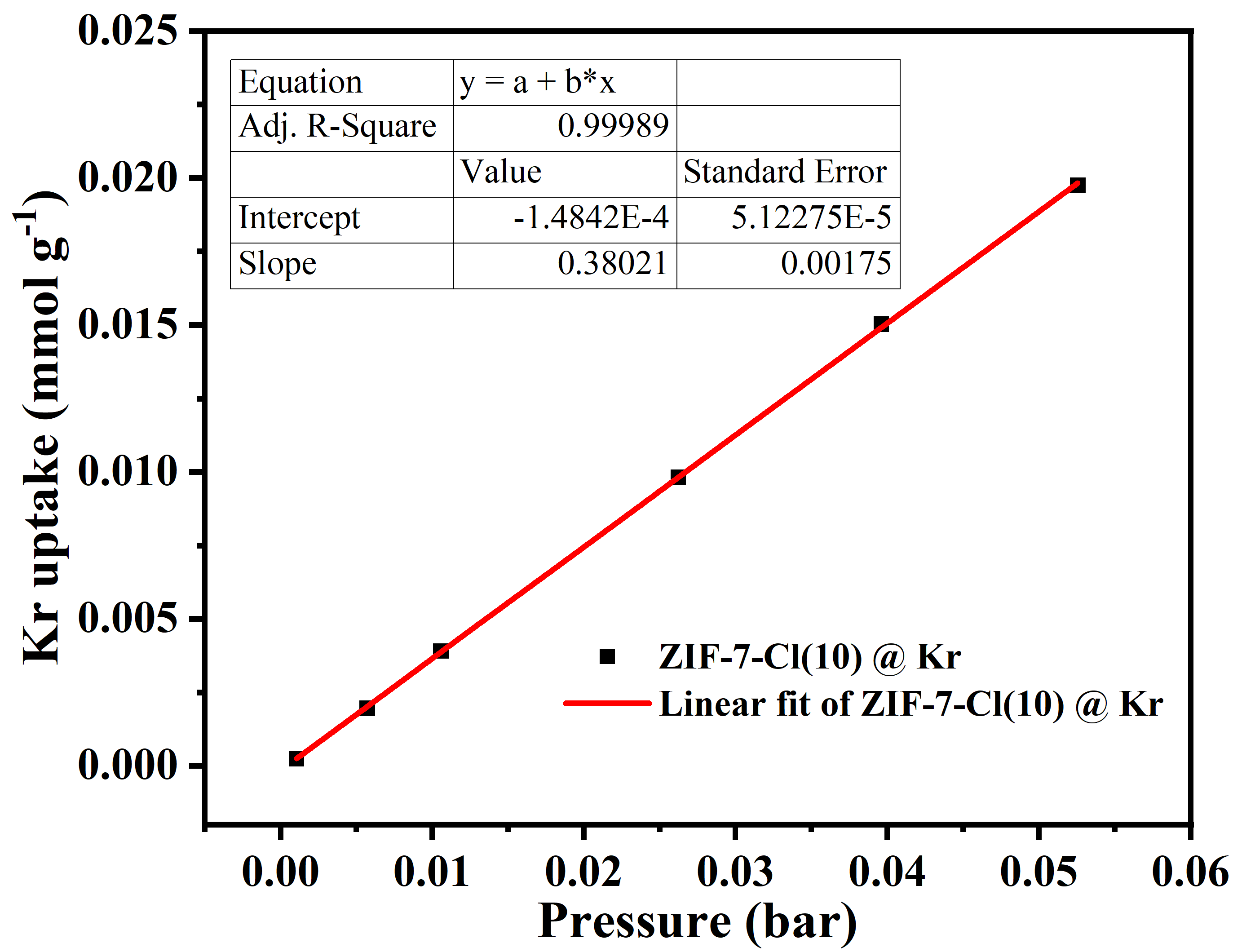


**Figure S49.** Henry coefficient fitting of Kr adsorption isotherm for ZIF-7-Cl(10) at 298 K.


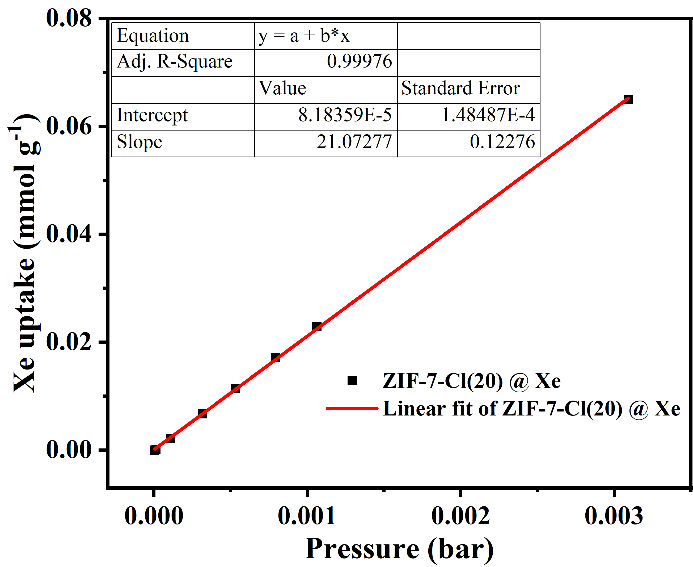


**Figure S50.** Henry coefficient fitting of Xe adsorption isotherm for ZIF-7-Cl(20) at 298 K.


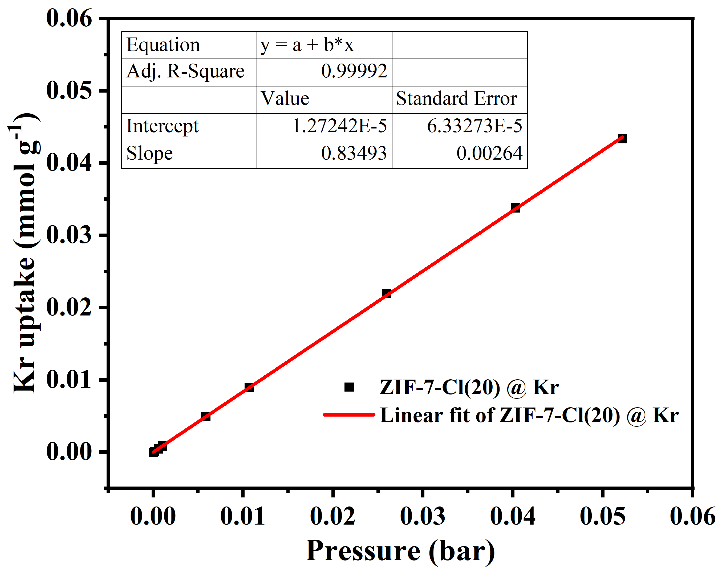


**Figure S51.** Henry coefficient fitting of Kr adsorption isotherm for ZIF-7-Cl(20) at 298 K.


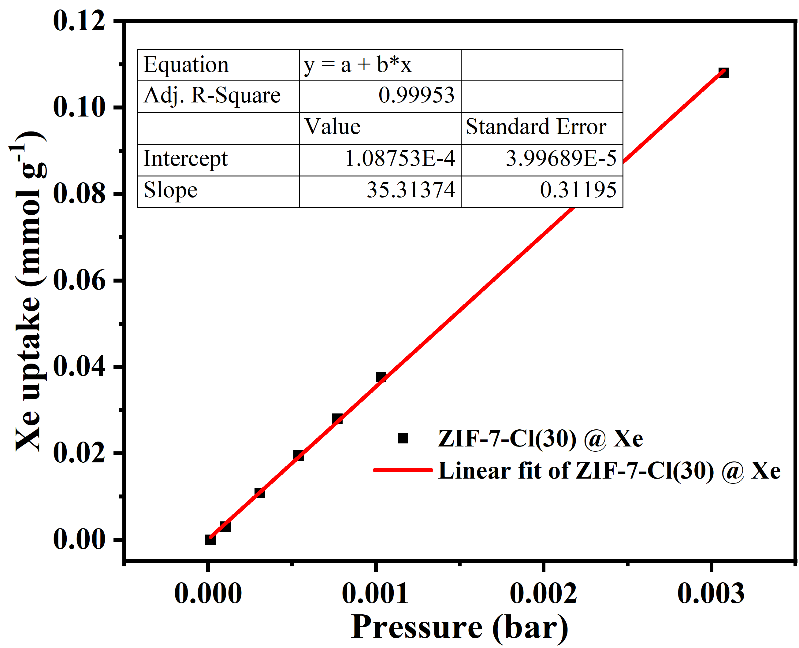


**Figure S52.** Henry coefficient fitting of Xe adsorption isotherm for ZIF-7-Cl(30) at 298 K.


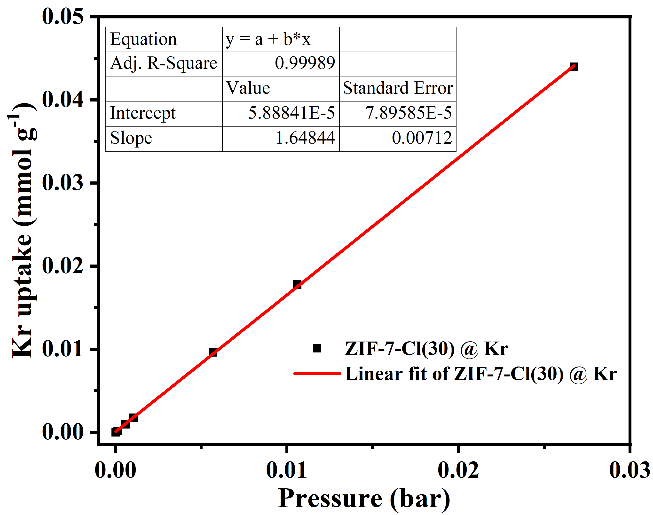


**Figure S53.** Henry coefficient fitting of Kr adsorption isotherm for ZIF-7-Cl(30) at 298 K.


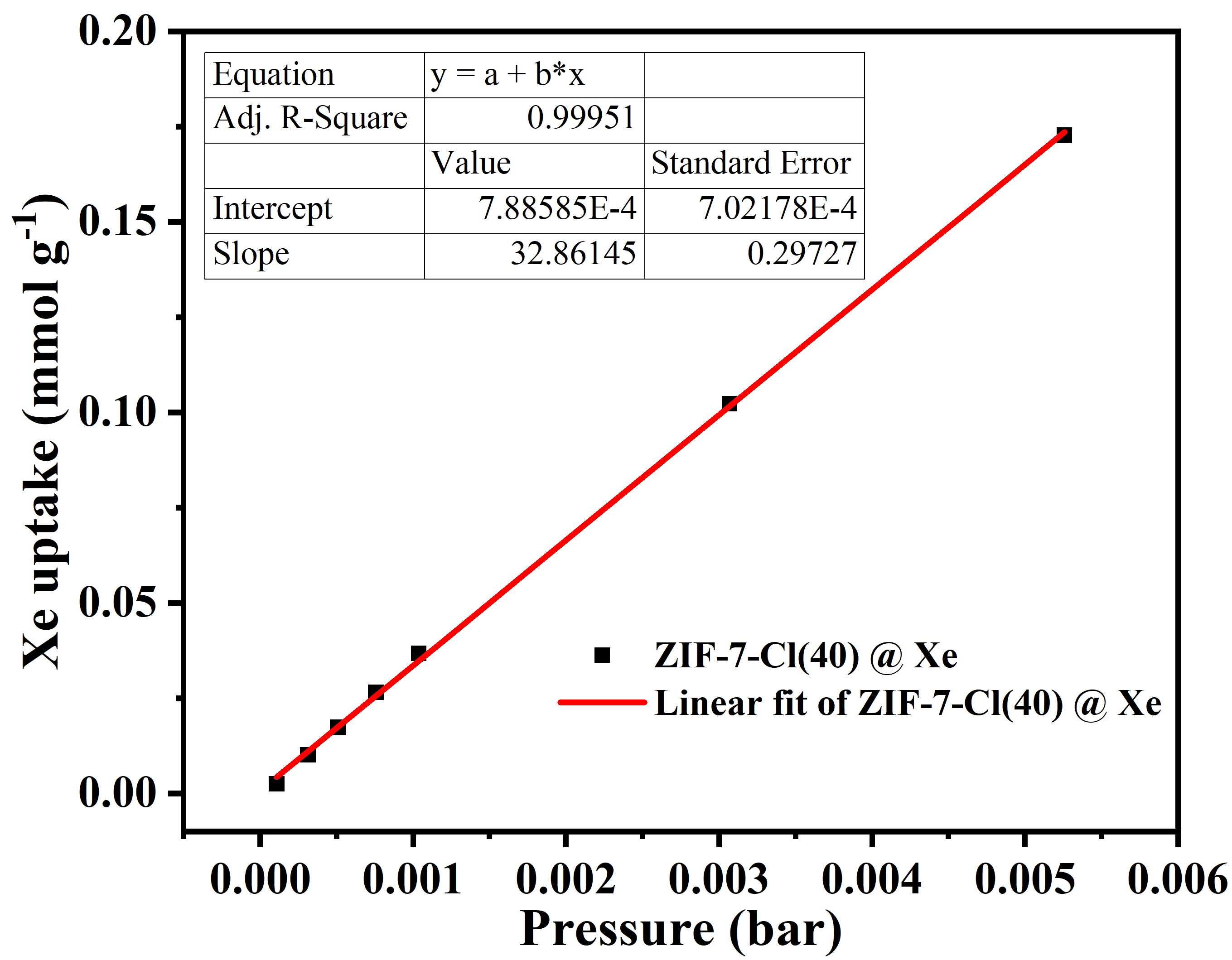


**Figure S54.** Henry coefficient fitting of Xe adsorption isotherm for ZIF-7-Cl(40) at 298 K.


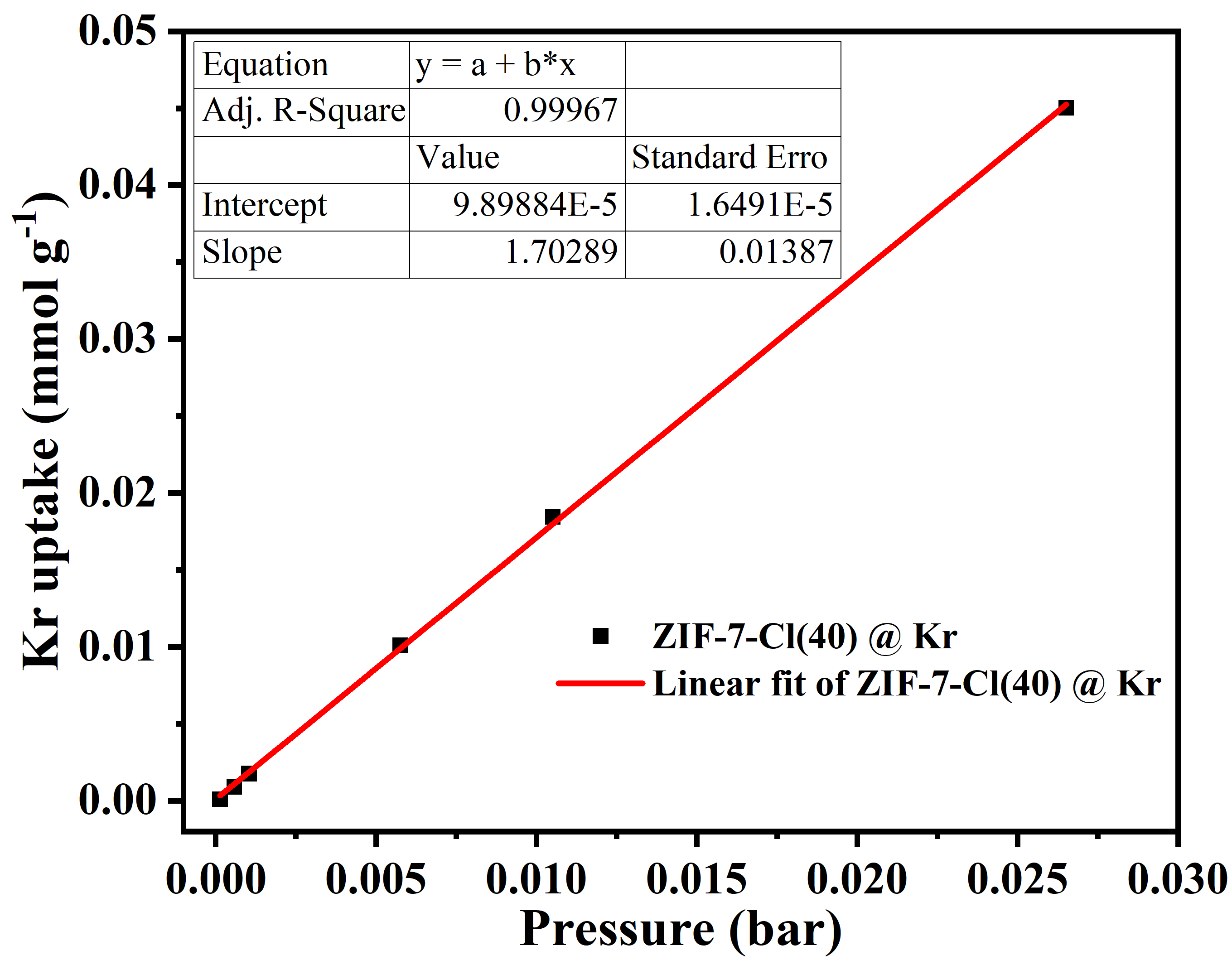


**Figure S55.** Henry coefficient fitting of Kr adsorption isotherm for ZIF-7-Cl(40) at 298 K.


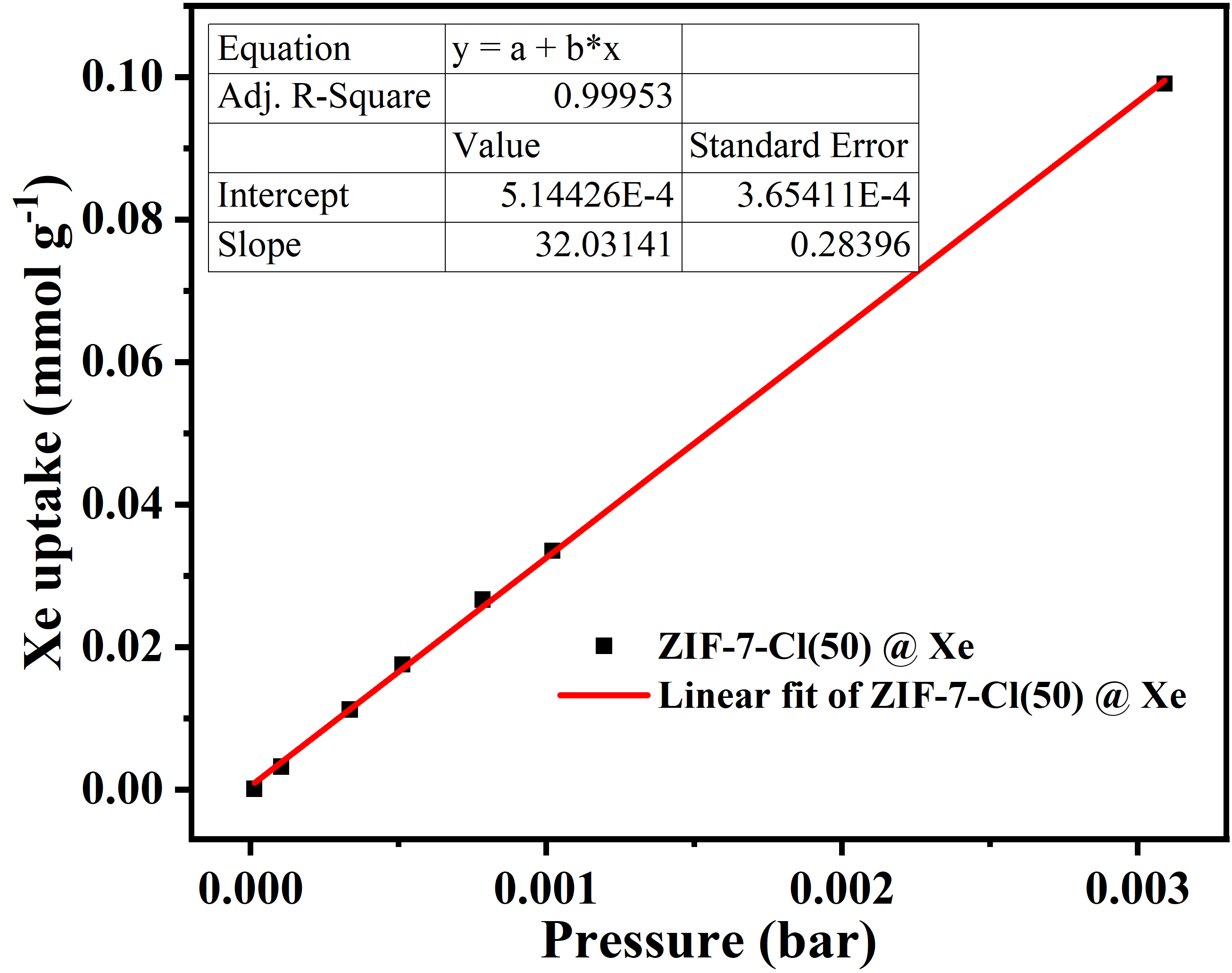


**Figure S56.** Henry coefficient fitting of Xe adsorption isotherm for ZIF-7-Cl(50) at 298 K.


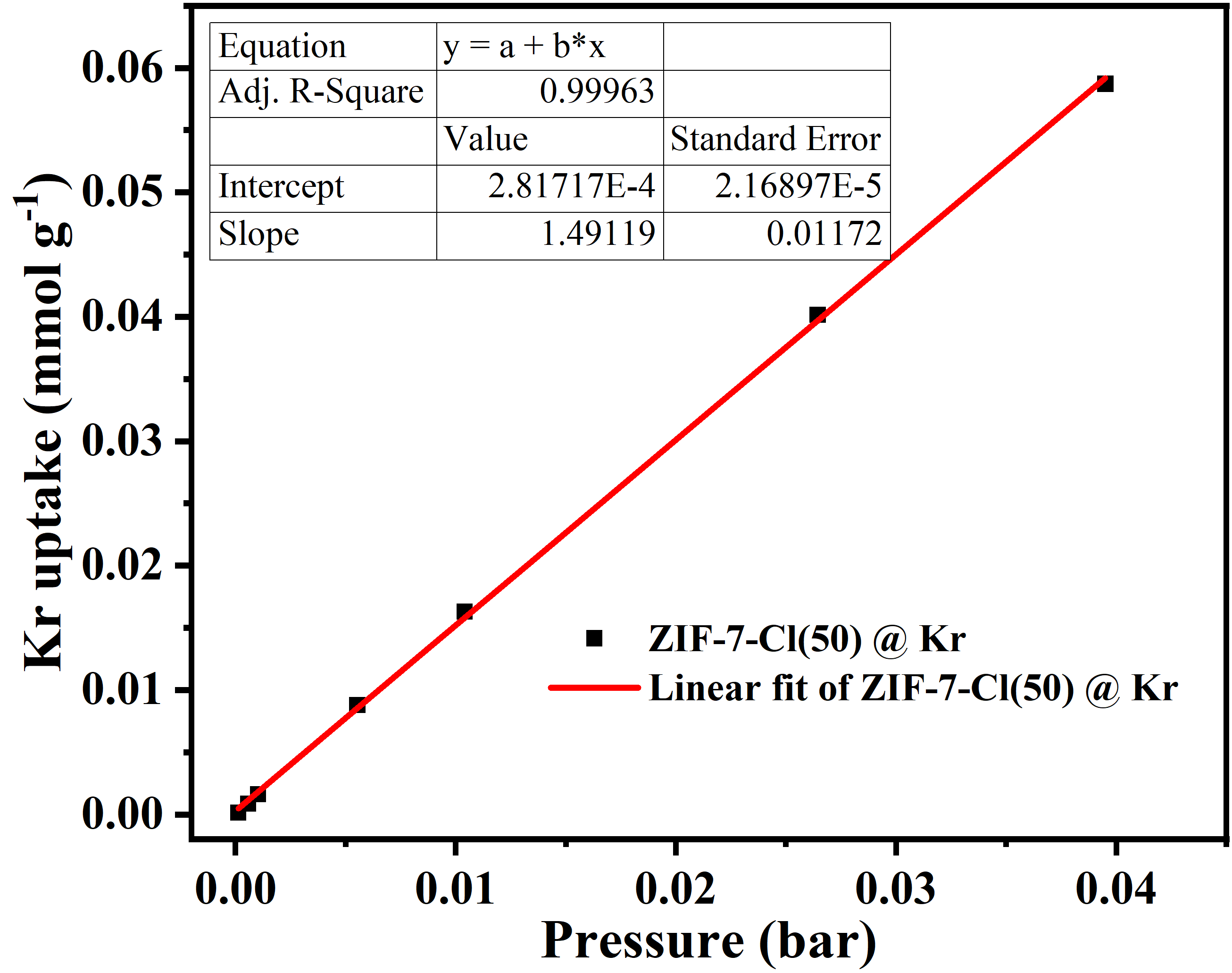


**Figure S57.** Henry coefficient fitting of Kr adsorption isotherm for ZIF-7-Cl(50) at 298 K.


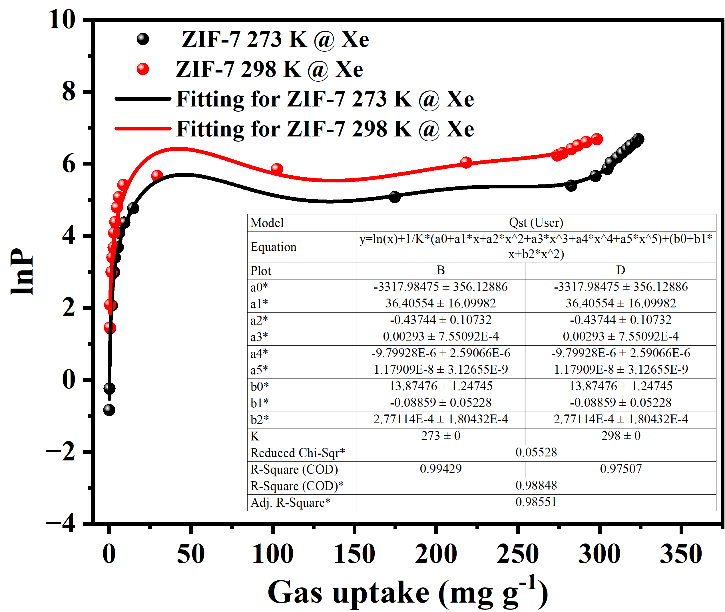


**Figure S58.** The virial fitting of Xe adsorption isotherm for ZIF-7.


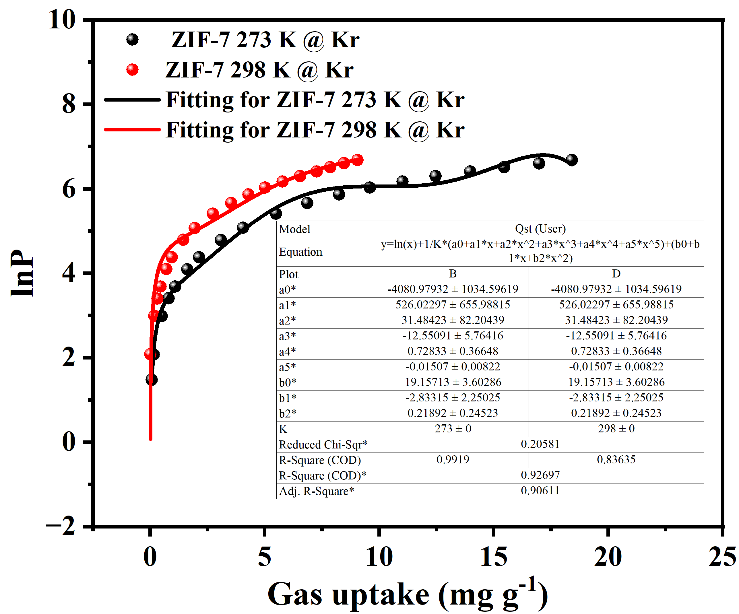


**Figure S59.** The virial fitting of Kr adsorption isotherm for ZIF-7.


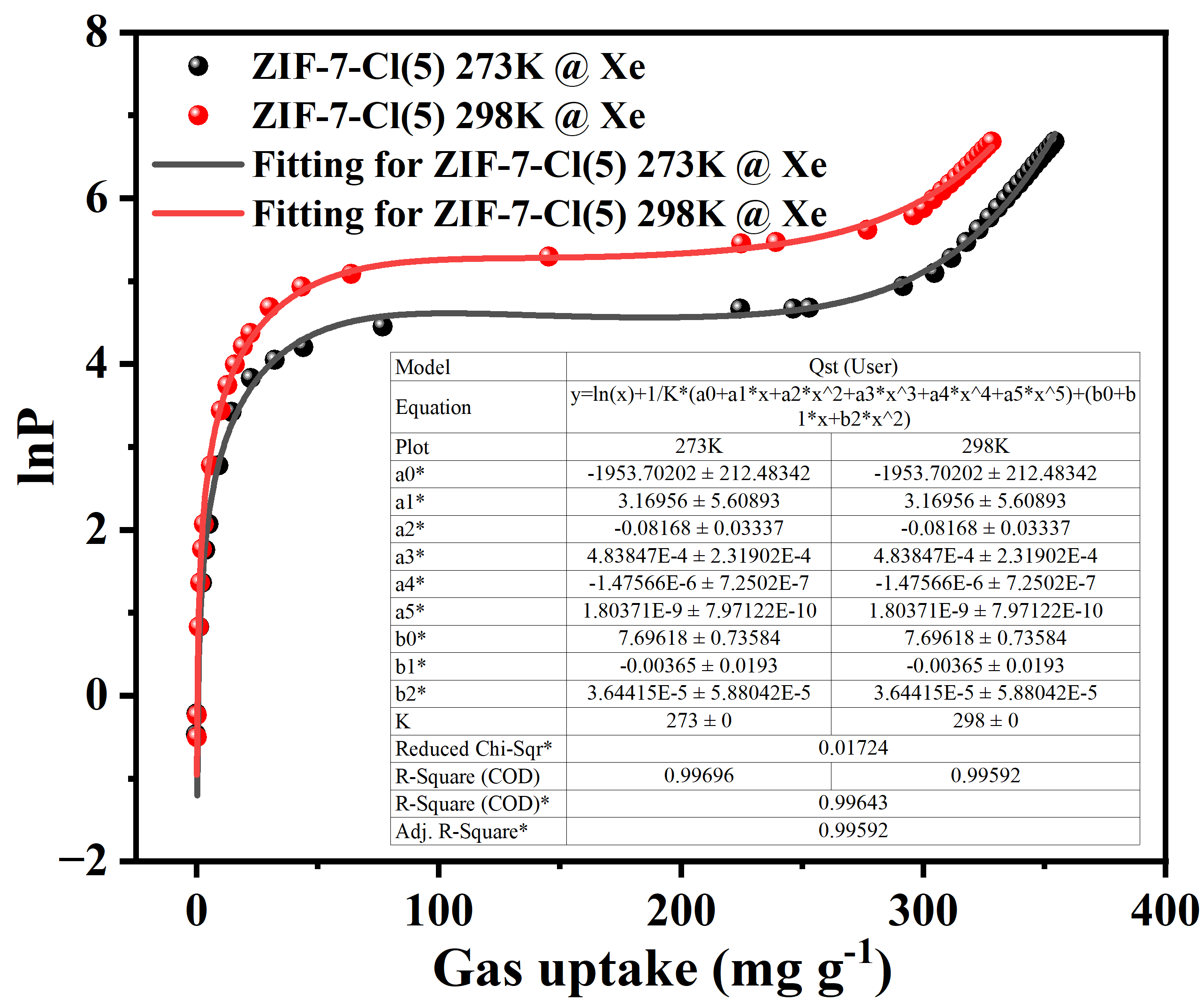


**Figure S60.** The virial fitting of Xe adsorption isotherm for ZIF-7-Cl(5).


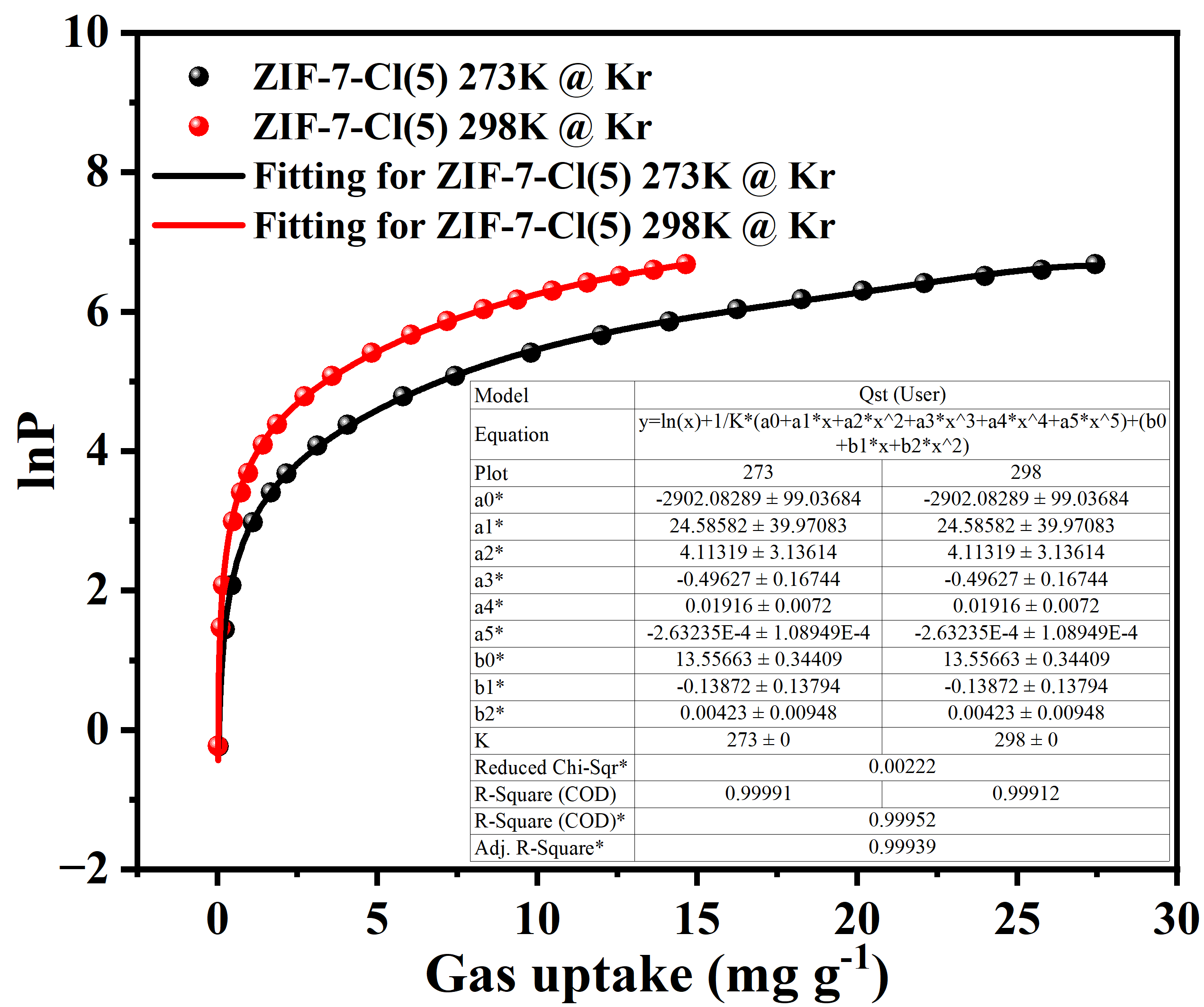


**Figure S61.** The virial fitting of Kr adsorption isotherm for ZIF-7-Cl(5).


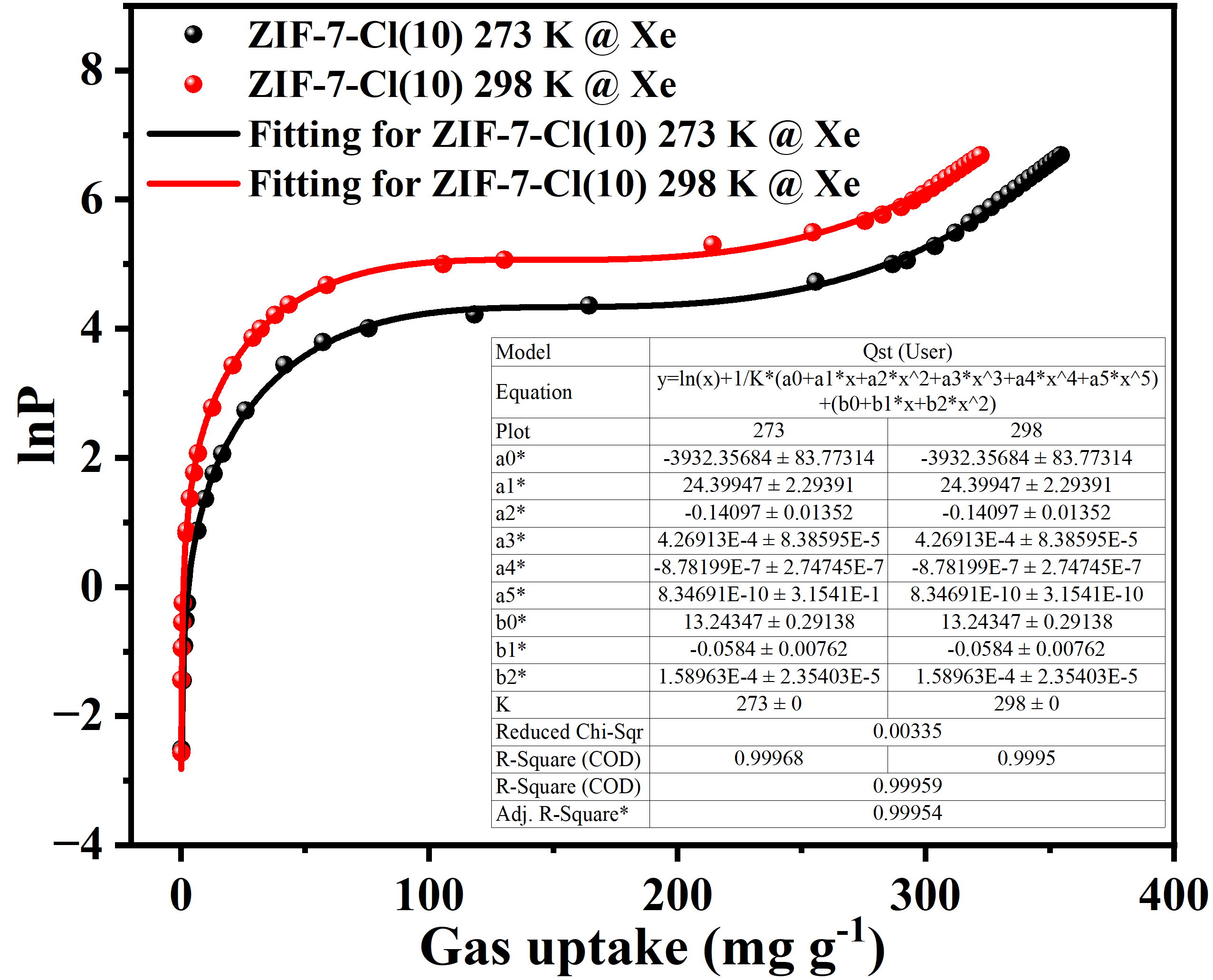


**Figure S62.** The virial fitting of Xe adsorption isotherm for ZIF-7-Cl(10).


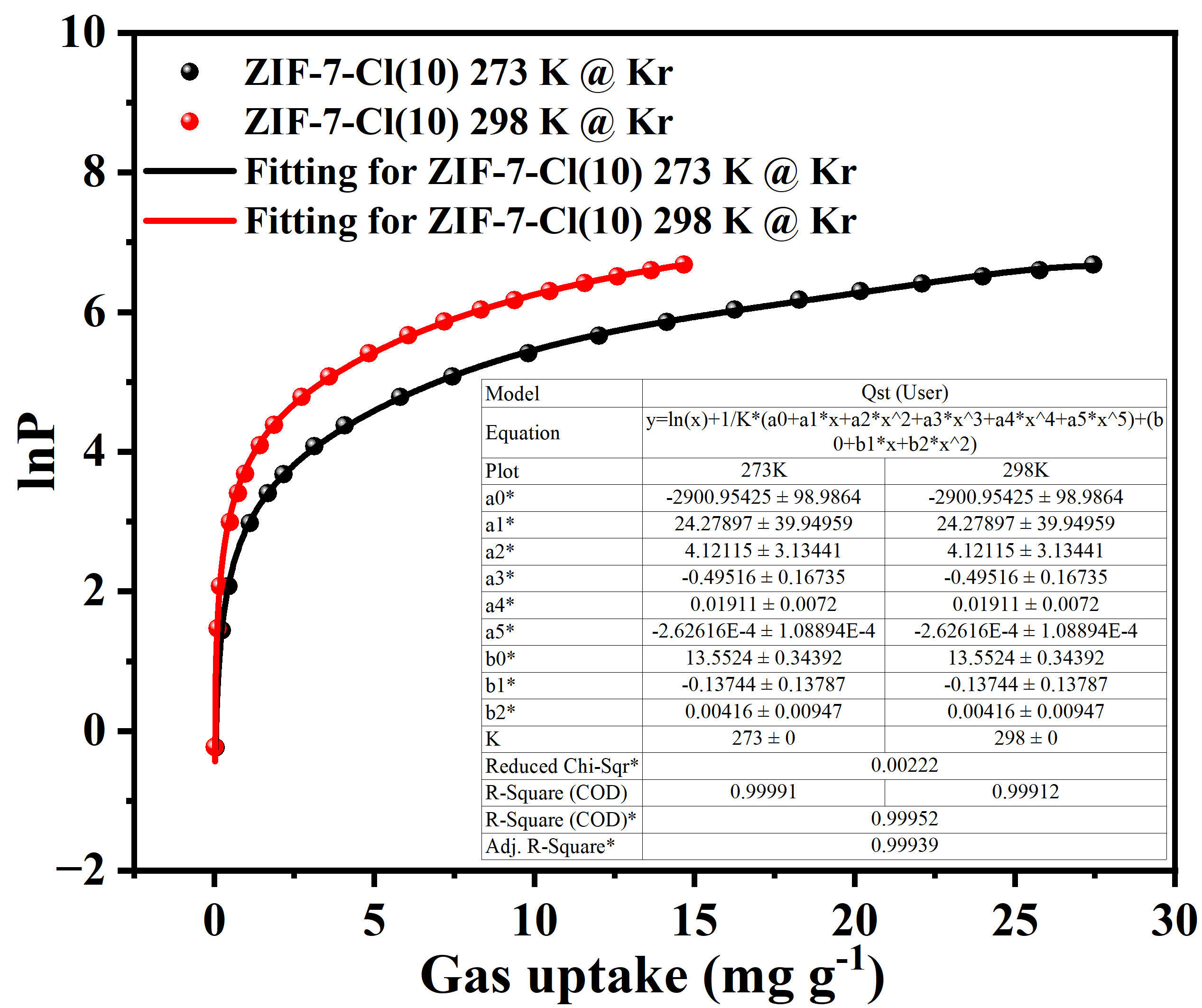


**Figure S63.** The virial fitting of Kr adsorption isotherm for ZIF-7-Cl(10).


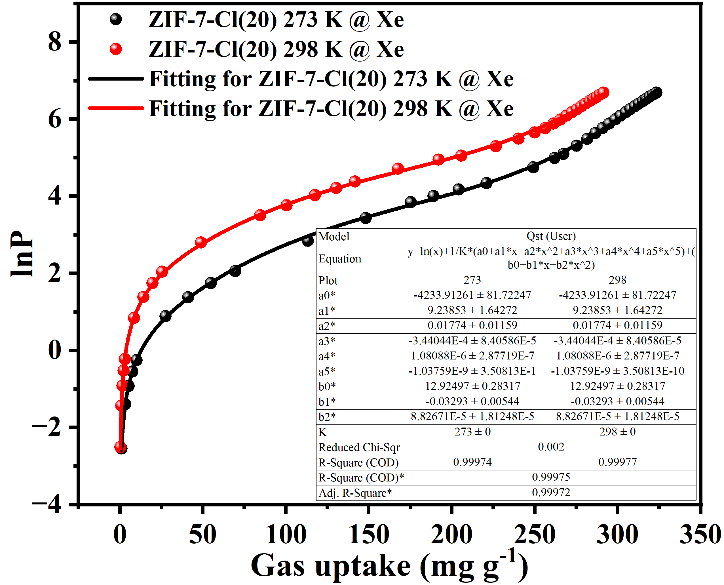


**Figure S64.** The virial fitting of Xe adsorption isotherm for ZIF-7-Cl(20).


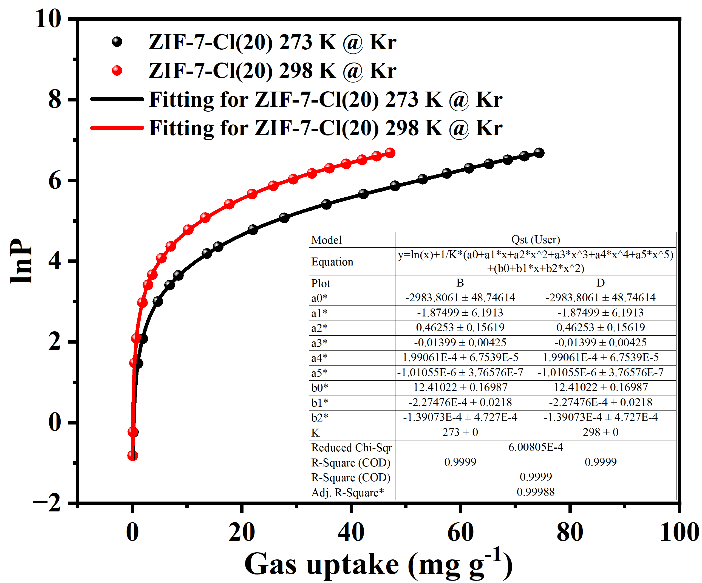


**Figure S65.** The virial fitting of Kr adsorption isotherm for ZIF-7-Cl(20).


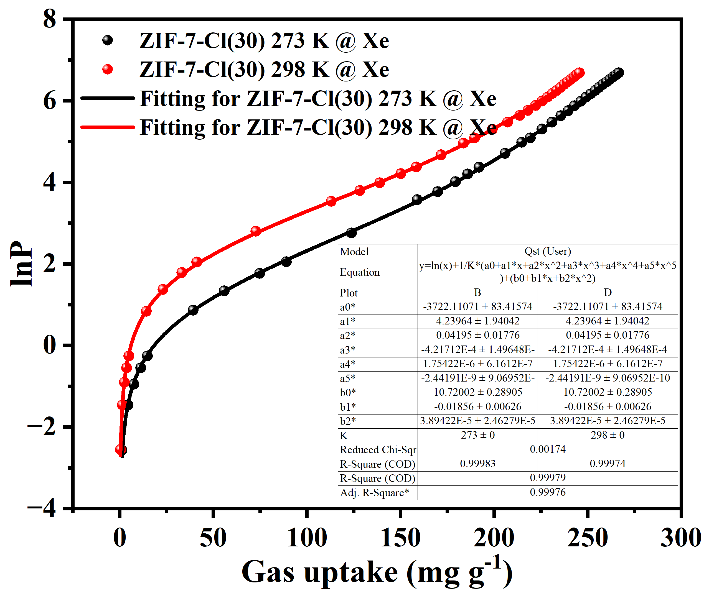


**Figure S66.** The virial fitting of Xe adsorption isotherm for ZIF-7-Cl(30).


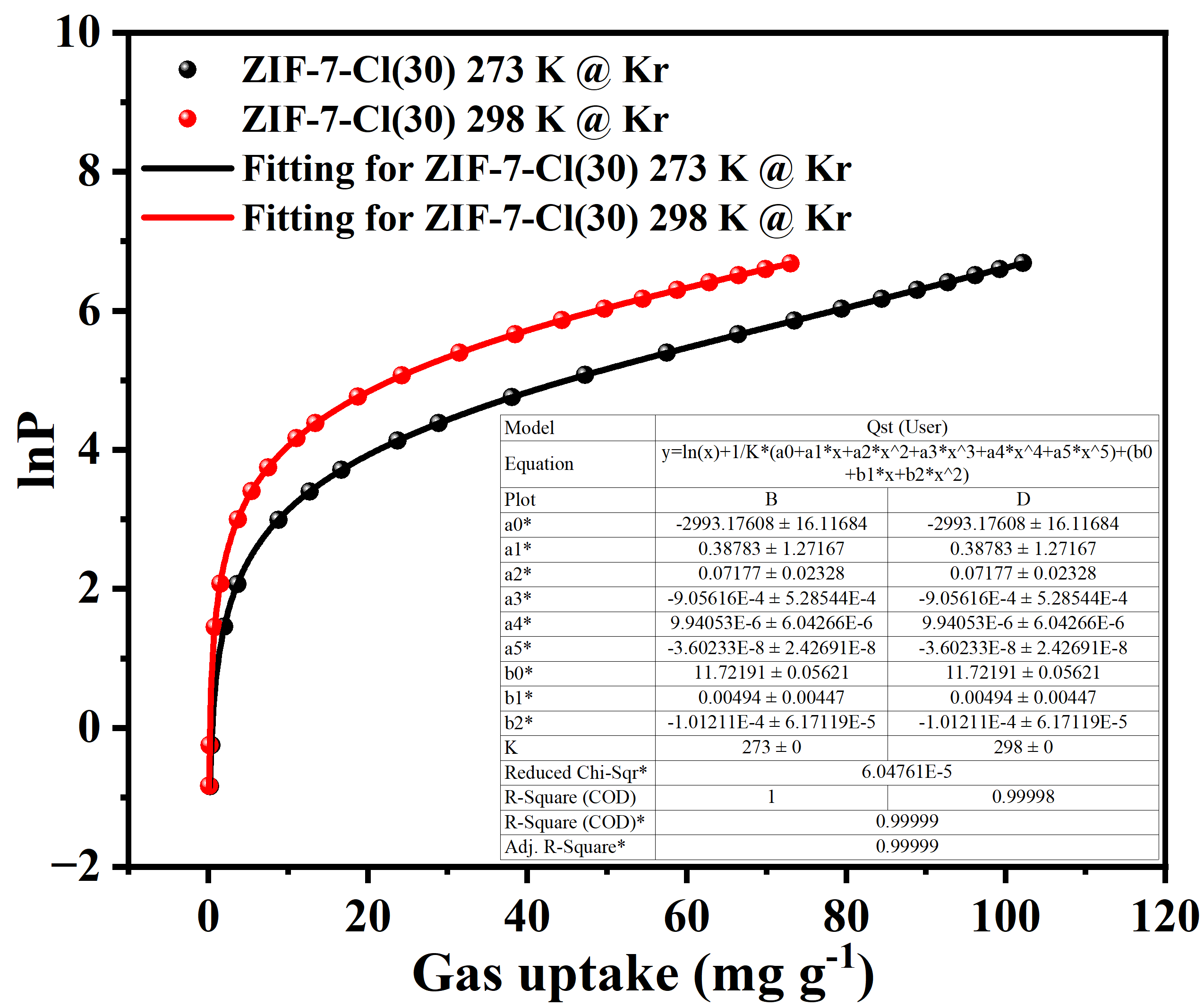


**Figure S67.** The virial fitting of Kr adsorption isotherm for ZIF-7-Cl(30).


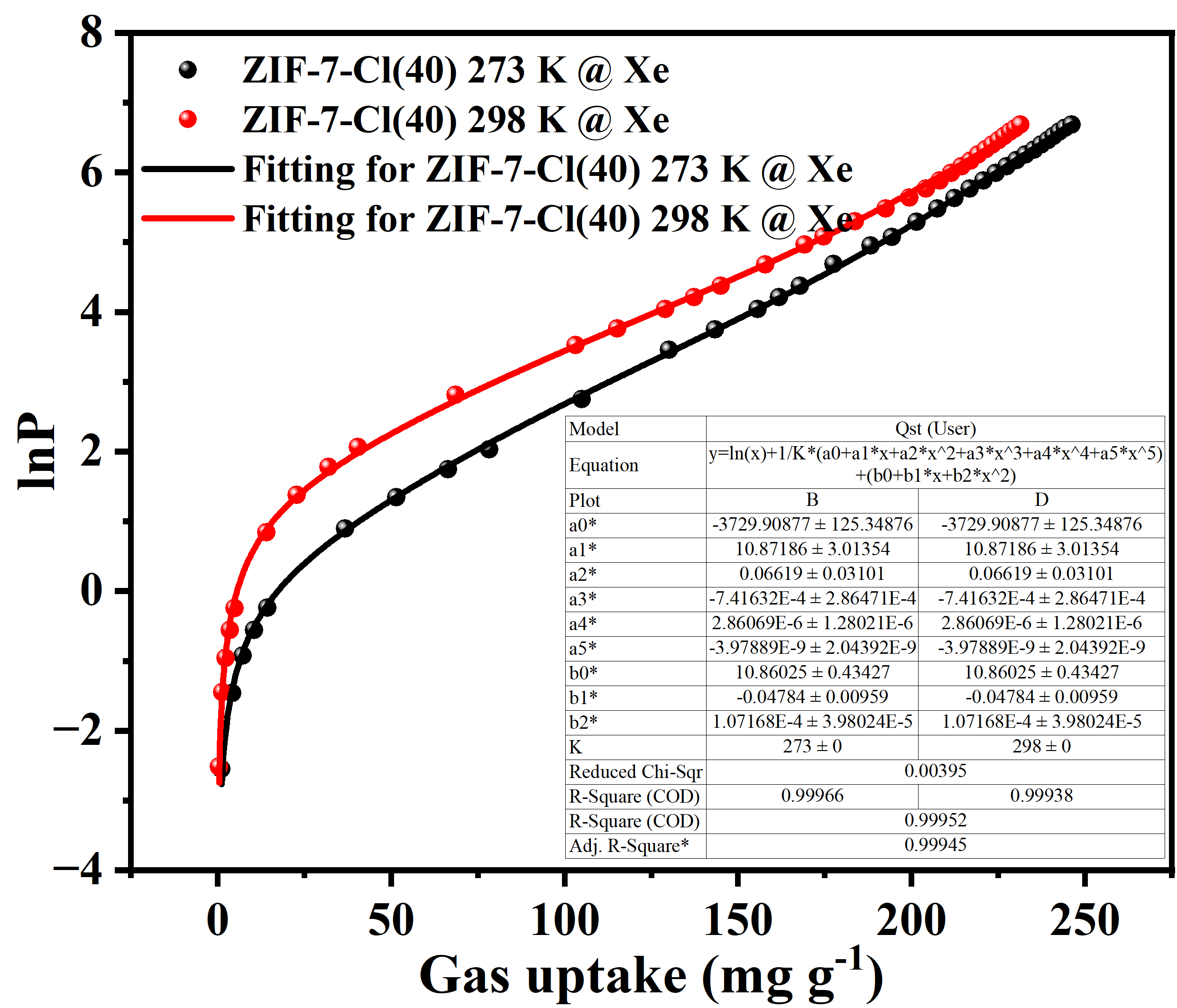


**Figure S68.** The virial fitting of Xe adsorption isotherm for ZIF-7-Cl(40).


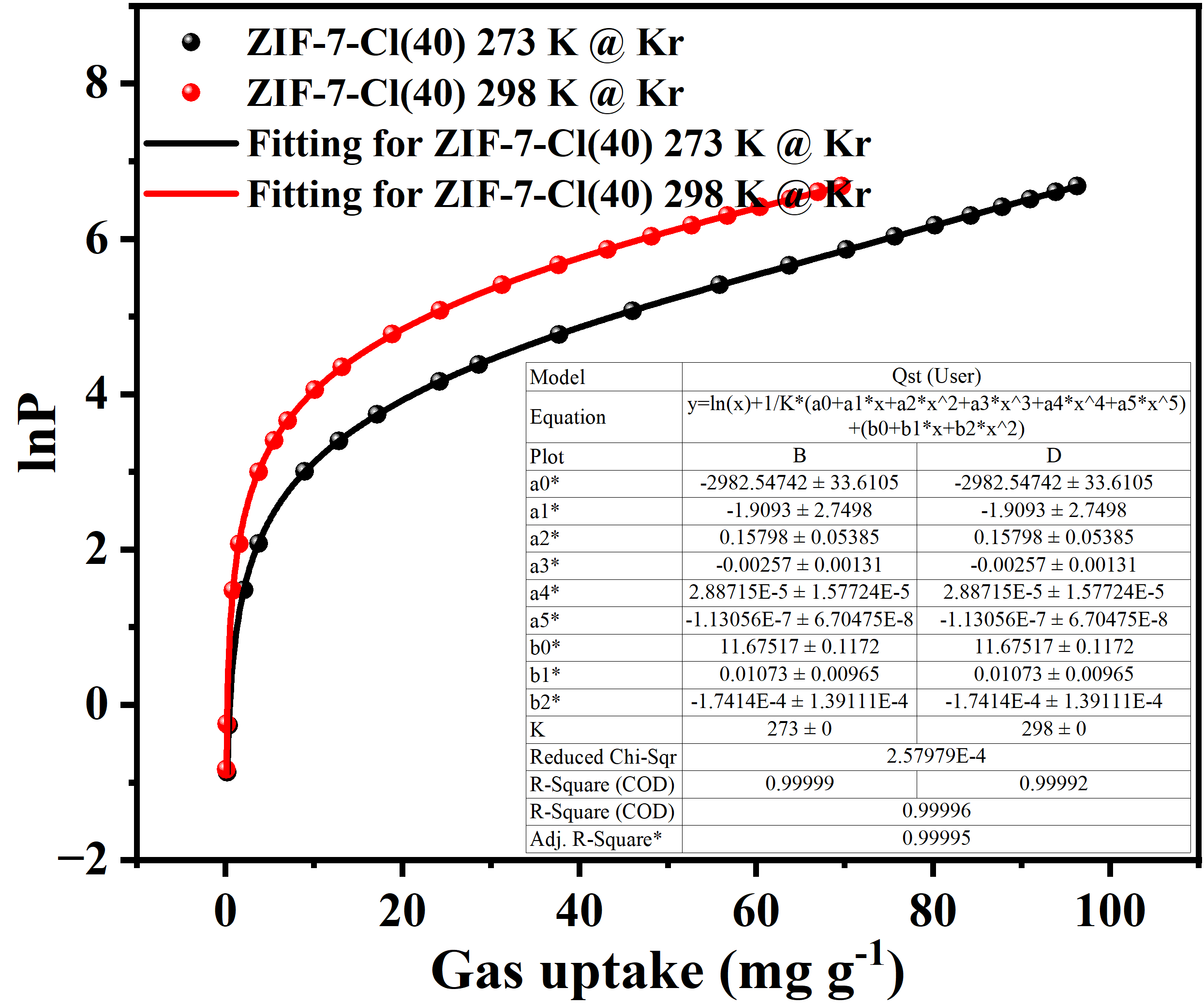


**Figure S69.** The virial fitting of Kr adsorption isotherm for ZIF-7-Cl(40).


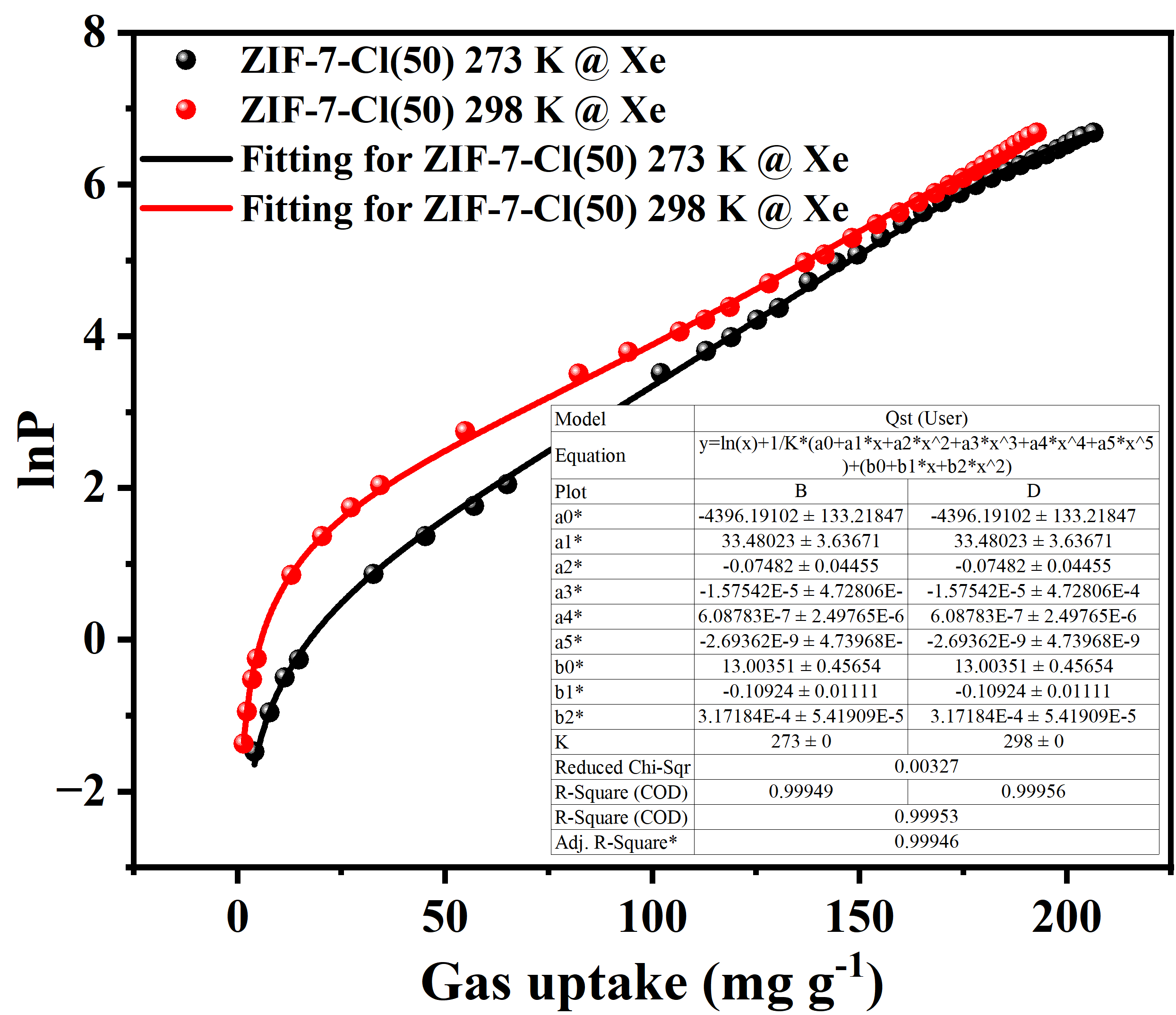


**Figure S70.** The virial fitting of Xe adsorption isotherm for ZIF-7-Cl(50).


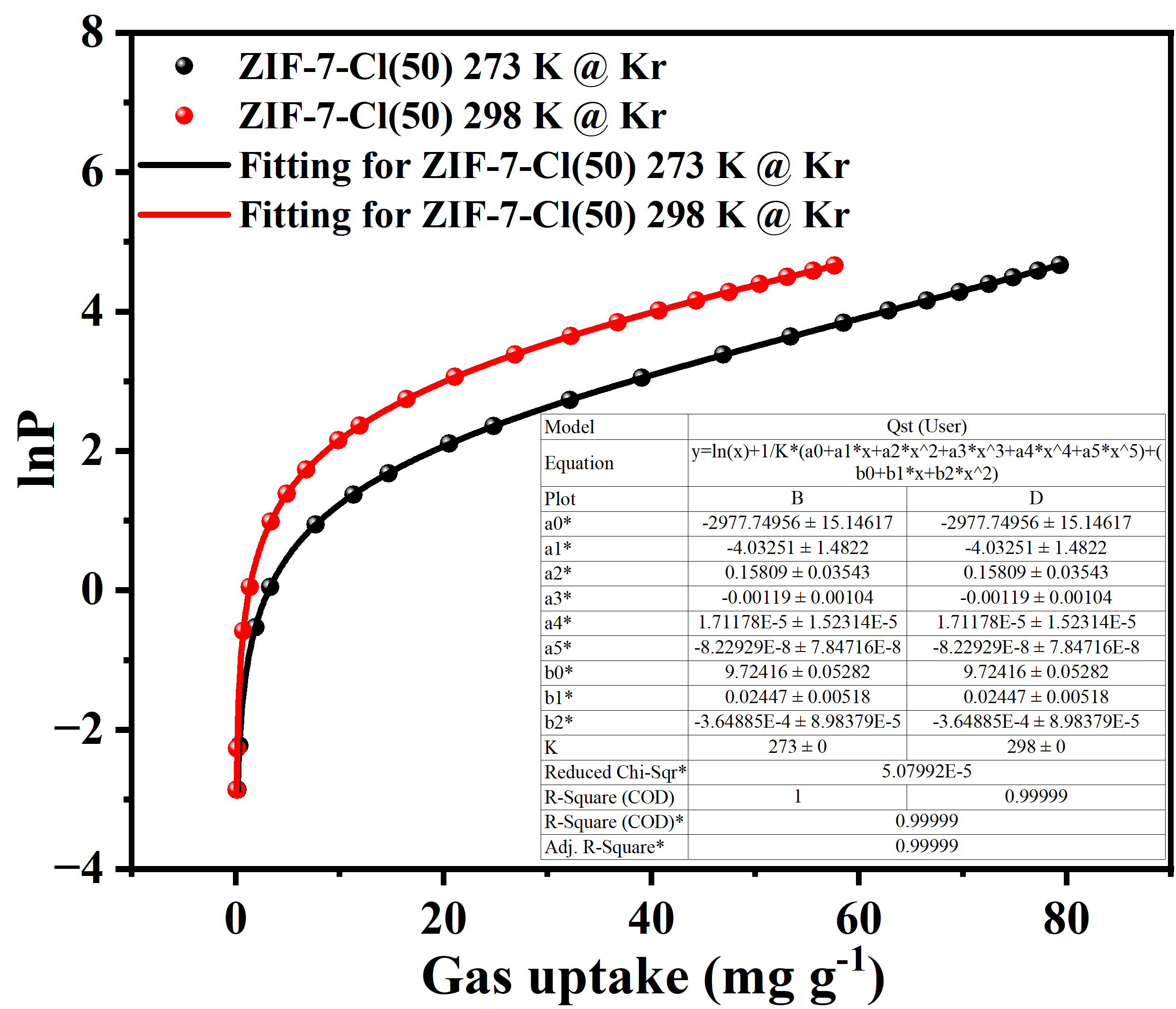


**Figure S71.** The virial fitting of Kr adsorption isotherm for ZIF-7-Cl(50).


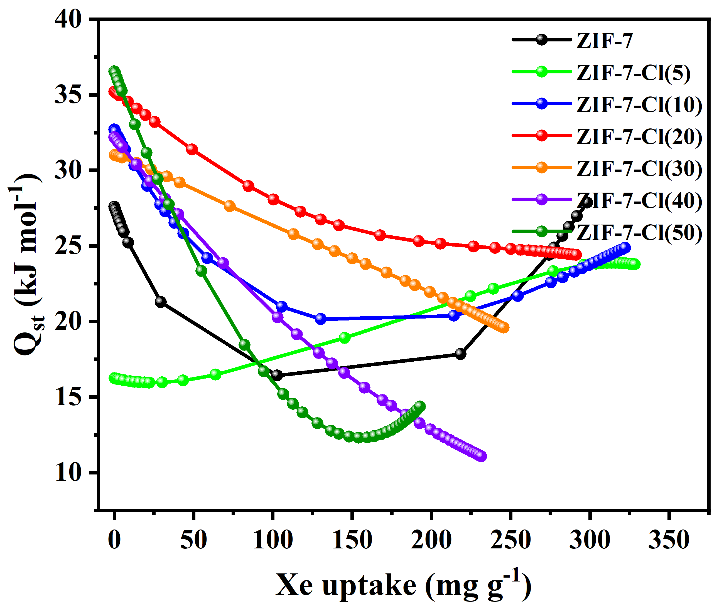


**Figure S72.** Coverage-dependent adsorption enthalpy of Xe for ZIF-7 and ZIF-7-Cl(x).


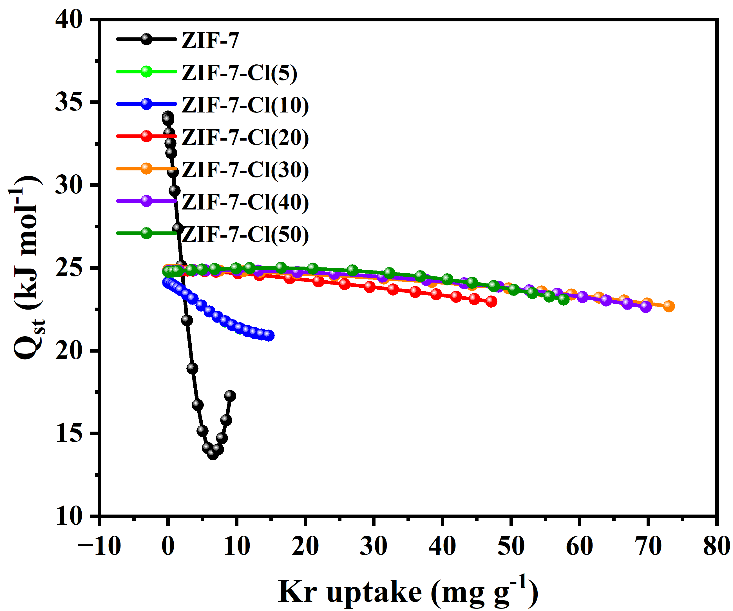


**Figure S73.** Coverage-dependent adsorption enthalpy of Kr for ZIF-7 and ZIF-7-Cl(x).


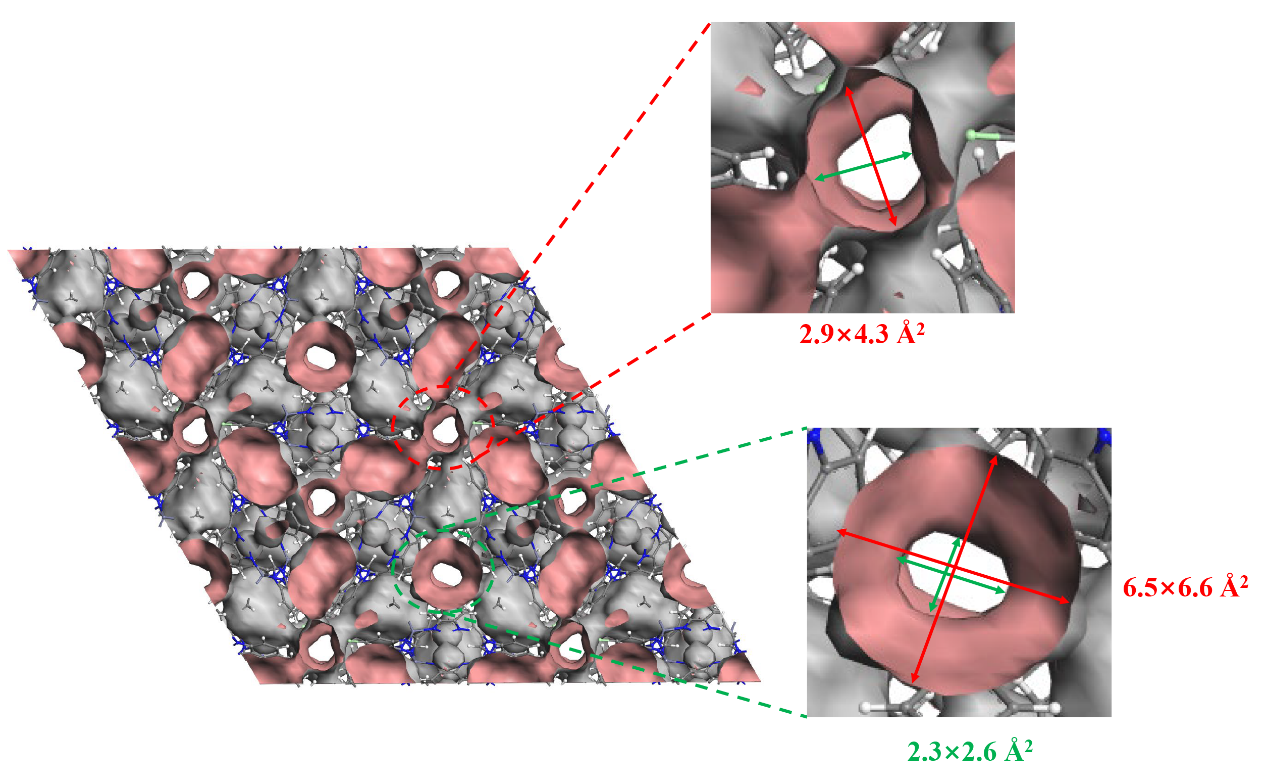


**Figure S74.** Visualized pore channels along the c-axis (Connolly radius of 1 Å determines Connolly surface).


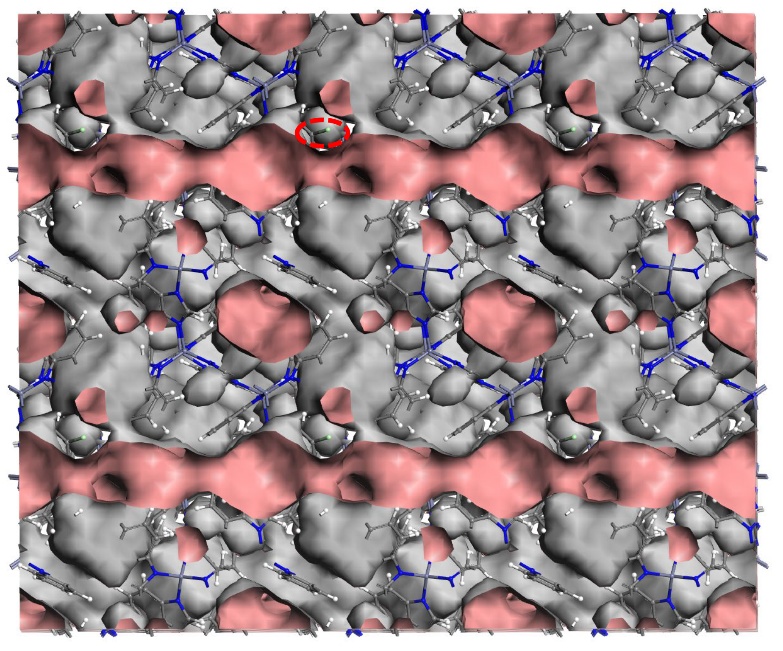


**Figure S75.** Channels along the c-axis side (Connolly radius of 1 Å determines Connolly surface).


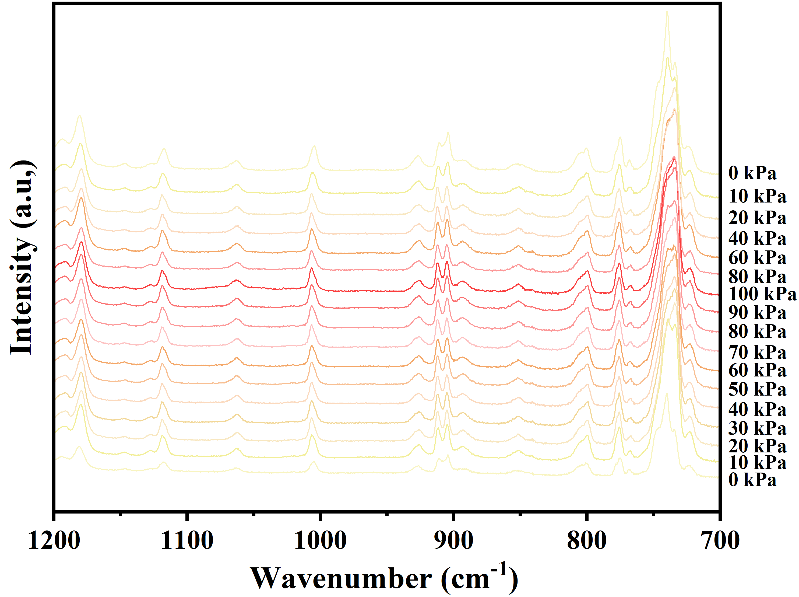


**Figure S76.** In-situ FT-IR spectra of ZIF-7-Cl(20) under different Xe loading pressures.


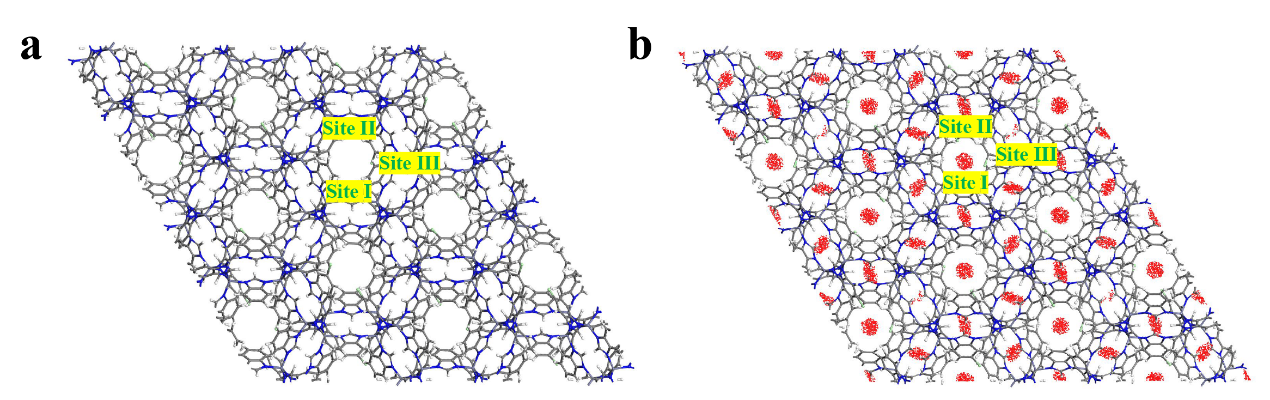


**Figure S77.** (a) Xe binding sites in the ZIF-7-Cl(20) framework; (b) Xe loading density under 1 kPa pressure simulated by GCMC to more intuitively represent the binding sites.


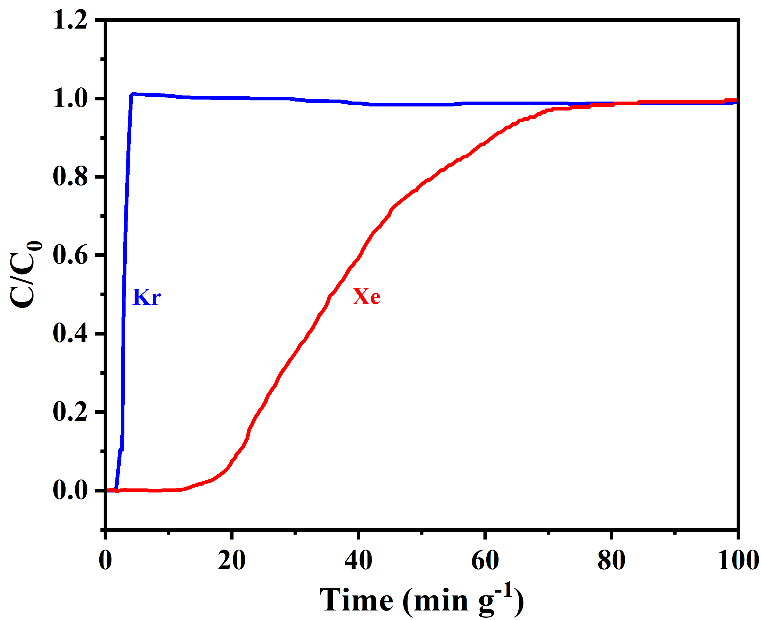


**Figure S78.** Dynamic gas breakthrough curves for the separation of a Xe/Kr (20/80, v/v) mixture on ZIF-7 at 298 K with a flow rate of 2 ml min^-1^.


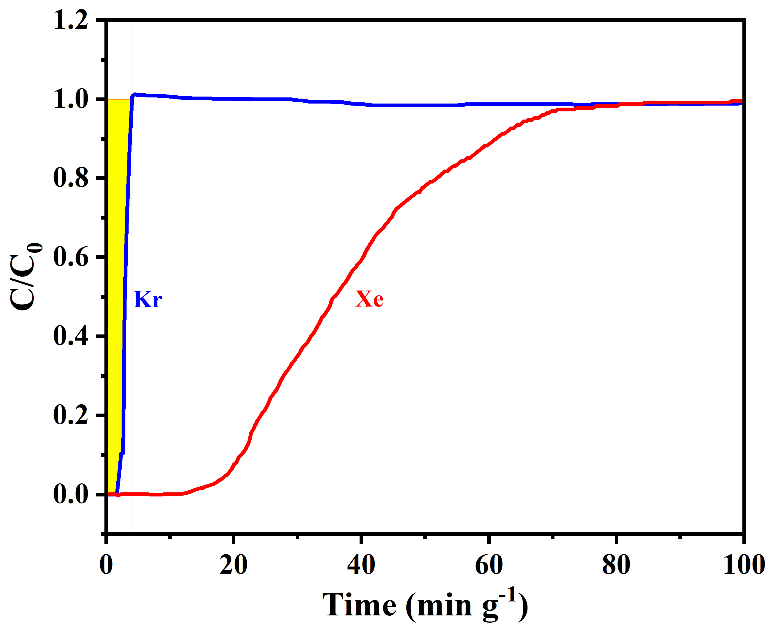


**Figure S79.** The breakthrough curves of Xe/Kr (20/80, v/v) on ZIF-7. The flow rate of Kr: q = 1.6 mL min^-1^, between the breakthrough point 0-t: the integration of the breakthrough curve gave loading Kr to be 4.8 cm^3^ g^-1^ $(Q=q\int_{t_{1}}^{t_{2}} \left[ C_{i}^{0}-C_{i}\left( t \right) \right]dt )$, so 0-t (S1) area above the entire breakthrough curves gave the maximum loading of ZIF-7-Cl to be 4.8 cm^3^ g^-1^.


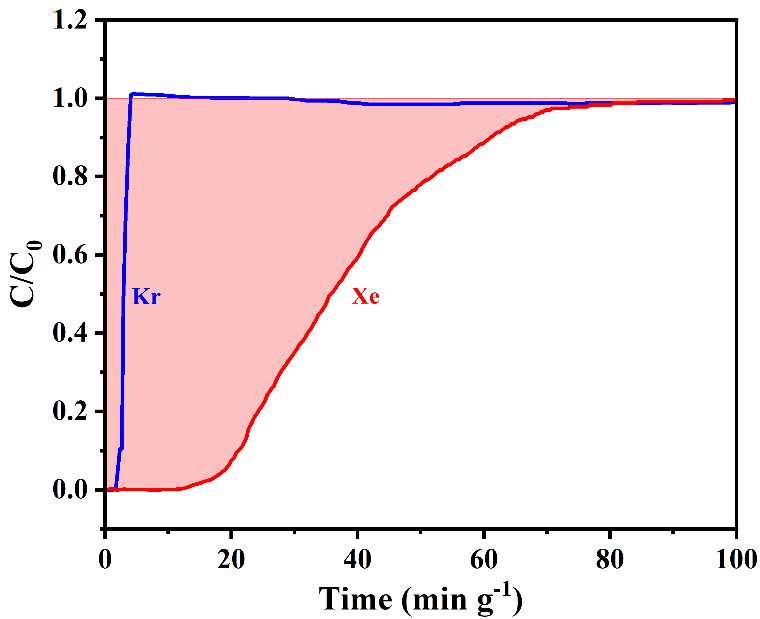


**Figure S80.** The breakthrough curves of Xe/Kr (20/80, v/v) on ZIF-7. The flow rate of Xe: q = 0.4 mL min^-1^, between the breakthrough point 0-t: the integration of the breakthrough curve gave loading Xe to be 15.4 cm^3^ g^-1^ $(Q=q\int_{t_{1}}^{t_{2}} \left[ C_{i}^{0}-C_{i}\left( t \right) \right]dt )$, so 0-t (S1) area above the entire breakthrough curves gave the maximum loading of ZIF-7 to be 15.4 cm^3^ g^-1^.


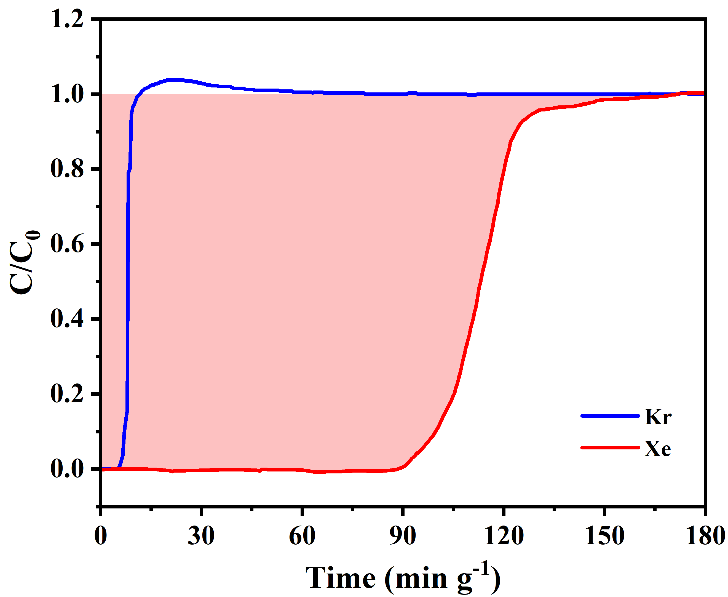


**Figure S81.** The breakthrough curves of Xe/Kr (20/80, v/v) on ZIF-7-Cl(20). The flow rate of Xe: q = 0.4 mL min^-1^, between the breakthrough point 0-t: the integration of the breakthrough curve gave loading Xe to be 45.4 cm^3^ g^-1^ $(Q=q\int_{t_{1}}^{t_{2}} \left[ C_{i}^{0}-C_{i}\left( t \right) \right]dt )$, so 0-t (S1) area above the entire breakthrough curves gave the maximum loading of ZIF-7-Cl(20) to be 45.4 cm^3^ g^-1^.


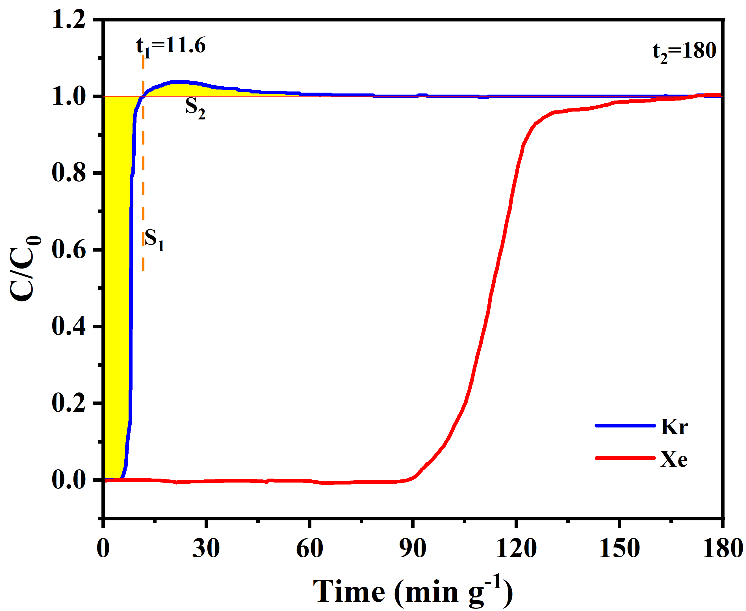


**Figure S82.** The breakthrough curves of Xe/Kr (20/80, v/v) on ZIF-7-Cl(20). The flow rate of Kr: q = 1.6 mL min^-1^, between the breakthrough point 0-t_1_: the integration of the breakthrough curve gave loading Xe to be 12.96 cm^3^ g^-1^ $(Q=q\int_{t_{1}}^{t_{2}} \left[ C_{i}^{0}-C_{i}\left( t \right) \right]dt )$ (S1). From t_1_ to t_2_, Kr partially flows out as the penetration experiment proceeds, and S2 represents the overflow amount in this stage, which is calculated as 1.66 cm^3^ g^-1^. 0-t_2_ (S1-S2) area above the entire breakthrough curves gave the maximum loading of ZIF-7-Cl(20) to be 11.3 cm^3^ g^-1^.


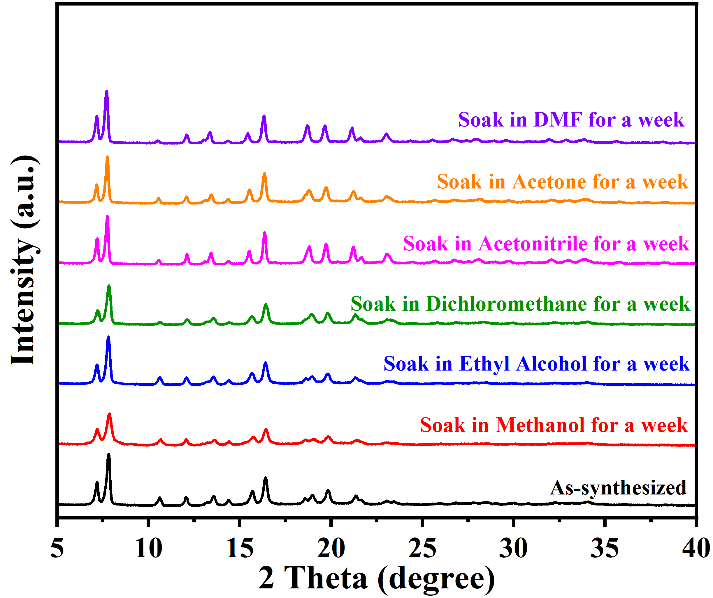


**Figure S83.** PXRD patterns of ZIF-7-Cl(20): as-synthesized, soak in MeOH, EtOH, DMF, Dichloromethane, Acetonitrile and Acetone for a week.


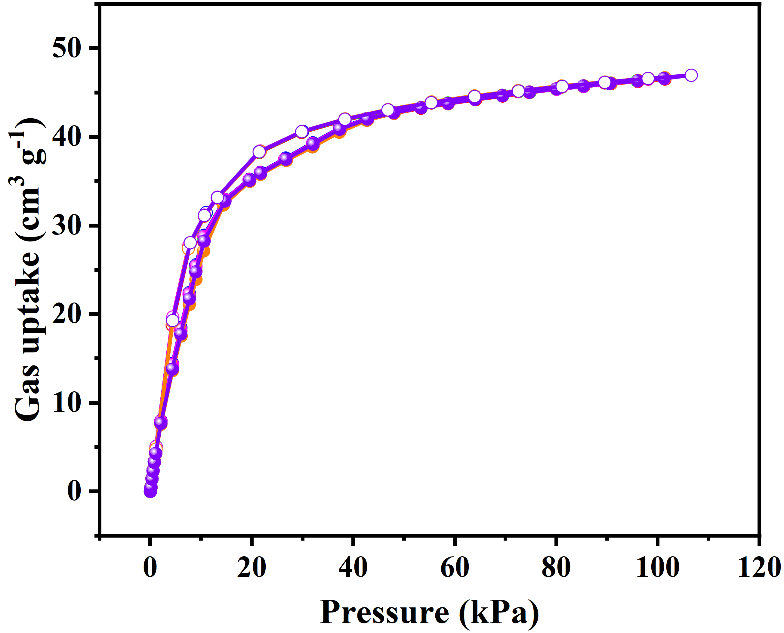


**Figure S84.** Six-cycle Xe adsorption-desorption curves of ZIF-7-Cl(20) at 298 K.

**Table S1.** The amount of the second ligand added during sample synthesis experiment and the actual value of the ^1^H NMR test results

| **Sample** | **Experiment (%)** | **Actual (%)** |
| --- | --- | --- |
| ZIF-7 | 0 | 0 |
| ZIF-7-Cl(5) | 5 | 7.4 |
| ZIF-7-Cl(10) | 10 | 10.7 |
| ZIF-7-Cl(20) | 20 | 17.3 |
| ZIF-7-Cl(30) | 30 | 28.5 |
| ZIF-7-Cl(40) | 40 | 36.6 |
| ZIF-7-Cl(50) | 50 | 47 |
| ZIF-7-Cl(100) | 100 | 100 |

**Table S2.** Elemental analysis results of ZIF-7 and ZIF-7-Cl(20).

| **Sample** | **C (wt%)** | **N (wt%)** | **H (wt%)** |
| --- | --- | --- | --- |
| ZIF-7 | 52.66 | 17.68 | 3.12 |
|  | 52.78 | 17.72 | 3.14 |
| ZIF-7-Cl(20) | 50.80 | 17.10 | 2.90 |
|  | 50.86 | 17.18 | 2.94 |

**Table S3.** Specific surface area calculated from 77K N_2_ and 195 K CO_2_ adsorption isotherms of ZIF-7 and ZIF-7-Cl(x).

| **Sample** | **Specific surface area (m^2^ g^-1^)** | | |
| --- | --- | --- | --- |
|  | **77K N_2_** | | **195K CO_2_** |
| ZIF-7 | 38 | 339 | |
| ZIF-7-Cl(5) | 119 | 300 | |
| ZIF-7-Cl(10) | 17 | 275 | |
| ZIF-7-Cl(20) | 19 | 236 | |
| ZIF-7-Cl(30) | 10 | 200 | |
| ZIF-7-Cl(40) | 9 | 203 | |
| ZIF-7-Cl(50) | 8 | 183 | |
| ZIF-7-Cl(100) | 9 | 8 | |

**Table S4.** Refined unit cell parameters of ZIF-7-Cl(20).

| **Name** | **ZIF-7-Cl(20)** |
| --- | --- |
| CCDC number | 2529844 |
| a (Å) | 22.7150 |
| b (Å) | 22.7152 |
| c (Å) | 15.7587 |
| α (deg) | 90 |
| β (deg) | 90 |
| γ (deg) | 120 |
| Volume (Å)^3^ | 7041.77 |
| Z | 2 |
| Rwp | 4.21% |
| Rp | 3.15% |
| Rexp | 2.49% |
| gof | 1.69 |
